# The impact of the COVID-19 pandemic on self-harm and suicidal behaviour: a living systematic review

**DOI:** 10.12688/f1000research.25522.1

**Published:** 2020-09-04

**Authors:** Ann John, Chukwudi Okolie, Emily Eyles, Roger T. Webb, Lena Schmidt, Luke A. McGuiness, Babatunde K. Olorisade, Ella Arensman, Keith Hawton, Nav Kapur, Paul Moran, Rory C. O'Connor, Siobhan O'Neill, Julian P.T. Higgins, David Gunnell

**Affiliations:** 1 Population Psychiatry, Suicide and Informatics, Swansea University, Swansea, UK; 2 Public Health Wales NHS Trust, Swansea, UK; 3 National Institute for Health Research Applied Research Collaboration West (NIHR ARC West) at University Hospitals Bristol NHS Foundation Trust, Bristol, UK; 4 Population Health Sciences, University of Bristol, Bristol, UK; 5 Division of Psychology and Mental Health, University of Manchester, Manchester, UK; 6 NIHR Greater Manchester Patient Safety Translational Research Centre, Manchester, UK; 7 School of Public Health and National Suicide Research Foundation, University College Cork, Cork, Ireland; 8 University Department of Psychiatry, Centre for Suicide Research, University of Oxford, Oxford, UK; 9 Oxford Health NHS Foundation Trust, Oxford, UK; 10 Greater Manchester Mental Health NHS Foundation Trust, Manchester, UK; 11 National Institute for Health Research Biomedical Research Centre at the University Hospitals Bristol NHS Foundation Trust and the University of Bristol, Bristol, UK; 12 Institute of Health & Wellbeing, University of Glasgow, Glasgow, UK; 13 School of Psychology, University of Ulster, Coleraine, UK

**Keywords:** COVID-19, Living systematic review, Suicide; Attempted suicide, Self-harm, Suicidal thoughts

## Abstract

**Background: **The COVID-19 pandemic has caused morbidity and mortality, as well as, widespread disruption to people’s lives and livelihoods around the world. Given the health and economic threats posed by the pandemic to the global community, there are concerns that rates of suicide and suicidal behaviour may rise during and in its aftermath. Our living systematic review (LSR) focuses on suicide prevention in relation to COVID-19, with this iteration synthesising relevant evidence up to June 7 ^th^ 2020.

**Method: ** Automated daily searches feed into a web-based database with screening and data extraction functionalities. Eligibility criteria include incidence/prevalence of suicidal behaviour, exposure-outcome relationships and effects of interventions in relation to the COVID-19 pandemic. Outcomes of interest are suicide, self-harm or attempted suicide and suicidal thoughts. No restrictions are placed on language or study type, except for single-person case reports.

**Results:** Searches identified 2070 articles, 29 (28 studies) met our inclusion criteria, of which 14 articles were research letters or pre-prints awaiting peer review. All articles reported observational data: 12 cross-sectional; eight case series; five modelling; and three service utilisation studies. No studies reported on changes in rates of suicidal behaviour. Case series were largely drawn from news reporting in low/middle income countries and factors associated with suicide included fear of infection, social isolation and economic concerns.

**Conclusions:  **A marked improvement in the quality of design, methods, and reporting in future studies is needed. There is thus far no clear evidence of an increase in suicide, self-harm, suicidal behaviour, or suicidal thoughts associated with the pandemic. However, suicide data are challenging to collect in real time and economic effects are evolving. Our LSR will provide a regular synthesis of the most up-to-date research evidence to guide public health and clinical policy to mitigate the impact of COVID-19 on suicide.

**PROSPERO registration:** CRD42020183326 01/05/2020

## Introduction

The COVID-19 pandemic is causing widespread societal disruption and loss of life globally. By the end of June 2020 over 10 million people had been infected and over 500,000 had died ( Worldometer, 2020). There are concerns about the impact of the pandemic on population mental health ( Holmes *et al*., 2020). These stem from the impact of the virus itself on people infected, as well as front-line workers caring for them ( Kisely *et al*., 2020), and on population mental health from the public health measures that have been implemented to minimise the spread of the virus – in particular physical distancing, leading to social isolation, disruption of businesses, services and education and threats to peoples’ livelihoods. Physical distancing measures have resulted in substantial rises in unemployment, falls in GDP and concerns that many nations will enter a prolonged period of deep economic recession.

There are concerns that suicide and self-harm rates may rise during and in the aftermath of the pandemic ( Gunnell *et al*., 2020; Reger *et al*., 2020). Time-series modelling indicated that the 1918-20 Spanish Flu pandemic, which caused well over 20 million deaths worldwide, led to a modest rise in the national suicide rate in the USA ( Johnson & Mueller, 2002; Wasserman, 1992). Likewise, there is evidence that suicide rates increased briefly amongst people aged over 65 years in Hong Kong during the 2003 SARS epidemic, predominantly amongst those with more severe physical illness and physical dependency ( Cheung *et al*., 2008).

The current context is, however, very different from previous epidemics and pandemics. The 2003 SARS epidemic was restricted to relatively few countries. Furthermore, during the 100-year period since the 1918-20 influenza pandemic, global and national health systems have improved, international travel and the speed of communication of information (and disinformation) have increased, antibiotics are available to treat secondary infection, and national economies have become more inter-dependent. The availability of the internet and technological advancement has made it far easier for people to communicate and engage in home working and home schooling. However, there are marked social inequalities in relation to access to technology and ability to stay safe and continue to work, within and between countries. Public health policies and responses, and the degree of access to technology to facilitate online clinical assessments and treatments differ greatly between countries.

Key concerns in relation to suicide prevention during the pandemic include: uncertainty regarding how best to assess and support people with suicidal thoughts and behaviours, whilst maintaining physical distancing; people who have attempted suicide may not attend hospitals because they are worried about contracting COVID-19 or being a burden on the healthcare system at this time; diminished access to community-based support; exposure to traumatic experiences; and an economic recession may have an adverse impact on suicide rates ( Chang *et al*., 2013; Stuckler *et al*., 2009). There have been increases in bereavement (with many being unusually complicated during the crisis), sales of alcohol ( Finlay & Gilmore, 2020) and domestic violence ( Mahase, 2020) – all risk factors for suicide ( Turecki *et al*., 2019); the insensitive or irresponsible media reporting of suicide deaths associated with COVID-19 may be harmful; and in some countries access to highly lethal suicide methods such as firearms and pesticides may rise ( Gunnell *et al*., 2020).

In the context of the COVID-19 pandemic there is likely to be a rapidly expanding research evidence base on its impact on suicide rates, and how best to mitigate such effects. It is therefore important that the best available knowledge is made rapidly available to policymakers, public health specialists and clinicians. To facilitate this, we are conducting a living systematic review focusing on suicide prevention in relation to COVID-19. Living systematic reviews are high-quality, up-to-date online summaries of research that are regularly updated, using efficient, often semi-automated, systems of production ( Elliott *et al*., 2014). This paper reports the first set of findings from the review, based on relevant articles identified up to June 7 ^th^ 2020.

## Aim

The overarching aim of the review is to identify and appraise any newly published evidence from around the world that assesses the impact of the COVID-19 pandemic on suicide deaths, suicidal behaviours, self-harm and suicidal thoughts, or that assesses the effectiveness of strategies to reduce the risk of suicide deaths, suicidal behaviours, self-harm and suicidal thoughts, resulting from the COVID-19 pandemic.

## Methods

**Figure 1.**
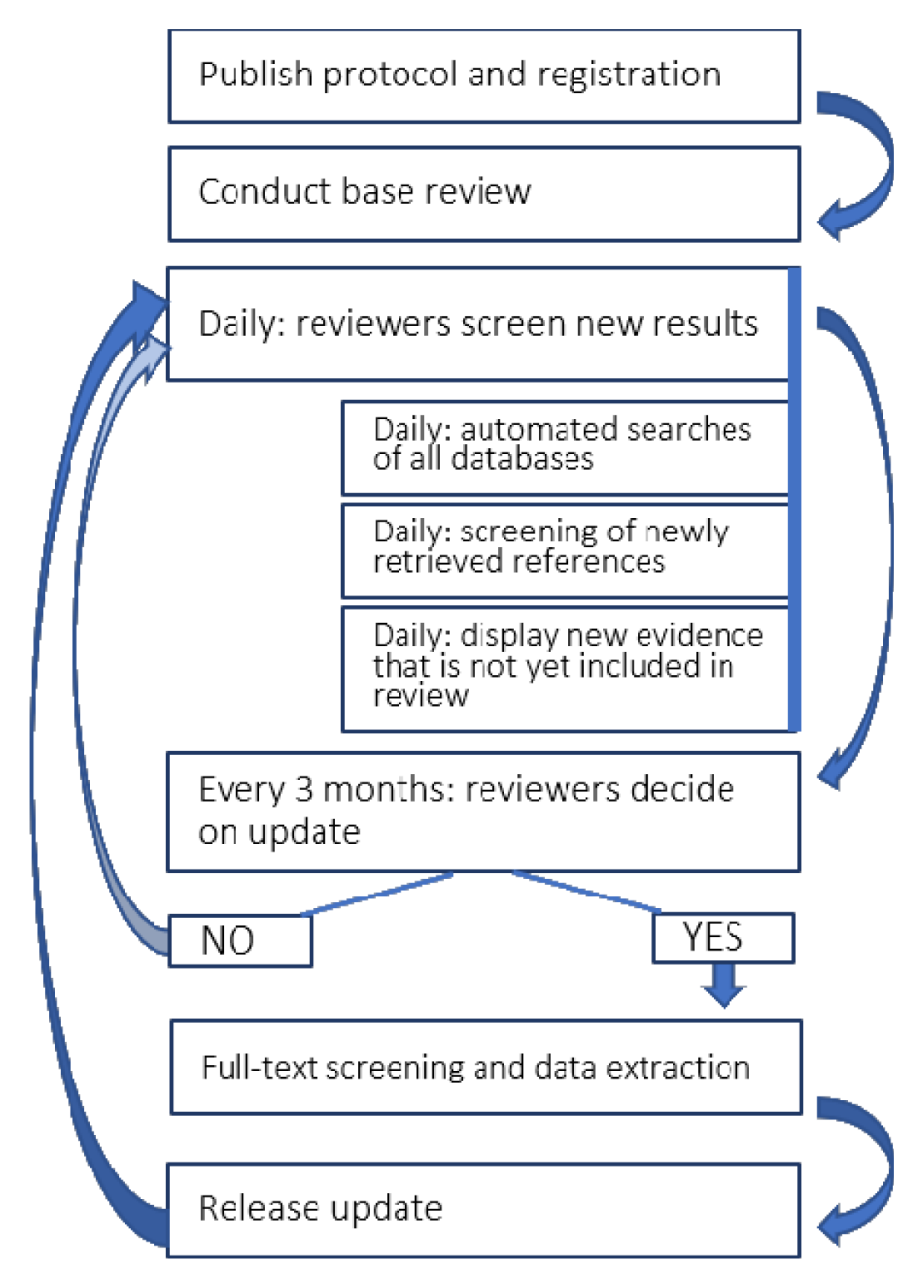
Workflow for updating the living systematic review review. The process will be supported using automation technology and at three-monthly intervals the team will update the published version of the review. The latest and previous versions of this figure are available as extended data ( John & Schmidt, 2020).

This living systematic review ( Figure 1) follows published guidance for such reviews and for how expedited ‘living’ recommendations should be formed where relevant ( Akl *et al*., 2017; Elliott *et al*., 2017). The review was prospectively registered (PROSPERO ID CRD42020183326; registered on 1 May 2020). An overview of our living review process is provided in Figure 1. A protocol ( John *et al*., 2020a) was published in line with the Preferred Reporting Items for Systematic Review and Meta-Analysis Protocols guideline ( Moher *et al*., 2015). Since publication of our protocol we have amended our methodology to: 1) search additionally the PsyArXiv and SocArXiv open access paper repositories; 2) include modelling studies within the scope of our review (e.g. to predict the likely impact of the pandemic on suicide rates); and 3) update our research questions to include adult self-neglect and parental neglect and fear of losing livelihood.

## Eligibility criteria

Study participants may be adults or children of any ethnicities living in any country. Outcomes of interest are:

1. Deaths by suicide
2. Self-harm (intentional self-injury or self-poisoning regardless of motivation and intent) or attempted suicide (including hospital attendance and/or admission for these reasons)
3. Suicidal thoughts/ideation

Studies must address one of the following research questions:

(i) What is the prevalence/incidence?

- Prevalence/incidence of each outcome during pandemic (including modelling studies)

(ii) What is the comparative prevalence/incidence?

- Prevalence/incidence of each outcome during pandemic vs not during pandemic

(iii) What are the effects of interventions?

- Effects of public health measures to combat COVID-19 (including physical distancing, school closures, interventions to address loss of income, interventions to tackle domestic violence) on each outcome
- Effects of changed and new approaches to clinical management of (perceived) elevated risk of self-harm or suicide risk on each outcome (any type of intervention is relevant)

(iv) What are the effects of other exposures?

- Impact of media portrayal of each outcome and misinformation attributed to the pandemic on each outcome
- Impact of bereavement from COVID-19 on each outcome
- Impact of any COVID-19 related behaviour changes (domestic violence, alcohol, adult self-neglect, parental neglect, cyberbullying, isolation) on each outcome
- Impact of COVID-19-related workload on crisis lines on each outcome
- Impact of infection with COVID-19 (self or family member) on each outcome
- Impact of changes in availability of analgesics, firearms and pesticides on each outcome (method-specific and overall suicide rates)
- Impact of COVID-19 related socio-economic exposures (changes in fiscal policy; recession/depression: unemployment, debt, fear of losing livelihood, deprivation at the person-, family- or small-area level) on each outcome
- Impact on health and social care professionals: the stigma of working with COVID-19 patients or the (perceived) risk of infection/being a ‘carrier’, as well as work-related stress on each outcome
- Impact of changes in/reduced intensity of treatment for patients with mental health conditions, in particular those with severe psychiatric disorders.
- Impact of any other relevant exposure on our outcomes of interest.

## Qualitative research

We include any qualitative research addressing perceptions or experiences around each outcome in relation to the COVID-19 pandemic (e.g. stigma of infection, isolation measures, complicated bereavement, media reporting, experience of delivering or receiving remote methods of self-harm/suicide risk assessment or provision of treatment; experience of seeking help for individuals in suicidal crisis); narratives provided for precipitating factors for each outcome.

No restrictions were placed on the types of study design eligible for inclusion, except for the exclusion of single-person case reports. Pre-prints were re-assessed at the time of publication and most current version included. There was no restriction on language of publication. We will draw on a combination of internet-based translation systems and network of colleagues to translate evidence in a language other than English.

## Identification of eligible studies

We searched the following electronic databases: PubMed; Scopus; medRxiv, bioRxiv; the COVID-19 Open Research Dataset (CORD-19) by Semantic Scholar and the Allen Institute for AI, which includes relevant records from Microsoft Academic, Elsevier, arXiv and PMC; and the WHO COVID-19 database. A sample search strategy (for PubMed) appears in Box 1 from 1 ^st^ Jan 2020 to June 7 ^th^ 2020. We have developed a workflow that automates daily searches of these databases, and the code supporting this process can be found at https://github.com/mcguinlu/COVID_suicide_living. Searches are conducted daily via PubMed and Scopus application programme interface and the bioRxiv and medRxiv RSS feeds. Conversion scripts for the daily updated WHO and the weekly updated CORD-19 corpus are used to collect information from the remaining sources. The software includes a systematic search function based on regular expressions to search results retrieved from the WHO, CORD-19 and preprint repositories (search strategy available in extended data ( John & Schmidt, 2020)). Our review is ongoing and we continue to investigate the use of other databases and to capture articles made available prior to peer review and assess eligibility and review internally. We therefore included PsyArXiv and SocArXiv repositories in our search strategy via their own open access platforms as we developed our automated system. For this version of the living review, Psy- and SocArXiv searches were carried out retrospectively on the 12 ^th^ of June, using a publication date filter for Jan 1 ^st^ 2020 – June 7 ^th^ 2020.

A two-stage screening process was undertaken to identify studies meeting the eligibility criteria. First, two authors (either CO or EE) assessed citations from the searches and identified potentially relevant titles and abstracts. Second, either DG, AJ or RW assessed the full texts of potentially eligible studies to identify studies to be included in the review. This process was managed via a custom-built online platform (Shiny web app, supported by a MongoDB database). The platform allowed for data extraction via a built-in form.

**Box 1. Search terms for PubMed**

((selfharm*[TIAB] OR self-harm*[TIAB] OR selfinjur*[TIAB] OR self-injur*[TIAB] OR selfmutilat*[TIAB] OR self-mutilat*[TIAB] OR suicid*[TIAB] OR parasuicid*[TIAB) OR (suicide[TIAB] OR suicidal ideation[TIAB] OR attempted suicide[TIAB]) OR (drug overdose[TIAB] OR self?poisoning[TIAB]) OR (self-injurious behavio?r[TIAB] OR self?mutilation[TIAB] OR automutilation[TIAB] OR suicidal behavio?r[TIAB] OR self?destructive behavio?r[TIAB] OR self?immolation[TIAB])) OR (cutt*[TIAB] OR head?bang[TIAB] OR overdose[TIAB] OR self?immolat*[TIAB] OR self?inflict*[TIAB]))) AND ((coronavirus disease?19[TIAB] OR sars?cov?2[TIAB] OR mers?cov[TIAB]) OR (19?ncov[TIAB] OR 2019?ncov[TIAB] OR n?cov[TIAB]) OR ("severe acute respiratory syndrome coronavirus 2" [Supplementary Concept] OR "COVID-19" [Supplementary Concept] OR COVID-19 [tw] OR coronavirus [tw] OR nCoV[TIAB] OR HCoV[TIAB] OR ((virus*[Title] OR coronavirus[Title] OR nCoV[Title] OR infectious[Title] OR HCoV[Title] OR novel[Title])AND (Wuhan[Title] OR China[Title] OR Chinese[Title] OR 2019[Title] OR 19[Title] OR COVID*[Title] OR SARS-Cov-2[Title] OR NCP*[Title]) OR “Coronavirus”[MeSH]))))

## Data collection and assessment of risk of bias

One author (DG, AJ or RW) extracted data from each included study using a piloted data extraction form (see extended data ( John & Schmidt, 2020)), and the extracted data were checked by one other author (AJ, or EE where AJ extracted data). Disagreements were resolved through discussion, and where this failed, by referral to a third reviewer (KH, NK or PM). Irrespective of study design, data source and outcome measure examined, the following basic data were extracted: citation; study aims and objectives; country/setting; characteristics of participants; methods; outcome measures (related to self-harm / suicidal behaviour and COVID-19); key findings; strengths and limitations; reviewer’s notes. For articles where causal inferences are made - i.e. randomised or non-randomised studies examining the effects of interventions or aetiological epidemiological studies of the effects of exposures – we used a suitable version of the ROBINS-I or ROBINS-E tool to assess risk of bias as appropriate based on the research question and study design ( Morgan *et al*., 2017; Sterne *et al*., 2016).

## Data synthesis

We synthesised studies according to themes based on research questions and study design, using tables and narrative. Results were synthesised separately for studies in the general population, in health and social care staff and other at-risk occupations, and in vulnerable populations (e.g. people of older age or those with underlying conditions that predispose them to becoming severely ill or dying after contracting COVID-19). Where multiple studies addressed the same research questions, we assessed whether meta-analysis is appropriate and would conduct it where suitable, following standard guidance available in the Cochrane Handbook ( Deeks *et al*., 2019). The current document is the first iteration of our review. We have not considered it appropriate to combine any results identified so far in a meta-analysis.

## Living review method

Details of the living review method, justification for its use and our transition plan are provided in our protocol ( John *et al*., 2020a). We plan to maintain the review in living mode for at least 12 months, from publication of the protocol (25 ^th^ June 2020). We will undertake monthly screening and consider full updates at least every three months. We will extend the living mode at 6-monthly intervals if evidence is still being published regularly. We anticipate an end to the living phase of the review at most 24 months after initiation, at which point we plan to publish the cumulated evidence in the form of a standard systematic review. Any decision to update the review more or less frequently will depend on the likely impact of the new evidence on the conclusions of the review. Impactful evidence may be (i) evidence that affects policy and/ or (ii) substantial, high-quality research studies (e.g. a randomised trial or population-based observational cohort study). Since we have not as yet identified any new evidence that impacts on the conclusions of this review we next update will include studies up to the 7 ^th^ of October 2020 after four months.

## Results

**Figure 2.**
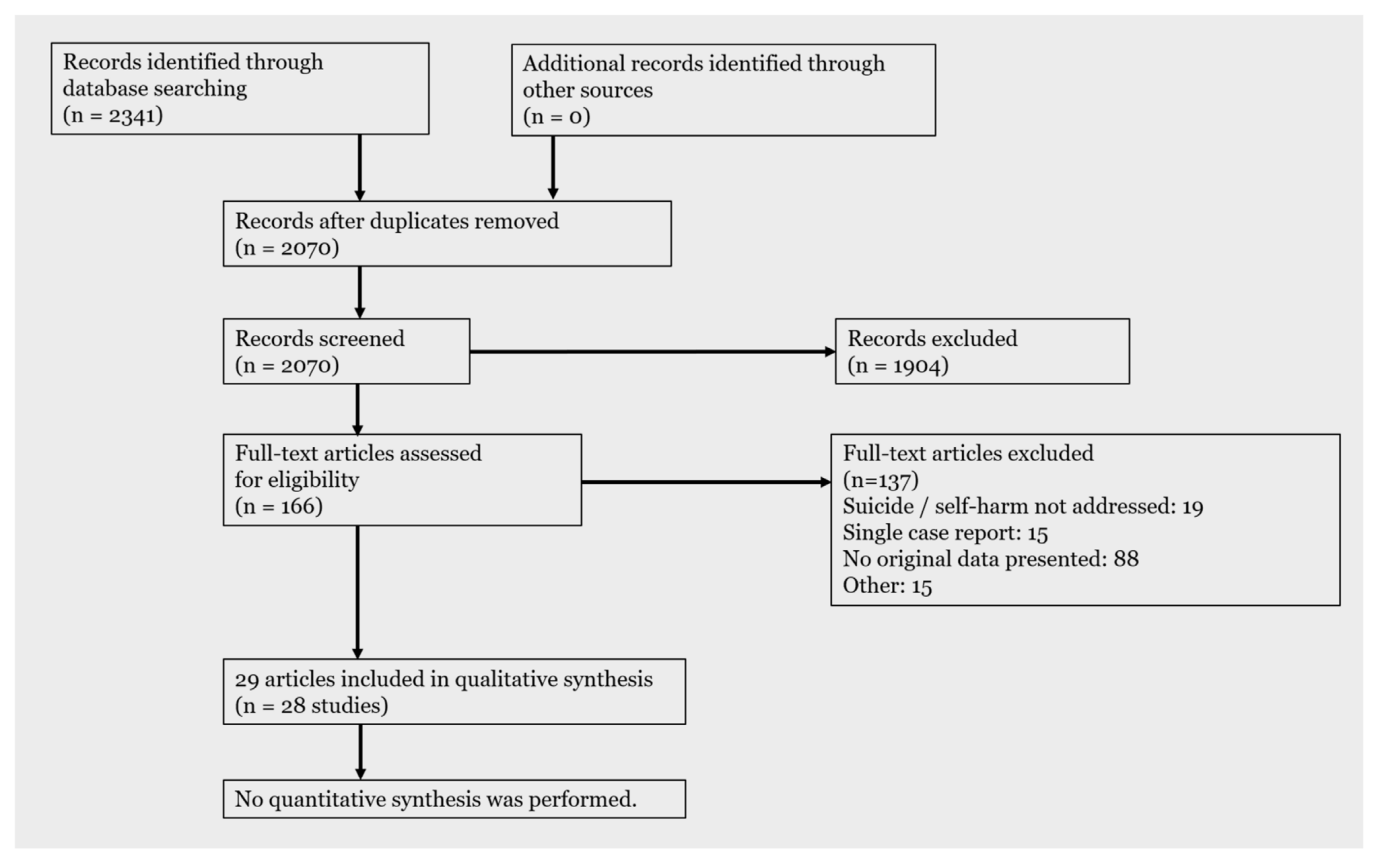
PRISMA flow diagram. The latest and previous versions of this figure are available as extended data ( John & Schmidt, 2020).

**Figure 3.**
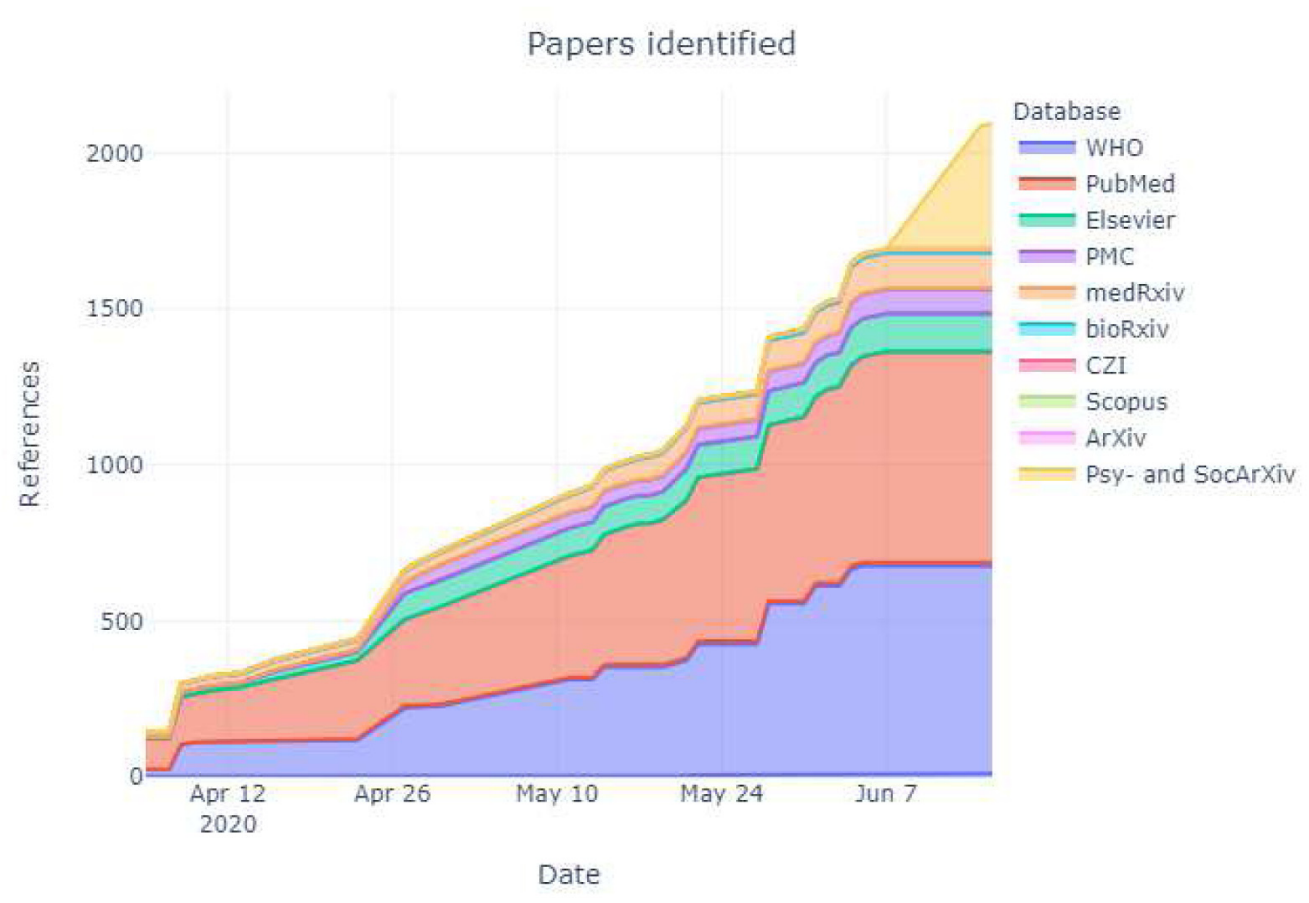
Number of articles identified by database and respository over time. The latest and previous versions of this figure are available as extended data ( John & Schmidt, 2020).

**Figure 4.**
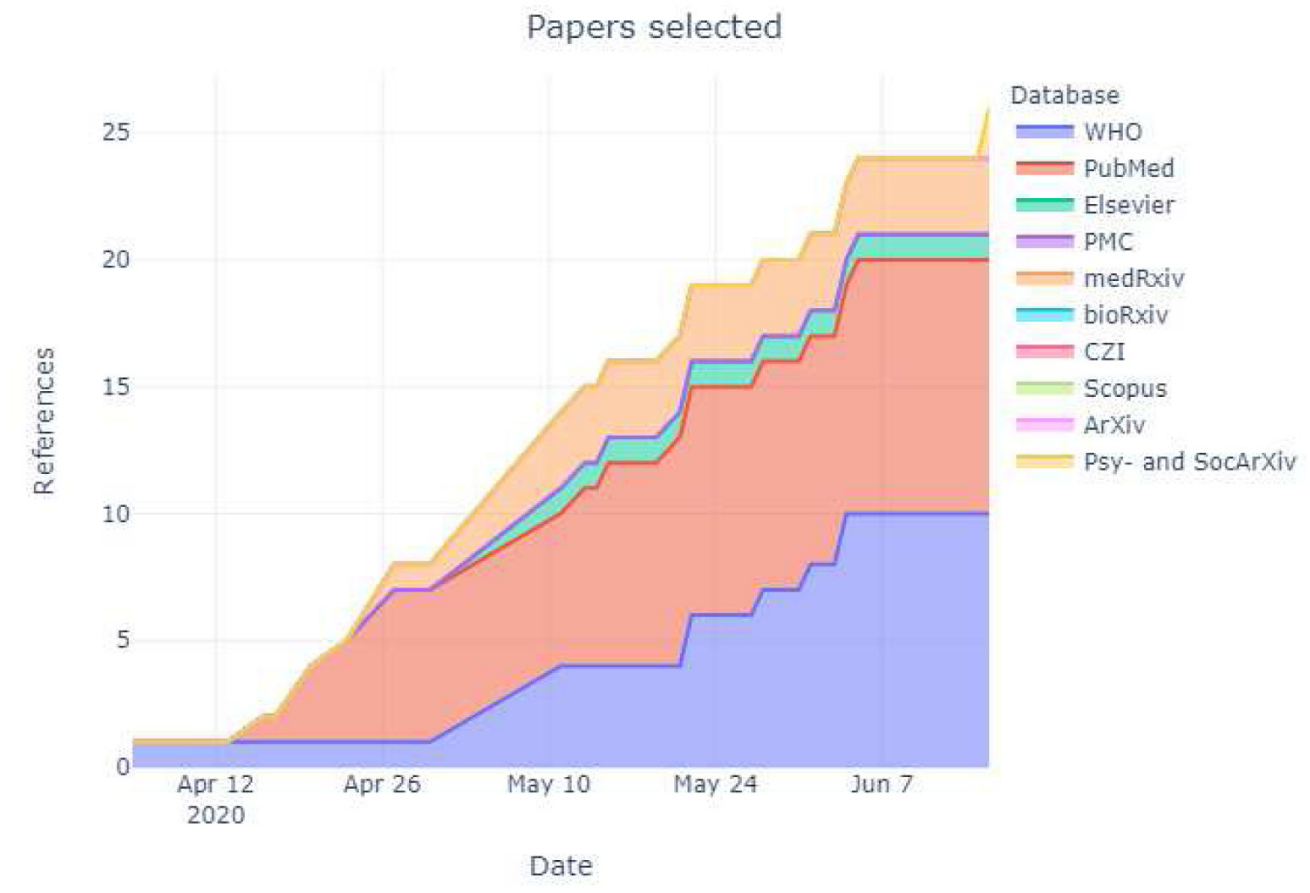
Number of articles selected by database and respository over time. The latest and previous versions of this figure are available as extended data ( John & Schmidt, 2020).

In total, 2070 citations were identified by 7 June 2020 from all electronic searches, after duplicates were removed ( Figure 2). The cumulative numbers of articles over time that were identified by the search and included in the review are shown in Figure 3 and Figure 4.

### Description of included studies

We included 29 articles in the review, describing 28 independent studies. In total, six studies spanned several countries or were worldwide, including those using online Amazon Mechanical Turk survey samples; six were from the United States; four from China; two from India; one each from Australia, Bangladesh, Canada, Germany, Greece, Pakistan, Spain, France and Switzerland. All articles were based on observational studies: eight were case series with a sample of two or more; 13 were cross sectional surveys (12 independent populations); five were modelling studies; and three were service utilisation studies. Studies are summarised by these study types in Table 1, Table 2, Table 3 and Table 4. Roughly half (n=14) of the articles did not appear to have been peer reviewed. Ten articles were published as research letters to the Editor, four as pre-prints before peer review and in seven others there was a short time (<7 days) between submission and acceptance.

**Table 1.** Summary of included case series. The latest and previous versions of this table are available as extended data ( John & Schmidt, 2020).

Authors	Geography	Data used	Outcome	Conclusions	Comment/ Limitations
Bhuiyan *et al.,* 2020	Bangladesh	News reports of COVID- 19 related suicide deaths (n=8)	Suicide death	Job loss, debt and difficulties obtaining food because of financial difficulties reported in all cases	Small sample size (n=8) Data drawn from news reports which depend on the reliability and extensiveness of data available to journalists. Representativeness of the cases unclear Letter to editor, probably not peer reviewed.
Buschmann & Tsokos, 2020	Germany	Case series of 10 individuals identified at autopsy who died by suicide during the pandemic up to March 25th 2020	Suicide death	All had pre-existing mental health issues. No evidence of COVID-19.	It is unclear what circumstances of the deceased persons were brought about directly due to the COVID-19 crisis. Letter to editor, probably not peer reviewed.
Dsouza *et al.,* 2020	India	News reports (n=69) of COVID-19 related suicide deaths including n=72 cases, 63 males, age 19-65 years from March to May 24, 2020.	Suicide death	The most common reported factors were: 1) Fear of infection (n=21) 2) Financial crisis (n=19) 3) COVID-19 related stress (n=9) 4) Positive test for COVID-19 (n=7) 5) Isolation related issues (n=5) 6)Social boycott (n=4) 7) Migrant unable to return home (n=3)	This is the largest case series of suicide deaths, which also excluded reports of deaths reported more than once. Data drawn from news reports which depend on the reliability and extensiveness of data available to journalists. Representativeness of the cases unclear Letter to editor, probably not peer reviewed.
Mamun & Ullah, 2020	Pakistan	News reports of COVID- 19 related suicide deaths in Pakistan (n=12, a further 4 reports of suspected suicide were not presented), January 2020 to end of April 2020.	Suicide death	Economic concerns reported in 8/12 cases, and fear of infection in the remaining 4. There were 13 other reports of suicides (and attempted suicide) during this period not reported to be linked to COVID-19.	Highlights the potential importance of the economic impact of COVID-19 and/or public health measures on influencing suicide in low- and middle-income countries. Data drawn from news reports which depend on reliability and extensiveness of data available to journalists Representativeness of the cases unclear
Griffiths & Mamun, 2020	Global -Bangladesh, India, Malaysia, USA	News reports of couples (n=6, one couple made suicide attempt, one murder suicide) engaging in COVID- 19-related suicidal behaviour identified via Searches of seven English- Indian online papers from March to May 24	Suicide attempt and/or death (couples)	Details several potential reasons: 1) Fear of infection; 2) Money problems (due to recession associated with lockdowns); 3) Harassment or victimisation by others due to (possibly perceived) infection status 4) Stress of being in isolation or quarantine 5) Uncertainty of when the pandemic will end	Small sample size (n=6) One of the only papers to report on suicide pacts. Data drawn from news reports which depend on reliability and extensiveness of data available to journalists. Representativeness of the cases unclear Letter to editor, probably not peer reviewed.
Sahoo *et al.,* 2020	India	Clinical case reports of COVID-19 related suicide attempts (n=2) presenting to the ED	Suicide attempts	Both cases are related to the fear and stigma of COVID-19. One case was ordered to self-isolate due to being in contact with a known case.	Small sample size (n=2) Letter to editor, probably not peer reviewed.
Thakur & Jain, 2020	World	News reports (n=7) of COVID-19 related suicide deaths	Suicide deaths	Identified 4 types of suicide risks: 1) Social isolation; 2) Economic; 3) Stress in health professionals; 4) Stigma	Small sample size (n=7) Data drawn from news reports which depend on reliability and extensiveness of data available to journalists. Representativeness of the cases unclear
Valdés-Florido *et al.,* 2020	Spain	Patients admitted to two hospitals in Spain with reactive psychoses in the context of the COVID-19 crisis during the first two weeks of lockdown (n=4)	Suicide attempts	Stress from the pandemic thought to have triggered reactive psychoses in four patients two of whom presented with severe suicidal behaviour	Small sample size (n=4)

**Table 2.** Summary of cross-sectional surveys. The latest and previous versions of this table are available as extended data ( John & Schmidt, 2020).

Authors	Geography	Data used	Outcome	Conclusions	Comment/ Limitations
Ammerman *et al.,* 2020	USA	General population recruited via Amazon Mechanical Turk (n=970), April 3-4, 2020 Mean age 36.43 years old (SD = 11.02, Range = 18 - 74). 56.30% of participants ( *n* = 511) male; 76.4% white	Suicidal thoughts Suicide attempts Measured by two items from the SITBI questionnaire to assess presence ( *yes*/ *no*) of past-month active suicidal ideation (i.e., “In the past month, have you had thoughts of killing yourself?”) and past- month suicide attempt (i.e., “In the past month, have you attempted to kill yourself?”).	Associations with **suicidal thoughts**(controlling for age and ethnicity): Protective effect of social distancing (OR 0.86 CI 0.78, 0.94); General distress related to COVID-19 1.14 (1.02; 1.27); concerns about physical safety: 1.14 (1.03; 1.26); Mental Health impact of social distancing measures: 1.08 (0.99, 1.19). Associations for **suicide attempts** (also controlling for sex and suicidal thoughts) report social distancing (OR 1.30 CI 1.03, 1.63); General distress 1.55 (1.20; 1.99); Physical safety concerns: 1.79 (1.36; 2.35); Mental Health impact 1.37 (1.11, 1.70).	The data are cross- sectional; no baseline pre-COVID-19 measures Questionnaire measures: Convenience sample Response rates unclear. The models for suicide attempts control for suicidal thoughts (along causal pathway), so they cannot be interpreted. Pre-print, not peer reviewed
Bryan *et al.,* 2020	USA	Qualtrics, online survey platform that maintains a database of several million U.S. residents who have volunteered to participate in periodic survey-based research. Quota sampling (age, sex, ethnicity), general population 18+ (n= 10,625). March 18, 2020 – April 2, 2020	Suicidal thoughts Suicide attempts From Self- Injurious Thoughts and Behaviors Interview (SITBI) questionnaire (Nock *et al.,* 2007)	Participants with past-month suicide ideation who were subject to large gatherings bans were significantly less likely to report a suicide attempt in the prior month (OR=0.39, 95% CI=0.17-0.88, p=.024). The likelihood of past-month suicide attempt was significantly increased among those endorsing concerns about a life-threatening illness or injury of a close friend or family member (OR=2.26, 95% CI=1.48-3.46, p<.001) but was decreased among those endorsing an unexpected bill or expense that cannot be easily afforded (OR=0.41, 95% CI=0.24-0.70, p=.001). In the subset of participants reporting past-month suicide ideation (n=489), only life-threatening illness or injury of a close friend or family member was associated with significantly increased likelihood of past-month suicide attempt (OR=3.87, 95% CI=2.14-6.99, p<.001). No evidence of an increased risk of suicidal thoughts or attempts in respondents subject to stay at home orders. Results did not support hypothesis that physical distancing measures were associated with suicide ideation or attempt.	Convenience sample. Response rates unclear Authors highlight that timing of survey and timeframes of questions meant the effects of physical distancing may not yet have emerged.
Hao *et al.,* 2020	China	A single Chinese hospital "designated for COVID-19" during lockdown 19th-21st Feb 2020. 76 case patients with mental illnesses on hospital list; 109 "healthy control" patients without mental illnesses through convenience sampling. All contacted via SMS.	Suicidal thoughts	There were significantly more patients with mental illness reporting suicidal ideation (n = 12; 15.7%) as compared to those without mental illness (n = 1; 0.9%) (p = 0.003)	It is not clear how control patients were sampled and from which population they were drawn. Measure to assess suicidal thoughts not described. Patients with mental illness would be expected to experience more suicidal thoughts compared to general population.
Kaparounaki *et al.,* 2020	Greece	1000 Greek university students sampled 4-9 ^th^ April	Suicidality RASS suicidality scale (Fountoulakis, 2012)	Respondents reported a 20.2% increase in "overall suicidality". Higher RASS scores than the general population in 2012.	Definition of ‘suicidality’ not given. Little methodological information. Letter to editor, probably not peer reviewed. The comparison population is derived from the literature and it is unclear if it includes all ages rather than people of the same age as the students (mean age 22 yrs, 68% females).
Killgore *et al.,* 2020a	USA	Nationally representative sample of 1,013 (18–35 years old; 567 females; 446 males) English speaking U.S. adults Participants were sampled from all 50 states, proportional to state population. Used the UCLA Loneliness Scale-3 Conducted in 3 ^rd^ week of lockdown (9–10 ^th^ April).	Suicidal thoughts question from PHQ-9	Lonely individuals (M=0.55±0.88) scored significantly higher than non-lonely (M=0.07±0.36) respondents on the PHQ- 9suicidal ideation item (F1,997=138.13,p<.0000 1,partialη2=.12) 34.9% of lonely respondents endorsed some level of suicidal ideation compared to 4.5% of non-lonely participants (OR: 10.97, 95% CI: 7.04-17.11;p<.00001).	No detailed discussion of sampling frame. Authors suggest impact of social distancing on loneliness and suicidal ideation is tangible at a population level Letter to editor, probably not peer reviewed.
Killgore *et al.,* 2020b	USA	As Killgore *et al.,* 2020b Completed Insomnia Severity Index ( Morin *et al.,* 2011) and adapted “COVID pandemic worry scale”(based on Wong *et al.,* 2007).	Suicidal thoughts question from PHQ-9	Cross-sectional analysis of the association between COVID worry and suicidal thoughts) and sleep mediation. Found weak correlation (r=0.11) between suicidal thoughts and COVID-worries; association attenuated / mediated via insomnia	As above.
Lee, 2020	Not specified	1237 recruited through Amazon Turk, a crowdsourcing website to hire remotely located “crowdworkers” to perform discrete on-demand tasks.. 675 male and 558 female responders (4 other); median age 35. 4.9% respondents reported having had COVID- 19. 25.4% ‘dysfunctional coronavirus anxiety’ Survey date 2 April 2020	Suicidal thoughts question from PHQ-9	A logistic regression, which controlled for sociodemographic effects of age, gender, education, and race, demonstrated that dysfunctional coronavirus anxiety was associated with suicidal ideation [odds ratio 1.24, 95% CI 1.13–1.37].	Convenience sample. Response rates unclear Participants received a payment of $0.50 Little information about the measure of ‘dysfunctional coronavirus anxiety’ but (see Lee *et al.,* 2020) suggestion it is associated with increased suicidal ideation.
Lee *et al.,* 2020	Not specified	398 Amazon Turk participants. 207 men and 191 women combined mean age of 35.91 (SD= 11.73) years Most were White (n= 286; 71.9%), , educated with a Bachelor's degree or higher (n= 253; 63.6%), Surveyed 11-13 March 2020	Suicidal thoughts measured by the question: “I wished I was already dead so I did not have to deal with the coronavirus.” Over last 2 weeks how many times on a five ponit scale	Assessed psychometric properties Coronavirus Anxiety Scale (CAS) and found scores were positively correlated with suicidal ideation (r= 0.71 p<0.001).	Convenience sample. Response rates unclear Participants received a payment of $0.50.
Plomecka *et al.,* 2020	Worldwide - 12 countries across 5 continents	On-line questionnaire promoted by social media posts, personal contacts and professional email lists, influences etc. Restricted to age 18+ 12817 usable responses from countries including USA (n=1864), Iran (1198), Pakistan (1173), Poland (1110), Italy (1096), Spain (972), Bosnia and Herzegovina (885), Turkey (539), Canada (538), Germany (534) Excluded people from African region; age <18)	Suicidal thoughts from Becks Depression Inventory-II	Factors known to be associated with suicidal thoughts (e.g. past trauma, age, low optimism) were (not surprisingly) associated with increased levels of suicidal thoughts as was worsening of of pre-existing psychiatric disorder during COVID-19 (OR: 4.66, 95% CI: [4.10, 5.29]. Ability to share concerns with family and friends as usual was associated with lower suicidal ideation (OR: 0.30, 95% CI: [0.26,0.36] Healthcare medical professionals had reduced risk of suicidal thoughts	Sampling frame and response rate unclear. Likely biased sample e.g. 72% respondents were female; 75% had a bachelors degree or above. Likely different samples in different countries. Pre-print, not peer reviewed
Sharif *et al.,* 2020	Global	Neurosurgeons approached from World Spinal Column Society. n=375 responses from 52 countries Anonymous on-line survey.	Suicidal thoughts SRQ20 questionnaire	5.1% (19/375) had suicidal thoughts	Response rate is unclear. No baseline pre-COVID data
Wu *et al.,* 2020a	China	Survivors of COVID-19, followed up median 22 days (IQR 20-30d) post hospital discharge. N=370	Suicidal thoughts question from PHQ-9	4 (1.1%) reported experiencing suicidal / self-harm thoughts over several days	Large survey of hospital admitted COVID-19 No pre-illness baseline measure. 1.1% prevalence of suicidal thoughts/ behaviour is surprisingly low Letter to editor, probably not peer reviewed.
Wu *el al.*, 2020b	China	4124 pregnant women during their third trimester from 25 public hospitals in 10 provinces Jan 1st-Feb 9th 2020 1285 assessed after January 20, 2020 when the coronavirus epidemic was publicly announced and 2839 were assessed before this time point.	Thoughts of self-harm in the last 7 days from the Edinburgh Postnatal Depression Scale (EPDS, Cox *et al.,* 1987)	A multi-centre study to identify mental health concerns in pregnancy The risk of self-harm thoughts was higher after 20 ^th^ January compared to before (aRR=2.85, 95% CI: 1.70, 8.85, P=0.005).	Pre-existing data collection system- Perinatal and Postpartum Depression Information Collection System. Element of before and after but not same women None directly related to SH but suggest risk communication for pregnant women who may be a heightened anxiety of vertical transmission and safety of their babies,
Zhao *et al.,* 2020	China	A survey from February 2nd-16 ^th^, 2020 of COVID-19 patients (n=106), 46 male, range 35-92 years at Tongji Hospital, Wuhan	Suicidal thoughts question from PHQ-9	24.5% (26/106) of COVID-19 patients had self-harming or suicidal thoughts, which were "significantly higher percentages than those of the general population."	Highlights the potential mental health support needs, and the risk faced by recovering COVID-19 patients No baseline data

**Table 3.** Summary of studies using modelling approaches to estimate the possible impact of the pandemic on suicide rates. The latest and previous versions of this table are available as extended data ( John & Schmidt, 2020).

Authors	Country / region model estimate relates to	Data used to inform estimate	Model prediction	Comment / Limitations
Bhatia, 2020	USA	Previous research modelling the association of unemployment with suicide in the USA indicating a 1% rise in unemployment was associated with a 1% rise in suicide. Assumes unemployment in the USA has risen from 3.8% to over 20%	7444 additional suicides in the following 2 months There were approximately 48,000 suicides in USA in 2018, so this equates to a predicted 15% rise in suicides in the USA.	No account for potential impacts of pandemic other than via unemployment rises Duration of unemployment rises uncertain Pre-print, not peer reviewed.
Kawohl & Nordt, 2020	World	Previous research modelling the association of unemployment with suicide in 63 countries (2000–2011). International Labour Organisations (ILO) Predicted job losses (March 2020) of between 5.3 to 24.7 million	Between 2135 and 9570 extra suicides per year worldwide. i.e. a 0.3% to 1.2% rise	No account for potential impacts of pandemic other than via unemployment rises Duration of unemployment rises uncertain Research letter, probably not peer reviewed.
McIntyre & Lee, 2020a	USA	The authors analysed theassociation of unemployment with suicide in the USA (1999–2018) and reported a 1% rise in unemployment was associated with a 1% rise in suicide. Three scenarios for changes in level of unemployment a) unchanged at 3.6%(2020), 3.7% (2021); b) rise to 5.8% (2020) and 9.3% (2021); c) rise to 24% (2020) and 18% (2021).	Scenario b) associated with a 3.3% rise in suicide in 2020–21 Scenario c) associated with an 8.4% rise in suicide in 2020–21.	Usefully models the potential impact of two different unemployment rate rises. No account for potential impacts of pandemic other than via unemployment rises Duration of unemployment rises uncertain
McIntyre & Lee, 2020b	Canada	The authors analysed the association of unemployment with suicide in Canada (2000–2018) and reported a 1% rise in unemployment was associated with a 1% rise in suicide. Three scenarios for changes in level of unemployment a) minimal change at 5.9%(2020), 6.0% (2021); b) rise to 8.3% (2020) and 8.1% (2021); c) rise to 16.6% (2020) and 14.9% (2021).	Scenario b) associated with a 5.5% rise in suicide in 2020–21 Scenario c) associated with a 27.7% rise in suicide in 2020–21.	Usefully models the potential impact of two different unemployment rate rises. No account for potential impacts of pandemic other than via unemployment rises Duration of unemployment rises uncertain
Moser *et al.,* 2020	Switzerland	Used published data on increased risk of suicide amongst a) prisoners in shared cells (3 fold increased risk) and b) prisoners in solitary confinement (27 fold increased risk) as indicators of risk of lock down on a) multi-person households and; b) single person households. Data on the annual number of suicides in Switzerland and the proportion of Swiss people living alone (16%) and in shared households (84%).	Estimate 1523 additional suicides. Based on an estimate the 1043 recorded suicides in Switzerland in 2017 this equates to a more than doubling in suicides deaths	The team modelled the impact of COVID- 19 pandemic on multiple outcomes as well as suicide. Prison confinement is probably not a good proxy for effects of lockdown. High suicide rates in prisoners are due to multiple factors e.g. age and gender profile; high levels of psychiatric morbidity rather than impacts of confinement. Other potential factors e.g. rises in unemployment not included in models Pre-print, not peer reviewed.

**Table 4.** Summary of studies assessing service utilisation. The latest and previous versions of this table are available as extended data ( John & Schmidt, 2020).

Authors	Country / region model estimate relates to	Data used	Outcome	Findings	Comment / Limitations
Pignon *et al.,* 2020	France	Emergency psychiatric consultations from three psychiatric emergency centres from first four weeks of lockdown (started March 17 ^th^ 2020) and corresponding weeks 2019	**Suicide** **attempts**	During the four first weeks of lockdown, 553 emergency psychiatric consultations were carried out, less than half (45.2%) of the corresponding weeks in 2019 (1224 consultations). Total suicide attempts decreased in 2020 to 42.6% of those in 2019.	Descriptive study.
Smalley *et al.,* 2020	USA	Attendees with suicidal ideation and alcohol issues across 20 diverse EDs in a large Midwest integrated healthcare system with >750,000 ED visits annually. All behavioural health (BH) visits were collected for 1-month (March 25 ^th^ to April 24, 2020) following “stay at home” orders (lockdown). Visits were identified if a BH ICD-10 code was used as a primary diagnosis or if behavioural complaints were listed. The same parameters were used to collect data for the same time period for 2019 to compare effects of COVID- 19 on ED visits.	Suicidal thoughts ICD coded by hospital staff	Between 2019 and 2020, there was 44.4% decrease in overall ED visits and 28.0% decrease in BH visits. Attendances with suicidal thoughts encounters decreased by 60.6% 2020 vs. 2019. As a percentage of all ED attendances , Suicidal thoughts attendances decreased from 2.03% to 1.44% from 2019 to 2020. SI encounters fell from 33.28% in 2019 to 18.21% in 2020 (p < .001) when examining percentage of overall BH encounters within the system.	Alternative avenues for help-seeking not included. But highlights importance of improving access for vulnerable populations during a pandemic. Letter to editor, probably not peer reviewed
Titov *et al.,* 2020	Australia	Callers / website visits to "Mindspot" - national digital MH service in Australia Compared caller volume and characteristics 1-28 Sept 2019 (n=1650) vs. 19 March - 15 April 2020 (n=1668)	Suicidal thoughts question from PHQ-9 Suicide attempts/ self-harm	No change in prevalence of a) suicidal thoughts (30.6% pre vs. 27.5% during; p=0.08) or b) suicidal intentions or plans (3.7% pre- and 2.9% post p=0.27)	Before and after study Clinical / helpline sample - not population based Possible seasonal differences- September contacts vs. March-April Evidence of increased contact volume to a digital service.

### Study populations

Two articles shared study populations ( Killgore *et al*., 2020a; Killgore *et al*., 2020b). Excluding duplicate populations and modelling studies, the total number of unique participants was 33, 345. Most studies included both male and female participants except ( Wu *et al*., 2020b) which was conducted in a population of pregnant women in their third trimester.

### Outcomes

Two of the eight case series focused on suicide attempts and six on suicide deaths. Of the 12 independent cross-sectional surveys ten assessed suicidal thoughts of which two also assessed suicide attempts ( Ammerman *et al*., 2020; Bryan *et al*., 2020), one thoughts of self-harm ( Wu *et al*., 2020b) using a single item from the Edinburgh Postnatal Depression Scale (EPDS), one suicidality ( Kaparounaki *et al*., 2020) using the Risk Assessment Suicidality Scale (RASS). A range of validated questionnairres were used to assess suicidal thoughts. Four used the question 9 single item from PHQ-9 ‘Have you had thoughts that you would be better off dead or of hurting yourself in some way’ with four levels of response ranging from ‘not at all’ to ‘nearly every day’ over the last 2 weeks. One each used: the Beck Depression Inventory-II (with one item where the participant choses one statement from among a group of four statements that best describes how they have been feeling during the past few days, ranging from ‘I don’t have thoughts of killing myself’ to ‘I would kill myself if I had the chance’); the WHO Self Reporting Questionnaire (with one item of 20 asking ‘Has the thought of ending your life been on your mind?’, response *yes/no* in the last 30 days); one used the question how many times over the last two weeks have you thought ‘I wished I was already dead so I did not have to deal with the coronavirus’ on a five point scale; and in two little detail was given regarding this outcome assessment.

Two studies used the Self-injurous Thoughts and Behaviours Interview (SITBI) to assess for presence ( *yes*/ *no*) of active suicidal thoughts (i.e., ‘Have you had thoughts of killing yourself?’) in the past month ( Ammerman *et al*., 2020) and the other in the past month, year or over a year ago ( Bryan *et al*., 2020). They also included the item for suicide attempts. Ammerman *et al*. (2020) used one adapted item from the SITBI ‘In the past month, have you attempted to kill yourself?’ ( *yes/no*) and Bryan *et al*. (2020) ‘Have you ever made an actual attempt to kill yourself in which you had at least some intent to die?’ ( *yes/no*) within the past month, year or more than a year ago.

### Summary of studies’ findings: Case series

We identified eight case series reports of suicide attempts and suicide deaths ( Table 1). Five of these used news reports as their data source ( Bhuiyan *et al*., 2020; Dsouza *et al*., 2020; Griffiths & Mamun, 2020; Mamun & Ullah, 2020; Thakur & Jain, 2020). Many reasons for COVID-19 related suicide or suicide attempts were suggested and usually this information was derived from a journalist’s report of the death. Contributory factors reported included fear of contracting the disease or of passing it on to others, reactive psychoses, financial or economic issues, loneliness and isolation due to quarantine, stress among health professionals, the uncertainty around when the pandemic would end, an inability for migrants to return home, frustration and the stigma of a (possibly perceived) positive result, which resulted in harassment or victimisation by others in the community. The largest case series ( Dsouza *et al*., 2020) (n=72 suicide deaths) reported that the most commonly occurring antecedents to suicide were fear of infection (n=21) and financial crisis (n=19). One case series ( Griffiths & Mamun, 2020), based on news reports, included suicide pacts by 6 couples (including one murder suicide and one double suicide attempt) from Bangladesh, India, Malaysia and the USA.

### Summary of studies’ findings: Cross-sectional surveys

There were 13 articles describing cross-sectional surveys, reporting 12 independent studies ( Table 2). Seven articles (6 independent studies) reported cross-sectional surveys in the general population. One study ( Killgore *et al*., 2020a; Killgore *et al*., 2020b) was a nationally representative sample of English speaking participants aged 18-35 years from 50 US states; however, no details were given regarding how the participants were sampled. Bryan *et al*. (2020) used a panel quota sampling methodology and weighted their sample to match the USA general population by age, sex and ethnicity. Three studies used convenience sampling through Amazon Mechanical Turk crowdsourcing ( Ammerman *et al*., 2020; Lee, 2020; Lee *et al*., 2020), which pays survey responders a small fee for participation and one ( Plomecka *et al*., 2020) used online recruitment.

Participants were COVID-19 patients in three studies ( Hao *et al*., 2020; Wu *et al*., 2020a; Zhao *et al*., 2020) and surveys were targeted at specific poulations in a further three: pregnant women ( Wu *et al*., 2020b)), neurosurgeons ( Sharif *et al*., 2020) and university students ( Kaparounaki *et al*., 2020). The study by Wu *et al*. (2020b) was the only survey to report pre-pandemic/pre-illness data for comparison, although Killgore *et al*. (2020a) compared their findings to previous work ( Morahan-Martin & Schumacher, 2003) and a number of studies compared their findings to estimates that were reported from earlier published studies.

Higher levels of suicidal/self-harm thoughts were reported in individuals with: anxiety relating to COVID-19 ( Lee, 2020); worry relating to COVID-19 mediated by insomnia ( Killgore *et al*., 2020b); with loneliness ( Killgore *et al*., 2020a); worsening of pre-existing mental illness during COVID-19 ( Hao *et al*., 2020; Plomecka *et al*., 2020); and in students ( Kaparounaki *et al*., 2020); people recovering from COVID-19 infection ( Hao *et al*., 2020); as well as women who were in their third trimester of pregnancy during the pandemic, compared with measures taken amongst women at the same stage of pregnancy before the pandemic ( Wu *et al*., 2020b). As these are cross-sectional studies the direction of association is not possible to determine and only one study used pre-pandemic measures recorded in the same population in a similar way ( Wu *et al*., 2020b).

One study carried out in the USA exploited the natural experiment provided by states imposing physical distancing measures on different dates ( Bryan *et al*., 2020). This study found no evidence of an increased risk of suicidal thoughts or attempts amongst those living in states with either stay-at-home orders or restrictions on large gatherings in place compared with states without these measures.

### Summary of studies’ findings: Modelling studies

We identified five studies ( Table 3) that have used modelling approaches to forecast the potential impact of the pandemic on future suicide rates ( Bhatia, 2020; Kawohl & Nordt, 2020; McIntyre & Lee, 2020a; McIntyre & Lee, 2020b; Moser *et al*., 2020). Each was based on different assumptions, but models largely focused on the well-characterised impact on suicide rates of rises in unemployment ( Chang *et al*., 2013; Stuckler *et al*., 2009). Unemployment rates are predicted to rise as a result of a post-pandemic recession, due to measures to control the spread of the virus on the wider economy and loss of work as many businesses have been forced to shut down.

Only one study modelled the effects of physical distancing measures on suicide rates ( Moser *et al*., 2020); it did this by using suicide rates in prisoners in group or single cells as a model for lock-down in a group or in isolation. The prison population is exposed to multiple other risk factors for suicide (e.g. increased prevalence of mental illness, substance misuse and low socioeconomic position) ( Humber *et al*., 2011; Rivlin *et al*., 2010), and this, coupled with the distinct differences between prison incarceration and the adoption of home quarantine procedures during the pandemic, this model is likely to over-estimate the potential impact of physical distancing measures on suicide.

The models suggest between a 1% rise (globally) ( Kawohl & Nordt, 2020) and a 145% rise (in Switzerland) ( Moser *et al*., 2020) in suicide deaths.

### Summary of studies’ findings: Service utilisation studies

We identified three service utilisation studies ( Pignon *et al*., 2020; Smalley *et al*., 2020; Titov *et al*., 2020) ( Table 4). Smalley *et al*. (2020) reported a fall in ED visits for suicidal thoughts in Midwest USA, as well as a fall in the proportion of total visits that were for suicidal thoughts. In contrast Titov *et al*. (2020) found evidence of increased contact volume to a national digital mental health service in Australia. However, amongst contacts, while there was evidence of increased anxiety and levels of concerns about COVID-19, which increased with age, there was no evidence that the percentage of contacts with suicidal thoughts/plans increased. Pignon *et al.,* 2020 reported that emergency psychiatric consultations for suicide attempts more than halved in a region of Paris in the first month of lockdown, compared to the same period in 2019.

## Discussion

In total, 28 independent studies (29 articles) were included in this review covering a total of 33,345 studied individuals from around the world with a mix of low, middle and high income countries. Almost half of the articles were pre-prints published before peer review, or research letters that may not have been peer-reviewed. The majority of studies were case series or cross sectional surveys, almost all based on non-representative convenience samples. Only one study reported on the change in incidence of suicide or suicidal behaviour before versus after the onset of the pandemic ( Pignon *et al*., 2020); this analysis was based on emergency psychiatric consultations for suicide attempt – and reported a decline, although levels of consultation could have been influenced by fears about using services or ideas of not burdening the health service rather than changes in incidence. A further study from China reported heightened levels of self-harm thoughts in pregnant women surveyed in the period after the onset of the pandemic, compared with levels reported amongst women surveyed at the same stage of pregnancy just before the pandemic ( Wu *et al*., 2020b). No studies reported potentially harmful effects of lockdown/physical distancing measures in relation to our outcomes, although one study comparing the prevalence of suicidal thoughts and attempts in people living in USA states with varying timing and strigency of state-specific lockdowns found no evidence for such an ecological association ( Bryan *et al*., 2020). Modelling studies that aimed to predict the impact of the pandemic on national or global suicide rates produced widely differing estimates of the likely impact and most focused on predictions based on previous studies of the impact of changes in unemployment levels on suicide. Three studies investgated service use patterns – one found a decline in ED visits for suicidal thoughts, one a decline in psychiatric emergency consultation for suicide attempt and the other reported an increase in contacts to a mental health digital platform but no changes in contacts for suicidal thoughts.

We identified eight case series reports of suicide attempts and suicide deaths, five based on news stories in India, Bangladesh and Pakistan. Given the relatively low quality of case series in the hierarchy of evidence, often reflecting small numbers and selection bias, but more importantly the lack of comparator data, drawing any reliable inferences from these studies is challenging. Furthermore, news reports report a non-representative sample of suicide deaths and often derive their information from bystanders and witnesses who are unlikely to know the full circumstances of the death ( Khan *et al*., 2009). Nevertheless, these studies highlight circumstances surrounding apparently COVID-19-related suicides and flag the potential importance of factors such as economic difficulties, fear of the disease, and social isolation. Indeed in parts of the world without reliable suicide incidence data they may be the only source of information ( Khan & Hyder, 2006).

The 12 cross-sectional studies investigated a range of issues. Findings indicated worries about COVID-19 and recent COVID-19 infection were associated with suicidal thoughts ( Hao *et al*., 2020; Killgore *et al*., 2020a; Killgore *et al*., 2020b; Lee, 2020; Lee *et al*., 2020; Zhao *et al*., 2020) and, amongst pregnant women surveyed during the pandemic, thoughts of self-harm were higher than amongst those surveyed pre-pandemic. The one study comparing suicidal thoughts and behaviours amongst people living in areas with versus without physical distancing measures found no adverse association ( Bryan *et al*., 2020). Surprisingly survey by Ammerman *et al*. (2020) from the USA indicated that social distancing was associated with reduced instances of suicidal thoughts early in the period of lockdown. Only one survey suggested it was nationally representative but lacked sampling details ( Killgore *et al*., 2020a). Non-probability sampling lacks a sound theoretical basis for statistical inference ( Neyman, 1934). Consequently, basic descriptive analyses and explorations of potential associations are appropriate but measures of uncertainty (i.e., confidence intervals around estimates of prevalence) are generally not valid. One study ( Bryan *et al*., 2020) used panel quota sampling, but these sorts of adjustments for age, sex and ethnicity may miss other elements of bias and cannot account for groups not included at all, particularly if the response rate is unknown ( Pierce *et al*., 2020). Four studies used convenience sampling which tend to attract volunteers who have access to the internet, are already engaged in research and have an interest in the topic. Hence responses may be unrepresentative of the general population, and associations observed among these healthy volunteers may not reflect associations that would be observed in others. Similarly, when assessing suicidal thoughts and behaviours, those in most distress or with co-existing mental illness, as well as older people, are less likely to participate in these sorts of surveys. There is no way to assess non-response bias in a convenience sample as might be possible in a probability-sampled survey ( Pierce *et al*., 2020).

There was a large range in modelling estimates of the effect of the pandemic on suicide rates, varying between a 1% and a 145% rise. These differences between model estimates were partly due to differences in modelling assumptions, which are associated with considerable uncertainty. Given the methodological limitations, the uncertainty of assumptions about how the economies of individual countries will be affected, as well as international differences in financial supports given to businesses and people out of work, these predictive exercises can at best only provide a guide as to where action should be directed.

### Strengths and Limitations

To date, there is little literature exploring COVID-19 and suicide deaths, suicidal behaviours, self-harm and suicidal thoughts and most of the published evidence that we identified had important limitations. Importantly, much of the literature is not yet peer reviewed so the quality of reported studies may change. There were eight research letters, five pre-prints and for many others very short timeframes between submission and acceptance. All included studies were observational in design and prone to multiple sources of bias (eg, recall bias, selection bias, confounding). No conclusions can be drawn regarding causality and temporality from cross sectional studies. However, such study designs may be appropriate in current circumstances where timeliness of studies to inform policy and practice are important. However many were carried out too quickly and too early (one to two weeks post lockdown) in the outbreak to make meaningful contributions to the evidence base. The lack of baseline data in the majority of surveys included in the review and adjustments made to standardised measures to assess suicidal behaviours as well as the range of measures and timing asked made assessment of findings problematic.

We did not include Google Trends studies ( Jacobson *et al*., 2020; Knipe *et al*., 2020; Rana, 2020; Sinyor *et al*., 2020) since search data constitute a proxy measure but findings are largely mixed. We also excluded grey literature ( Fancourt & Steptoe, 2020).

### Implications

A marked improvement in the quality of design, methods, and reporting in future studies is needed. There is thus far no clear evidence of an increase in suicidal behaviour or self-harm associated with the pandemic nor with the measures taken to curb the spread of COVID-19. The current iteration of out living review highlights the methodological issues of early evidence from around the world that assesses the impact of the COVID-19 pandemic on suicide deaths, suicidal behaviours, self-harm and suicidal thoughts, or that assesses the effectiveness of strategies to reduce the risk of suicide deaths, suicidal behaviours, self-harm and suicidal thoughts, resulting from the COVID-19 pandemic. However, suicide data are challenging to collect in real time and the economic effects are evolving. Our living review will provide a regular synthesis of the most up-to-date research evidence to guide public health and clinical policy to mitigate the impact of COVID-19 on suicide.

## Data availability

### Underlying data

Harvard Dataverse: Full review data for: "The impact of the COVID-19 pandemic on self-harm and suicidal behaviour: update of living systematic review". https://doi.org/10.7910/DVN/7WZXZK ( John & Schmidt, 2020)

This project contains the following underlying data:

- Screening_snapshot.csv (Screening progress for literature published before June 7th)

### Extended data

Harvard Dataverse: Full review data for: "The impact of the COVID-19 pandemic on self-harm and suicidal behaviour: update of living systematic review". https://doi.org/10.7910/DVN/7WZXZK ( John & Schmidt, 2020)

This project contains the following extended data:

- LSR update tables and figures.docx (Tables and figures from this publication)
- PRISMA.doc

Data regarding the Protocol are available via our Harvard Dataverse repository for the protocol

Harvard Dataverse: Underlying data for: The impact of the Covid-19 pandemic on suicidal behaviour: a living systematic review protocol. https://doi.org/10.7910/DVN/9JYHLS ( John *et al.*, 2020b)

That project contains the following extended data:

- Search.docx (additional information about the searches, including full search strategies)
- Data extraction sheet/ study report
- Figure 1
- Prisma.pdf (the PRISMA-P statement)
- Prospero registration

### Reporting guidelines

Harvard Dataverse: PRISMA checklist for ‘The impact of the COVID-19 pandemic on self-harm and suicidal behaviour: a living systematic review’ https://doi.org/10.7910/DVN/7WZXZK ( John & Schmidt, 2020)

Data are available under the terms of the Creative Commons Attribution 4.0 International license (CC-BY 4.0).

## Software availability

The development version of the software for automated searching is available from Github: https://github.com/mcguinlu/COVID_suicide_living.

Archived source code at time of publication: http://doi.org/10.5281/zenodo.3871366 ( McGuinness & Schmidt, 2020)

License: MIT

## References

[ref-1] Akl EA Meerpohl JJ Elliott J: Living systematic reviews: 4. Living guideline recommendations. *J Clin Epidemiol.* 2017;91:47–53. 10.1016/j.jclinepi.2017.08.009 28911999

[ref-2] Ammerman BA Burke TA Jacobucci R: Preliminary Investigation of the Association Between COVID-19 and Suicidal Thoughts and Behaviors in the US. *PsyArXiv.* 2020 10.31234/osf.io/68djp 33360222

[ref-3] Bhatia R: Predictions of Covid-19 Related Unemployment On Suicide and Excess Mortality in the United States. *medRxiv.* 2020 10.1101/2020.05.02.20089086

[ref-4] Bhuiyan AI Sakib N Pakpour AH: COVID-19-related suicides in Bangladesh due to lockdown and economic factors: case study evidence from media reports. *Int J Ment Health Ad.* 2020;1–6. 10.1007/s11469-020-00307-y 32427168 PMC7228428

[ref-5] Bryan C Bryan AO Baker JC: Associations among state-level physical distancing measures and suicidal thoughts and behaviors among US adults during the early COVID-19 pandemic. *PsyArXiv.* 2020 10.1111/sltb.12653 PMC7362130 32589801

[ref-80] Buschmann C Tsokos M: Corona-associated suicide - Observations made in the autopsy room. *Leg Med (Tokyo).* 2020;46:101723. 10.1016/j.legalmed.2020.101723 32526673 PMC7267788

[ref-6] Chang SS Stuckler D Yip P: Impact of 2008 global economic crisis on suicide: time trend study in 54 countries. *BMJ.* 2013;347:f5239. 10.1136/bmj.f5239 24046155 PMC3776046

[ref-7] Cheung Y Chau PH Yip PS: A revisit on older adults suicides and Severe Acute Respiratory Syndrome (SARS) epidemic in Hong Kong. *Int J Geriatr Psychiatry.* 2008;23(12):1231–1238. 10.1002/gps.2056 18500689

[ref-81] Cox J Holden J Sagovsky R: Detection of postnatal depression: Development of the 10-item Edinburgh Postnatal Depression Scale. *Br J Psychiatry.* 1987;150:782–786. 10.1192/bjp.150.6.782 3651732

[ref-8] Deeks JJ Higgins JP Altman DG: Chapter 10: Analysing data and undertaking meta-analyses. *Cochrane handbook for systematic reviews of interventions.*2nd ed.: John Wiley & Sons. 2019 Reference Source

[ref-9] Dsouza DD Quadros S Hyderabadwala ZJ: Aggregated COVID-19 suicide incidences in India: Fear of COVID-19 infection is the prominent causative factor. *Psychiatry Res.* 2020;290:113145. 10.1016/j.psychres.2020.113145 32544650 PMC7832713

[ref-10] Elliott JH Synnot A Turner T: Living systematic review: 1. Introduction—the why what, when, and how. *J Clin Epidemiol.* 2017;91:23–30. 10.1016/j.jclinepi.2017.08.010 28912002

[ref-11] Elliott JH Turner T Clavisi O: Living systematic reviews: an emerging opportunity to narrow the evidence-practice gap. *PLoS Med.* 2014;11(2):e1001603. 10.1371/journal.pmed.1001603 24558353 PMC3928029

[ref-12] Fancourt D Steptoe A: COVID-19 social study. *Nuffield Foundation.* 2020 Reference Source

[ref-13] Finlay I Gilmore I: Covid-19 and alcohol—a dangerous cocktail. *BMJ.* 2020;369:m1987. 10.1136/bmj.m1987 32434792

[ref-14] Griffiths MD Mamun MA: COVID-19 suicidal behavior among couples and suicide pacts: Case study evidence from press reports. *Psychiatry Res.* 2020;289:113105. 10.1016/j.psychres.2020.113105 PMC7229970 33242807

[ref-15] Gunnell D Appleby L Arensman E: Suicide risk and prevention during the COVID-19 pandemic. *Lancet Psychiatry.* 2020;7(6):468–471. 10.1016/S2215-0366(20)30171-1 32330430 PMC7173821

[ref-16] Hao F Tan W Jiang L: Do psychiatric patients experience more psychiatric symptoms during COVID-19 pandemic and lockdown? A case-control study with service and research implications for immunopsychiatry. *Brain Behav Immun.* 2020;87:100–106. 10.1016/j.bbi.2020.04.069 32353518 PMC7184991

[ref-17] Holmes EA O'Connor RC Perry VH: Multidisciplinary research priorities for the COVID-19 pandemic: a call for action for mental health science. *Lancet Psychiatry.* 2020;7(6):547–560. 10.1016/S2215-0366(20)30168-1 32304649 PMC7159850

[ref-18] Humber N Piper M Appleby L: Characteristics of and trends in subgroups of prisoner suicides in England and Wales. *Psychol Med.* 2011;41(11): 2275–2285. 10.1017/S0033291711000705 21557891

[ref-19] Jacobson NC Lekkas D Price G: Flattening the Mental Health Curve: COVID-19 Stay-at-Home Orders Are Associated With Alterations in Mental Health Search Behavior in the United States. *JMIR Ment Health.* 2020;7(6):e19347. 10.2196/19347 32459186 PMC7265799

[ref-20] John A Eyles E Mcguinness LA: The impact of the COVID-19 pandemic on self-harm and suicidal behaviour: protocol for a living systematic review [version 1; peer review: 1 approved, 1 approved with reservations] *F1000Research.* 2020a;9:644 10.12688/f1000research.24274.1 PMC7871358 33604025

[ref-21] John A Eyles EC McGuinness LA: Underlying data for: The impact of the Covid-19 pandemic on suicidal behaviour: a living systematic review protocol [Data set].Harvard Dataverse. 2020b 10.7910/DVN/9JYHLS

[ref-22] John J Schmidt L: "Full review data for: "The impact of the COVID-19 pandemic on self-harm and suicidal behaviour: update of living systematic review"". *Harvard Dataverse*, V3, 2020 10.7910/DVN/7WZXZK PMC7871358 33604025

[ref-23] Johnson NP Mueller J: Updating the accounts: global mortality of the 1918-1920" Spanish" influenza pandemic. *Bull Hist Med.* 2002;76(1):105–115. 10.1353/bhm.2002.0022 11875246

[ref-24] Kaparounaki CK Patsali ME Mousa DPV: University students’ mental health amidst the COVID-19 quarantine in Greece. *Psychiatry Res.* 2020;290:113111. 10.1016/j.psychres.2020.113111 32450416 PMC7236729

[ref-25] Kawohl W Nordt C: COVID-19, unemployment, and suicide. *Lancet Psychiatry.* 2020;7(5):389–390. 10.1016/S2215-0366(20)30141-3 32353269 PMC7185950

[ref-26] Khan MM Ahmed A Khan SR: Female suicide rates in Ghizer, Pakistan. *Suicide Life Threat Behav.* 2009;39(2):227–230. 10.1521/suli.2009.39.2.227 19527163

[ref-27] Khan MM Hyder AA: Suicides in the developing world: Case study from Pakistan. *Suicide Life Threat Behav.* 2006;36(1):76–81. 10.1521/suli.2006.36.1.76 16676628

[ref-28] Killgore WD Cloonan SA Taylor EC: Loneliness: A signature mental health concern in the era of COVID-19. *Psychiatry Res.* 2020a;290:113117. 10.1016/j.psychres.2020.113117 32480121 PMC7255345

[ref-29] Killgore WD Cloonan SA Taylor EC: Suicidal ideation during the COVID-19 pandemic: the role of insomnia. *Psychiatry Res.* 2020b;290:113134. 10.1016/j.psychres.2020.113134 32505030 PMC7255187

[ref-30] Kisely S Warren N McMahon L: Occurrence, prevention, and management of the psychological effects of emerging virus outbreaks on healthcare workers: rapid review and meta-analysis. *BMJ.* 2020;369. 10.1136/bmj.m1642 32371466 PMC7199468

[ref-31] Knipe D Evans H Marchant A: Mapping population mental health concerns related to COVID-19 and the consequences of physical distancing: a Google trends analysis [version 2; peer review: 2 approved] *Wellcome Open Res.* 2020;5:82. 10.12688/wellcomeopenres.15870.2 32671230 PMC7331103

[ref-32] Lee SA: Coronavirus Anxiety Scale: A brief mental health screener for COVID-19 related anxiety. *Death Stud.* 2020;44(7):393–401. 10.1080/07481187.2020.1748481 32299304

[ref-33] Lee SA Mathis AA Jobe MC: Clinically significant fear and anxiety of COVID-19: A psychometric examination of the Coronavirus Anxiety Scale. *Psychiatry Res.* 2020;290:113112. 10.1016/j.psychres.2020.113112 32460185 PMC7237368

[ref-34] Mahase E: Covid-19: EU states report 60% rise in emergency calls about domestic violence. *BMJ.* 2020;369:m1872. 10.1136/bmj.m1872 32393463

[ref-35] Mamun MA Ullah I: COVID-19 suicides in Pakistan, dying off not COVID-19 fear but poverty?–The forthcoming economic challenges for a developing country. *Brain Behav Immun.* 2020;87:163–166. 10.1016/j.bbi.2020.05.028 32407859 PMC7212955

[ref-36] McGuinness LA Schmidt L: mcguinlu/COVID_suicide_living: Initial Release (v1.0.0) [Computer software]. *Zenodo.* 2020 10.5281/ZENODO.3871366

[ref-37] Mcintyre RS Lee Y: Preventing suicide in the context of the COVID-19 pandemic. *World Psychiatry.* 2020a;19(2):250–251. 10.1002/wps.20767 32394579 PMC7214950

[ref-38] Mcintyre RS Lee Y: Projected increases in suicide in Canada as a consequence of COVID-19. *Psychiatry Res.* 2020b;290:113104. 10.1016/j.psychres.2020.113104 32460184 PMC7236718

[ref-39] Moher D Shamseer L Clarke M: Preferred reporting items for systematic review and meta-analysis protocols (PRISMA-P) 2015 statement. *Syst Rev.* 2015;4(1):1. 10.1186/2046-4053-4-1 25554246 PMC4320440

[ref-40] Morahan-martin J Schumacher P: Loneliness and social uses of the Internet. *Comput Hum Behav.* 2003;19(6):659–671. 10.1016/S0747-5632(03)00040-2

[ref-41] Morgan R Sterne JA Higgins JP: A new instrument to assess Risk of Bias in Non-randomised Studies of Exposures (ROBINS-E): Application to studies of environmental exposure *Global Evidence Summit.* Cape Town.2017 Reference Source

[ref-82] Morin CM Belleville G Bélanger L: The Insomnia Severity Index: psychometric indicators to detect insomnia cases and evaluate treatment response. *Sleep.* 2011;34(5):601–608. 10.1093/sleep/34.5.601 21532953 PMC3079939

[ref-42] Moser DA Glaus J Frangou S: Years of life lost due to the psychosocial consequences of COVID19 mitigation strategies based on Swiss data. *Eur Psychiatry.* 2020;63(1):e58. 10.1192/j.eurpsy.2020.56 32466820 PMC7303469

[ref-43] Neyman J: On the two different aspects of the representative method: the method of stratified sampling and the method of purposive selection. *J R Stat Soc B.* 1934;97(4):558–625. 10.2307/2342192

[ref-44] Pierce M Mcmanus S Jessop C: Says who? The significance of sampling in mental health surveys during COVID-19. *Lancet Psychiatry.* 2020;7(7):567–568. 10.1016/S2215-0366(20)30237-6 32502467 PMC7266586

[ref-45] Pignon B Gourevitch R Tebeka S: Dramatic reduction of psychiatric emergency consultations during lockdown linked to COVID-19 in Paris and suburbs. *Psychiatry Clin Neurosci.* 2020. 10.1111/pcn.13104 32609417 PMC7361336

[ref-46] Plomecka MB Gobbi S Neckels R: Mental Health Impact of COVID-19: A global study of risk and resilience factors. *medRxiv.* 2020 10.1101/2020.05.05.20092023

[ref-47] Rana U: Elderly Suicides in India: An Emerging Concern during COVID-19 Pandemic. *Int Psychogeriatr.* 2020;1–2. 10.1017/S1041610220001052 32487275 PMC7322164

[ref-48] Reger MA Stanley IH Joiner TE: Suicide Mortality and Coronavirus Disease 2019—A Perfect Storm? *JAMA Psychiatry.* 2020. 10.1001/jamapsychiatry.2020.1060 32275300

[ref-49] Rivlin A Hawton K Marzano L: Psychiatric disorders in male prisoners who made near-lethal suicide attempts: case–control study. *Br J Psychiatry.* 2010;197(4):313–319. 10.1192/bjp.bp.110.077883 20884955

[ref-83] Sahoo S Bharadwaj S Parveen S: Self-harm and COVID-19 Pandemic: An emerging concern–A report of 2 cases from India. *Asian J Psychiatr.* 2020;51:102104. 10.1016/j.ajp.2020.102104 32325391 PMC7161515

[ref-50] Sharif S Amin F Hafiz M: COVID 19-Depression and Neurosurgeons. *World Neurosurg.* 2020;140:e401–e410. 10.1016/j.wneu.2020.06.007 32512242 PMC7274976

[ref-51] Sinyor M Spittal MJ Niederkrotenthaler T: Changes in Suicide and Resilience-related Google Searches during the Early Stages of the COVID-19 Pandemic. *Can J Psychiatry.* 2020; 706743720933426. 10.1177/0706743720933426 32524848 PMC7502879

[ref-52] Smalley CM Malone DA Meldon SW: The impact of COVID-19 on suicidal ideation and alcohol presentations to emergency departments in a large healthcare system. *Am J Emerg Med.* 2020; S0735-6757(20)30457-5. 10.1016/j.ajem.2020.05.093 32505472 PMC7263212

[ref-53] Sterne JA HernÁn MA Reeves BC: ROBINS-I: a tool for assessing risk of bias in non-randomised studies of interventions. *BMJ.* 2016;355:i4919. 10.1136/bmj.i4919 27733354 PMC5062054

[ref-54] Stuckler D Basu S Suhrcke M: The public health effect of economic crises and alternative policy responses in Europe: an empirical analysis. *Lancet.* 2009;374(9686):315–323. 10.1016/S0140-6736(09)61124-7 19589588

[ref-55] Thakur V Jain A: COVID 2019-suicides: A global psychological pandemic. *Brain Behav Immun.* 2020;88:952–953. 10.1016/j.bbi.2020.04.062 32335196 PMC7177120

[ref-56] Titov N Staples L Kayrouz R: Rapid report: Early demand, profiles and concerns of mental health users during the coronavirus (COVID-19) pandemic. *Internet Interv.* 2020;21:100327. 10.1016/j.invent.2020.100327 32537424 PMC7262525

[ref-57] Turecki G Brent DA Gunnell D: Suicide and suicide risk. *Nat Rev Dis Primers.* 2019;5:73 10.1038/s41572-019-0130-z 31649257

[ref-84] Valdés-Florido MJ López-Díaz A Palermo-Zeballos FJ: Reactive psychoses in the context of the COVID-19 pandemic: clinical perspectives from a case series. * Rev Psiquiatr Salud Ment.* 2020;13(2):90–94. 10.1016/j.rpsm.2020.04.009 38620329 PMC7183984

[ref-58] Wasserman IM: The impact of epidemic,war prohibition and media on suicide: United States, 1910– 1920. *Suicide Life Threat Behav.* 1992;22(2):240–254. 1626335

[ref-85] Wong TW Gao Y Tam WWS: Anxiety among university students during the SARS epidemic in Hong Kong. *Stress Health.* 2007;23(1):31–35. 10.1002/smi.1116

[ref-59] Worldometer: Covid-19 Coronavirus Pandemic [Online].2020 [Accessed 07/06/2020]. Reference Source

[ref-60] Wu C Hu X Song J: Mental health status and related influencing factors of COVID-19 survivors in Wuhan, China. *Clin Transl Med.* 2020a;10(2):e52. 10.1002/ctm2.52 32508037 PMC7300592

[ref-61] Wu Y Zhang C Liu H: Perinatal depressive and anxiety symptoms of pregnant women along with COVID-19 outbreak in China. *Am J Obstet Gynecol.* 2020b;223(2):240.e1–240.e9. 10.1016/j.ajog.2020.05.009 32437665 PMC7211756

[ref-62] Zhao Q Hu C Feng R: Investigation of the mental health of patients with novel coronavirus pneumonia. *Chinese Journal of Neurology.* 2020.

