# The impact of the COVID-19 pandemic on self-harm and suicidal behaviour: update of living systematic review

**DOI:** 10.12688/f1000research.25522.2

**Published:** 2021-06-17

**Authors:** Ann John, Emily Eyles, Roger T. Webb, Chukwudi Okolie, Lena Schmidt, Ella Arensman, Keith Hawton, Rory C. O'Connor, Nav Kapur, Paul Moran, Siobhan O'Neill, Luke A. McGuiness, Babatunde K. Olorisade, Dana Dekel, Catherine Macleod-Hall, Hung-Yuan Cheng, Julian P.T. Higgins, David Gunnell

**Affiliations:** 1 Population Psychiatry, Suicide and Informatics, Swansea University, Swansea, UK; 2 Public Health Wales NHS Trust, Swansea, UK; 3 National Institute for Health Research Applied Research Collaboration West (NIHR ARC West) at University Hospitals Bristol NHS Foundation Trust, Bristol, UK; 4 Population Health Sciences, Bristol Medical School, University of Bristol, Bristol, UK; 5 Division of Psychology and Mental Health, University of Manchester, Manchester, UK; 6 NIHR Greater Manchester Patient Safety Translational Research Centre, Manchester, UK; 7 School of Public Health and National Suicide Research Foundation, University College Cork, Cork, Ireland; 8 University Department of Psychiatry, Centre for Suicide Research, University of Oxford, Oxford, UK; 9 Oxford Health NHS Foundation Trust, Oxford, UK; 10 Institute of Health & Wellbeing, University of Glasgow, Glasgow, UK; 11 Greater Manchester Mental Health NHS Foundation Trust, Manchester, UK; 12 National Institute for Health Research Biomedical Research Centre at the University Hospitals Bristol NHS Foundation Trust and the University of Bristol, Bristol, UK; 13 School of Psychology, University of Ulster, Coleraine, UK

**Keywords:** COVID-19, Living systematic review, Suicide; Attempted suicide, Self-harm, Suicidal thoughts

## Abstract

**Background: **The COVID-19 pandemic has caused considerable morbidity, mortality and disruption to people’s lives around the world. There are concerns that rates of suicide and suicidal behaviour may rise during and in its aftermath. Our living systematic review synthesises findings from emerging literature on incidence and prevalence of suicidal behaviour as well as suicide prevention efforts in relation to COVID-19, with this iteration synthesising relevant evidence up to 19 ^th^ October 2020.

**Method: ** Automated daily searches feed into a web-based database with screening and data extraction functionalities. Eligibility criteria include incidence/prevalence of suicidal behaviour, exposure-outcome relationships and effects of interventions in relation to the COVID-19 pandemic. Outcomes of interest are suicide, self-harm or attempted suicide and suicidal thoughts. No restrictions are placed on language or study type, except for single-person case reports. We exclude one-off cross-sectional studies without either pre-pandemic measures or comparisons of COVID-19 positive vs. unaffected individuals.

**Results:** Searches identified 6,226 articles. Seventy-eight articles met our inclusion criteria. We identified a further 64 relevant cross-sectional studies that did not meet our revised inclusion criteria. Thirty-four articles were not peer-reviewed (e.g. research letters, pre-prints). All articles were based on observational studies.

There was no consistent evidence of a rise in suicide but many studies noted adverse economic effects were evolving. There was evidence of a rise in community distress, fall in hospital presentation for suicidal behaviour and early evidence of an increased frequency of suicidal thoughts in those who had become infected with COVID-19.

**Conclusions:  **Research evidence of the impact of COVID-19 on suicidal behaviour is accumulating rapidly. This living review provides a regular synthesis of the most up-to-date research evidence to guide public health and clinical policy to mitigate the impact of COVID-19 on suicide risk as the longer term impacts of the pandemic on suicide risk are researched.

## Introduction

The COVID-19 pandemic is causing widespread societal disruption, morbidity and loss of life globally. By the end of December 2020 over 85 million people had been infected and over 1.8 million had died ( Worldometers, 2020). There are concerns about the impact of the pandemic on population mental health ( Holmes *et al*., 2020). These stem from the impact of the virus itself on people infected ( Taquet *et al*., 2021), as well as frontline workers caring for them ( Kisely *et al*., 2020) and increases in bereavement. Other concerns relate to the impact on population mental health of the public health measures that have been implemented to minimise the spread of the virus – in particular physical distancing, leading to social isolation, disruption of businesses, services and education and threats to peoples’ livelihoods. Physical distancing measures and lockdowns have resulted in substantial rises in unemployment, falls in GDP and concerns that many nations will enter a prolonged period of deep economic recession.

There are concerns that suicide and self-harm rates may rise during and in the aftermath of the pandemic ( Gunnell *et al*., 2020; Reger *et al*., 2020). Time-series modelling indicated that the 1918–20 Spanish Flu pandemic, which caused well over 20 million deaths worldwide, led to a modest rise in the national suicide rate in the USA ( Wasserman, 1992) and Taiwan ( Chang *et al*., 2020). Likewise, there is some evidence that previous epidemics and pandemics were associated with rises in suicide and suicidal behaviour ( Zortea *et al*., 2020). Suicide rates increased briefly amongst people aged over 65 years in Hong Kong during the 2003 SARS epidemic, predominantly amongst those with more severe physical illness and physical dependency ( Cheung *et al*., 2008).

The current context is, however, very different from previous epidemics and pandemics. The 2003 SARS epidemic was restricted to relatively few countries. Furthermore, during the 100-year period since the 1918–20 influenza pandemic, global and national health systems have improved, international travel and the speed of communication of information (and disinformation) have increased, antibiotics are available to treat secondary infection, and national economies have become globally inter-dependent. The availability of the internet and technological advancement has made it far easier for people to communicate and engage in home working and home schooling. However, there are marked social inequalities in relation to access to technology and ability to stay safe and continue to work, within and between countries. Public health policies and responses, and the degree of access to technology to facilitate online clinical assessments and treatments differ greatly between countries.

Key concerns in relation to suicide prevention during the pandemic include: encouraging help-seeking in those with suicidal thoughts and behaviours e.g. people who have attempted suicide may not attend hospitals because they are worried about contracting COVID-19 or being a burden on the healthcare system at this time; uncertainty regarding how best to assess and support people with suicidal thoughts and behaviours, whilst maintaining physical distancing and addressing any impacts of remote consultation; diminished access to community-based support; exposure to traumatic experiences; long term effect of infection with the virus on mental health ( Taquet *et al*., 2021) and an economic recession may have an adverse impact on suicide rates ( Chang *et al*., 2013; Stuckler *et al*., 2009). There have been increases in bereavement (with many being unusually complicated during the crisis), sales of alcohol ( Finlay & Gilmore, 2020) and domestic violence ( Mahase, 2020) – all risk factors for suicide ( Turecki *et al*., 2019); the insensitive or irresponsible media reporting of suicide deaths associated with COVID-19 may be harmful ( Hawton *et al*., 2021); and in some countries access to highly lethal suicide methods such as firearms and pesticides may rise ( Anestis *et al*., 2021; Gunnell *et al*., 2020). However early findings from high income countries with ‘real-time’ suicide trend data, indicates there was no rise in suicide rates in the early months of the pandemic ( John *et al*., 2020a). Japan is the exception to this rule, falls in Japanese suicide rates in the early months of the pandemic have since been replaced by rises above pre-pandemic levels July/August 2020 and beyond ( John *et al*., 2020a; Tanaka & Okamoto, 2021; Ueda *et al*., 2021). The longer-term impact of the pandemic on suicide deaths and suicidal behaviour remains uncertain.

In the context of the COVID-19 pandemic there is a rapidly expanding evidence base on its impact on suicide rates, and how best to mitigate such effects. It is therefore important that the best available knowledge is made rapidly available to policymakers, public health specialists and clinicians. To facilitate this, we are conducting a living systematic review focusing on incidence and prevention of suicide and self-harm in relation to COVID-19. Living systematic reviews are high-quality, up-to-date online summaries of research that are regularly updated, using efficient, often semi-automated, systems of production ( Elliott *et al*., 2014). Our first report covered the period up to the 7 ^th^ June 2020. This paper reports the second set of findings from the review, based on relevant articles identified up to 19 ^th^ October 2020.

## Aim

The overarching aim of the review is to identify and appraise any newly published evidence from around the world that assesses the impact of the COVID-19 pandemic on suicide deaths, suicidal behaviours, self-harm and suicidal thoughts, or that assesses the effectiveness of strategies to reduce the risk of suicide deaths, suicidal behaviours, self-harm and suicidal thoughts, associated with the COVID-19 pandemic.

## Methods

**Figure 1.**
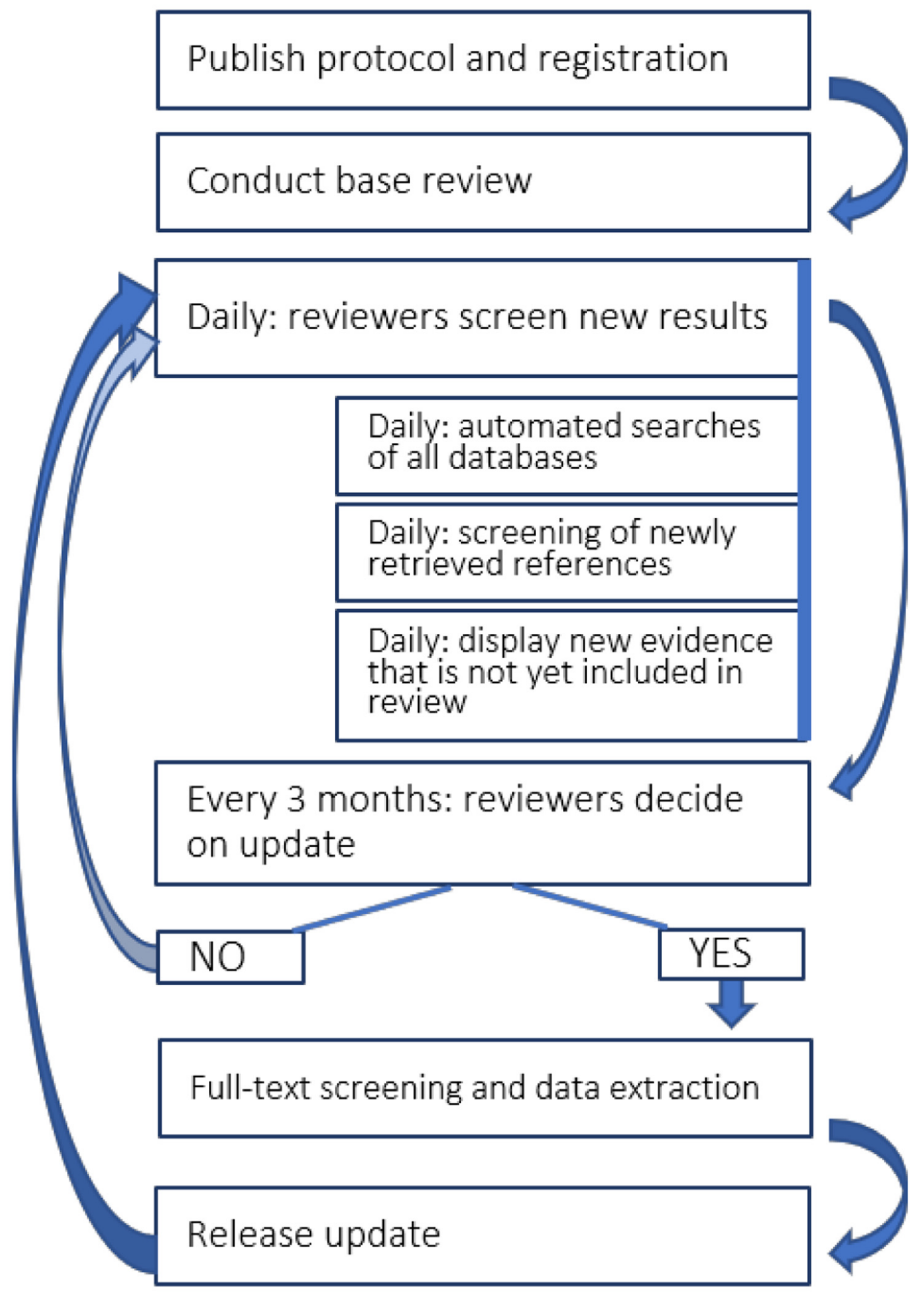
Workflow for updating the living systematic review review. The process will be supported using automation technology and at three-monthly intervals the team will update the published version of the review.

This living systematic review ( Figure 1) follows published guidance for such reviews and for how expedited ‘living’ recommendations should be formulated where relevant ( Akl *et al*., 2017; Elliott *et al*., 2017). The review was prospectively registered (PROSPERO ID CRD42020183326; registered on 1 ^st^ May 2020). An overview of our living review process is provided in Figure 1. A protocol ( John *et al*., 2020b) was published in line with the Preferred Reporting Items for Systematic Review and Meta-Analysis Protocols guideline ( Moher *et al*., 2015) along with the first update of our review which summarised articles identified up to 7 ^th^ June 2020 ( John *et al*., 2020c). Since publication of our protocol we have amended our methodology to: 1) search additionally the PsyArXiv and SocArXiv open access paper repositories; 2) include modelling studies within the scope of our review (e.g. to predict the likely impact of the pandemic on suicide rates); 3) update our research questions to include studying the impact of adult self-neglect and parental neglect and fear of losing livelihood on suicide-related outcomes; 4) update our searches with any new citations from PsycINFO prior to each update; 5) exclude from data extraction and presentation in results tables single-wave, cross-sectional surveys unless they explicitly make comparisons with appropriate pre-pandemic measures or include comparative data between COVID-19 positive and unaffected individuals for pragmatic reasons, due to the volume of such studes but also issues to do with sampling and generalisability of such studies. Surveys that meet the original inclusion criteria are included as an appendix to the update.

## Eligibility criteria

Study participants may be adults or children of any ethnicities living in any country. Outcomes of interest are:

1. Deaths by suicide
2. Self-harm (intentional self-injury or self-poisoning regardless of motivation and intent) or attempted suicide (including hospital attendance and/or admission for these reasons)
3. Suicidal thoughts/ideation

Studies must address one of the following research questions:

(i) What is the prevalence/incidence?

- Prevalence/incidence of each outcome during pandemic (including modelling studies)

(ii) What is the comparative prevalence/incidence?

- Prevalence/incidence of each outcome during pandemic vs not during pandemic

(iii) What are the effects of interventions?

- Effects of public health measures to combat COVID-19 (including physical distancing, school closures, interventions to address loss of income, interventions to tackle domestic violence) on each outcome

- Effects of changed and new approaches to clinical management of (perceived) elevated risk of self-harm or suicide risk on each outcome (any type of intervention is relevant)

(iv) What are the effects of other exposures?

- Impact of media portrayal on each outcome and misinformation attributed to the pandemic on each outcome
- Impact of bereavement from COVID-19 on each outcome
- Impact of any COVID-19 related behaviour changes (domestic violence, alcohol, adult self-neglect, parental neglect, cyberbullying, isolation) on each outcome
- Impact of COVID-19-related workload on crisis lines on each outcome
- Impact of infection with COVID-19 (self or family member) on each outcome
- Impact of changes in availability of analgesics, firearms and pesticides on each outcome (method-specific and overall suicide rates)
- Impact of COVID-19 related socio-economic exposures (changes in fiscal policy; recession/depression: unemployment, debt, fear of losing livelihood, deprivation at the person-, family- or small-area level) on each outcome
- Impact on health and social care professionals: the stigma of working with COVID-19 patients or the (perceived) risk of infection/being a ‘carrier’, as well as work-related stress on each outcome
- Impact of changes in/reduced intensity of treatment for patients with mental health conditions, in particular those with severe psychiatric disorders.
- Impact of any other relevant exposure on our outcomes of interest.

## Qualitative research

We included any qualitative research addressing perceptions or experiences around each outcome in relation to the COVID-19 pandemic (e.g. stigma of infection, isolation measures, complicated bereavement, media reporting, experience of delivering or receiving remote methods of self-harm / suicide risk assessment or provision of treatment; experience of seeking help for individuals in suicidal crisis); narratives provided for precipitating factors for each outcome.

No restrictions were placed on the types of study design eligible for inclusion, except for the exclusion of single-person case reports. Pre-prints will be re-assessed at the time of publication and the most current version included. There was no restriction on language of publication. We drew on a combination of internet-based translation systems and network of colleagues to translate reports in languages other than English.

## Identification of eligible studies

We searched the following electronic databases: PubMed; Scopus; medRxiv, PsyArXiv; SocArXiv; bioRxiv; the COVID-19 Open Research Dataset (CORD-19) by Semantic Scholar and the Allen Institute for AI, which includes relevant records from Microsoft Academic, Elsevier, arXiv and PMC; and the WHO COVID-19 database. A sample search strategy (for PubMed) appears in Box 1 from 1 ^st^ January 2020 to 19 ^th^ October 2020. We have developed a workflow that automates daily searches of these databases, and the code supporting this process can be found at https://github.com/mcguinlu/COVID_suicide_living). Searches are conducted daily via PubMed and Scopus application programme interface and the bioRxiv and medRxiv RSS feeds. Conversion scripts for the daily updated WHO and the weekly updated CORD-19 corpus are used to collect information from the remaining sources. The software includes a systematic search function based on regular expressions to search results retrieved from the WHO, CORD-19 and preprint repositories (search strategy available in extended data). Our review is ongoing and we continue to investigate the use of other databases and to capture articles made available prior to peer review and assess eligibility and review internally. For this update we therefore included PsyArXiv and SocArXiv repositories in our search strategy via their own open access platforms as we developed our automated system. PsycINFO searches were carried out retrospectively on 6 ^th^ January 2021, using a publication date filter for 1 ^st^ January 2020 to 19 ^th^ October 2020.

A two-stage screening process was undertaken to identify studies meeting the eligibility criteria. First, two authors (either CO or EE) assessed citations from the searches and identified potentially relevant titles and abstracts. Second, either DG, AJ or RW assessed the full texts of potentially eligible studies to identify studies to be included in the review. This process was managed via a custom-built online platform (Shiny web app, supported by a MongoDB database). The platform allowed for data extraction via a built-in form.

**Box 1. Search terms for PubMed**

((selfharm*[TIAB] OR self-harm*[TIAB] OR selfinjur*[TIAB] OR self-injur*[TIAB] OR selfmutilat*[TIAB] OR self-mutilat*[TIAB] OR suicid*[TIAB] OR parasuicid*[TIAB) OR (suicide[TIAB] OR suicidal ideation[TIAB] OR attempted suicide[TIAB]) OR (drug overdose[TIAB] OR self?poisoning[TIAB]) OR (self-injurious behavio?r[TIAB] OR self?mutilation[TIAB] OR automutilation[TIAB] OR suicidal behavio?r[TIAB] OR self?destructive behavio?r[TIAB] OR self?immolation[TIAB])) OR (cutt*[TIAB] OR head?bang[TIAB] OR overdose[TIAB] OR self?immolat*[TIAB] OR self?inflict*[TIAB]))) AND ((coronavirus disease?19[TIAB] OR sars?cov?2[TIAB] OR mers?cov[TIAB]) OR (19?ncov[TIAB] OR 2019?ncov[TIAB] OR n?cov[TIAB]) OR ("severe acute respiratory syndrome coronavirus 2" [Supplementary Concept] OR "COVID-19" [Supplementary Concept] OR COVID-19 [tw] OR coronavirus [tw] OR nCoV[TIAB] OR HCoV[TIAB] OR ((virus*[Title] OR coronavirus[Title] OR nCoV[Title] OR infectious[Title] OR HCoV[Title] OR novel[Title])AND (Wuhan[Title] OR China[Title] OR Chinese[Title] OR 2019[Title] OR 19[Title] OR COVID*[Title] OR SARS-Cov-2[Title] OR NCP*[Title]) OR “Coronavirus”[MeSH]))))

## Data collection and assessment of risk of bias

One author (DG, AJ or RW) extracted data from each included study using a piloted data extraction form, and the extracted data were checked by one other author (DG, KH, EA, RC, AJ, or EE where AJ extracted data, AJ where DG extracted data). Disagreements were resolved through discussion, and where this failed, by referral to a third reviewer (KH, NK or PM). Irrespective of study design, data source and outcome measure examined, the following basic information were extracted: citation; study aims and objectives; country/setting; characteristics of participants; methods; outcome measures (related to self-harm / suicidal behaviour and COVID-19); key findings; strengths and limitations; reviewer’s notes. For articles where causal inferences are made - i.e. randomised or non-randomised studies examining the effects of interventions or aetiological epidemiological studies of the effects of specific exposures – we plan to use a suitable version of the ROBINS-I or a preliminary similar tool for exposure studies to assess risk of bias as appropriate based on the research question and study design ( Morgan *et al*., 2017; Sterne *et al*., 2016).

## Data synthesis

We synthesised studies according to themes based on research questions and study design, using tables and narrative. Results were synthesised separately for studies in the general population, in health and social care staff and other at-risk occupations, and in vulnerable populations (e.g. people of older age or those with underlying conditions that predispose them to becoming severely ill or dying after contracting COVID-19) where relevant. Where multiple studies addressed the same research questions, we assessed whether meta-analysis was appropriate and would conduct it where suitable, following standard guidance available in the Cochrane Handbook ( Deeks *et al*., 2019). The current document is the second iteration of our review. We have not considered it appropriate to combine any results identified so far in a meta-analysis due to quality and heterogeneity.

## Results

**Figure 2.**
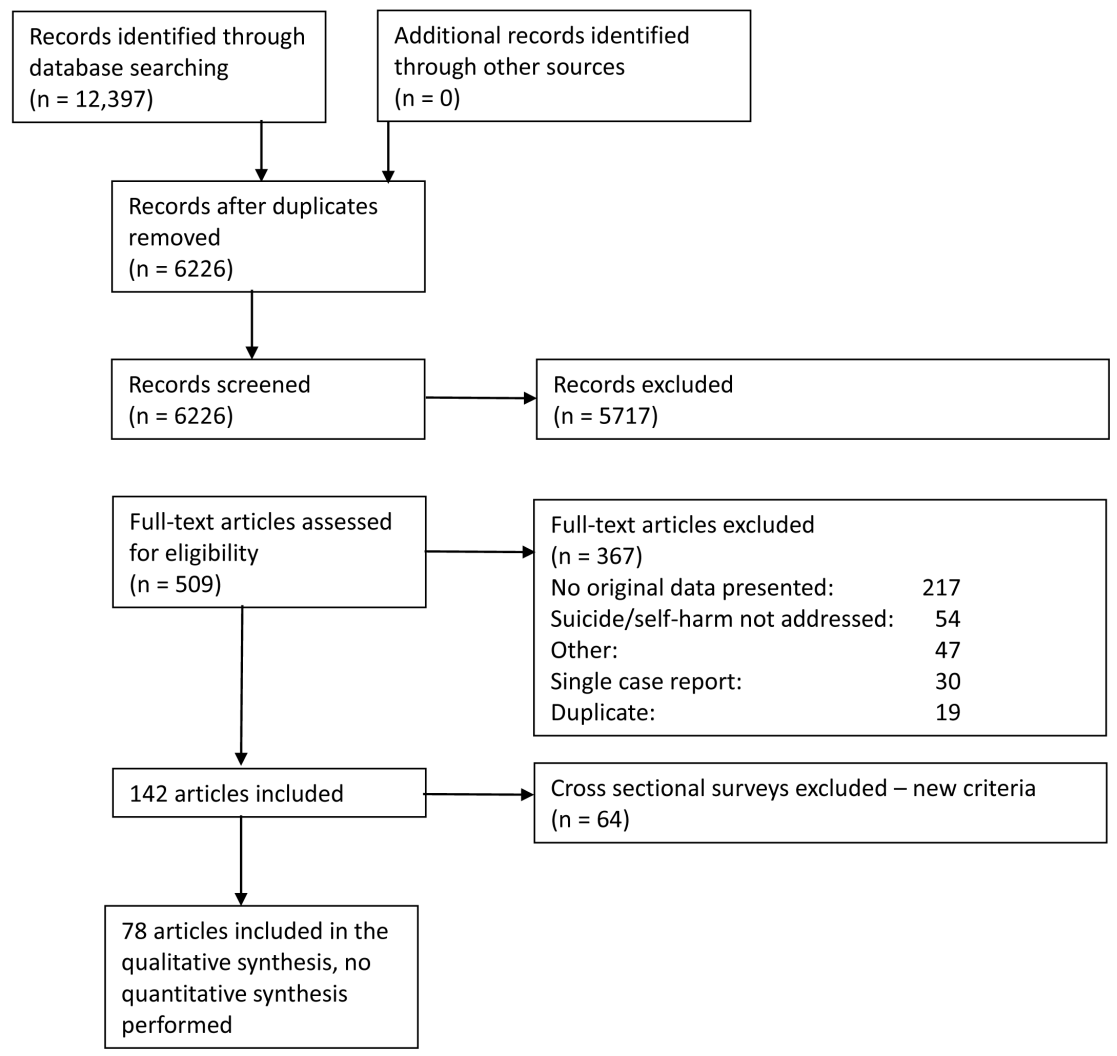
PRISMA flow diagram.

**Figure 3.**
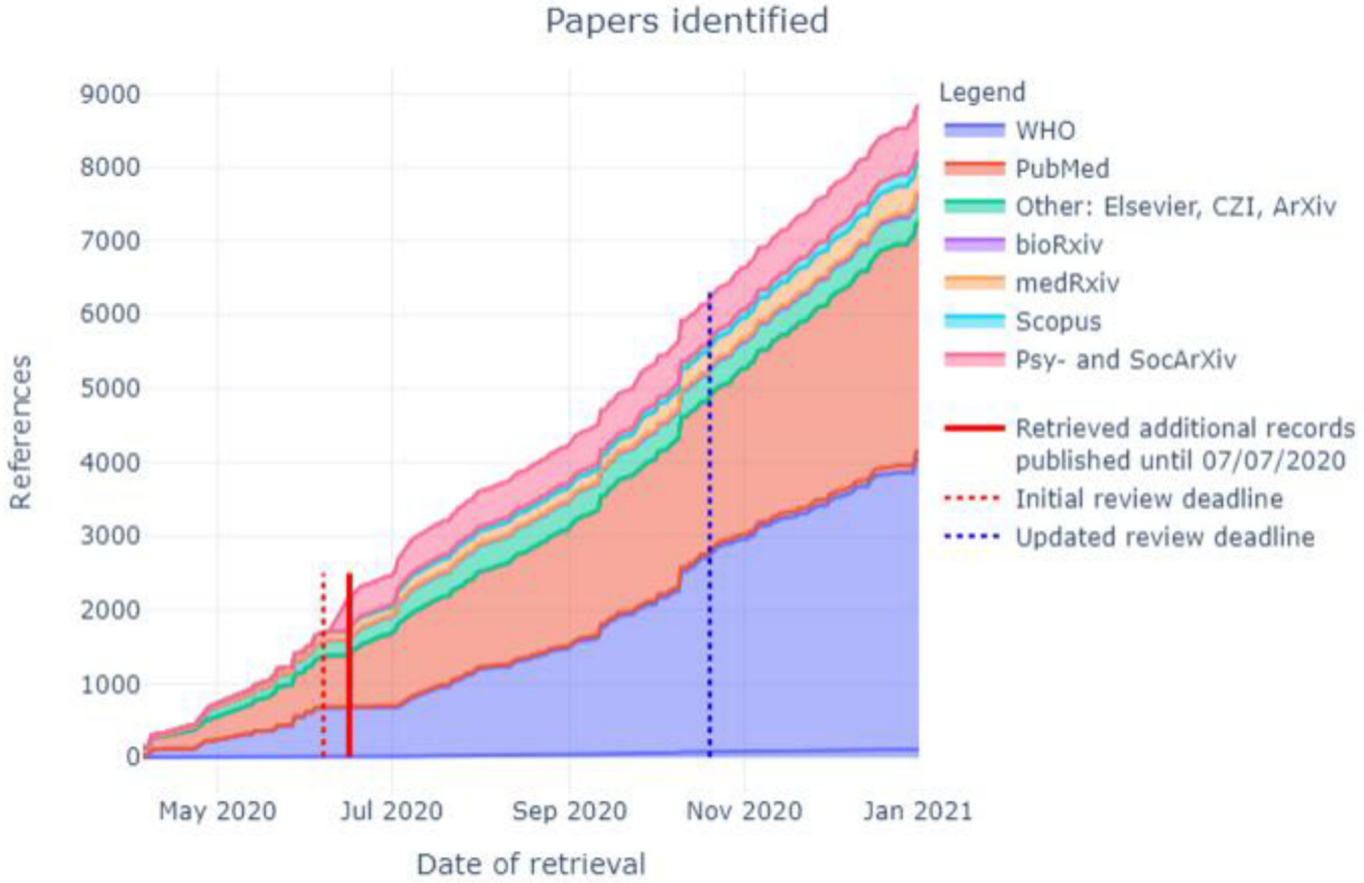
Number of articles identified by database and repository over time.

**Figure 4.**
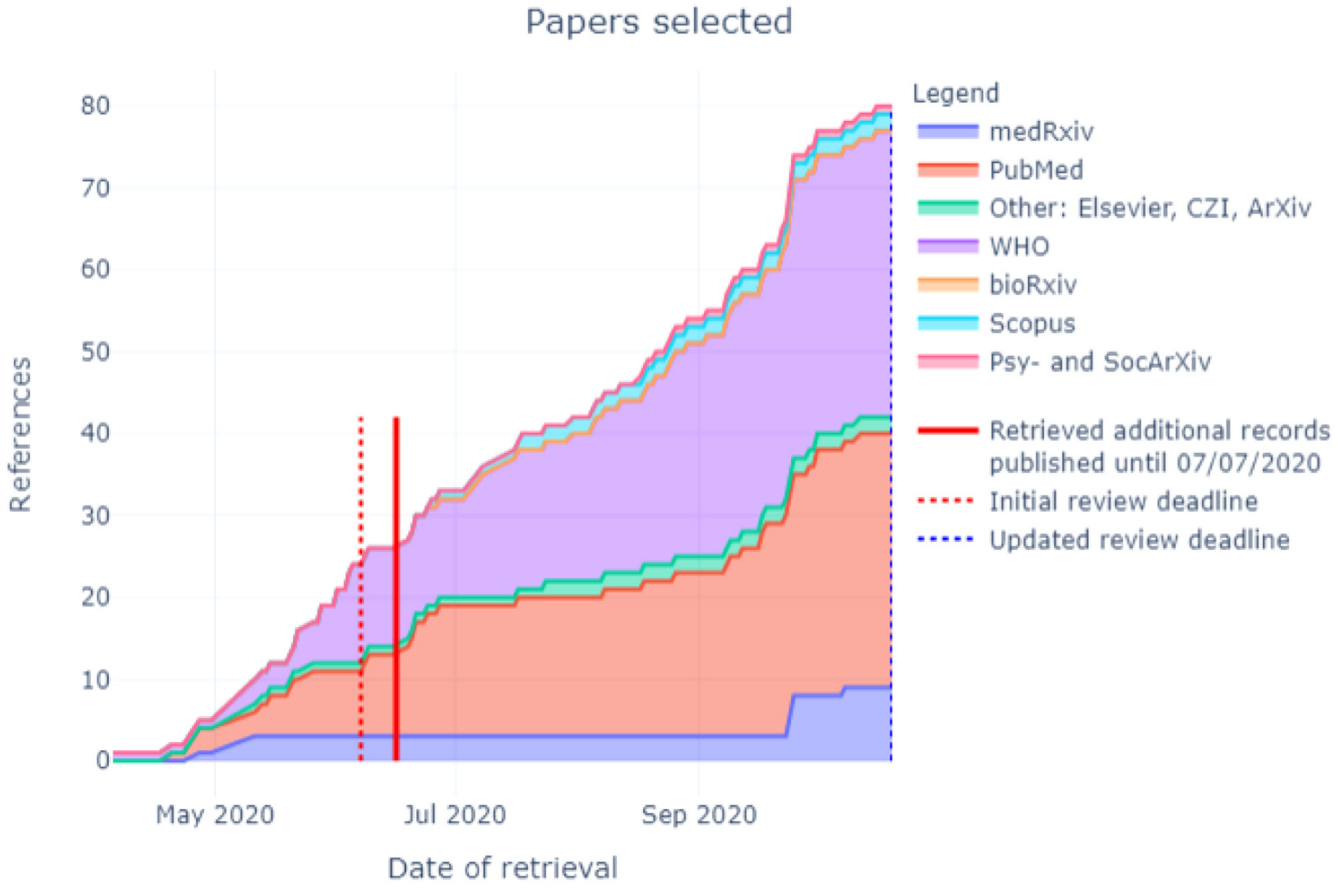
Number of articles selected by database and repository over time.

In total, 12,397 citations were identified by 19 ^th^ October 2020 from all electronic searches, after duplicates were removed ( Figure 2). The cumulative numbers of articles over time that were identified by the search and included in the review are shown in Figure 3 and Figure 4. The majority of studies identified in the review (5105; 82%) were sourced from two databases, PubMed and WHO; a further 10% (n=622) were drawn from pre-print sites such as MedRxiv.

### Description of included studies

We included 78 articles in the review. We have highlighted in Table 1– Table 6 where new citations have updated existing studies. Sixty-four cross sectional surveys are included in Appendix 1. In total, six studies spanned several countries or were worldwide, including one using a Reddit mental health dataset (almost half of users are from the USA); 13 were from the United States; seven from China; nine from India; five from the United Kingdom; four each from Japan and Nepal; and between one and three each from Australia, Bangladesh, Canada, Czech Republic, Denmark, France, Germany, Greece, Iran, Ireland, Israel, Italy, Pakistan, Peru, Poland, Portugal, Spain, Qatar and Switzerland. All articles were based on observational studies: twenty-five were case series with a sample of two or more (although Jefson *et al*., 2020 and Rohde *et al*., 2020 were based on the same case series); thirteen were cross sectional surveys; two were based on social media posts; six were modelling studies; twenty were service utilisation studies; and nine assessed suicide rates. Studies are summarised by these study types in Table 1 through Table 6. Three other relevant articles were identified, two of these described mixed methods studies ( Evans *et al*., 2020; Son *et al*., 2020) and one a case-control study ( Cai *et al*., 2020). Almost half (n=34) of the articles did not appear to have been peer- reviewed of which ten were pre-prints and 21 were published as research letters to the Editor.

**Table 1.** Summary of included case series.

Authors	Geography	Data used	Outcome	Conclusions	Comment/ Limitations
Ahmed *et al.,* 2020	India	Suicide cases linked with alcohol withdrawal syndrome (AWS) reported in newspapers or news channels’ websites from 25 March (start of national lockdown) to 5 May 2020. All cases were in the states of the southern part of India: Kerala, Tamil Nadu, Telangana, and Karnataka. ( **n=23**)	Suicide death	AWS seems implicated in a number of suicides in southern India but, on the basis of the empirical information that is presented here, we do not know whether these deaths were caused by the COVID-19 lockdown, and whether these deaths occurred at a higher frequency during the observation period than they normally occur.	We cannot be sure whether any of the suicides occurred primarily as a direct consequence of AWS, or were brought about due to the unavailability of alcohol during lockdown. Study uses news reports as their data source. Letter to the editor, so unlikely to be peer reviewed.
Bhuiyan *et al.,* 2020	Bangladesh	News reports of COVID-19 related suicide deaths (n=8)	Suicide death	Job loss, debt and difficulties obtaining food because of financial difficulties reported in all cases	Small sample size (n=8) Study uses news reports as their data source. Letter to editor, probably not peer reviewed.
Boshra & Islam, 2020a	Bangladesh	Suicide cases relating to COVID-19 taken from Bangladeshi online media INITIAL REPORT: 1 March to 31 July 2020 (n=32) UPDATED REPORT published October 27th (Boshra & Islam 2020b): 1 March to 30 Sept 2020 ( **n=37**). 65% of the cases were male.	Suicide death	45.9% were due to economic reasons attributed to lockdown-related unemployment.	Although they examined only cases relating to COVID-19, the authors recognize they do not know how many cases would have occurred if the pandemic had not happened. Study uses news reports as their data source. Pre-print, not peer reviewed.
Buschmann & Tsokos, 2020a	Germany	Case series of 10 individuals identified at autopsy who died by suicide during the pandemic up to March 25 ^th^ 2020 UPDATED REPORT ( Buschmann & Tsokas, 2020b) Individuals identified at autopsy who died by suicide associated with the effects of the pandemic up to 29 May 2020 ( **n=11**)	Suicide death	All had pre-existing mental health issues. No evidence of COVID-19. Authors conclude that the effects of the lockdown and media reporting influenced the suicide.	It is unclear what circumstances of the deceased persons were brought about directly due to the COVID-19 crisis. Both are Letters to editor, probably not peer reviewed.
Dsouza *et al.,* 2020	India	News reports (n=69) of COVID-19 related suicide deaths including **n=72** cases from March to 24 May 2020. Age range 19–65 years; 63 (88%) males.	Suicide death	The most common reported factors were: 1) Fear of infection (n=21); 2) Financial crisis (n=19); 3) COVID-19 related stress (n=9); 4) Positive test for COVID-19 (n=7); 5) Isolation related issues (n=5) 6)Social boycott (n=4); and 7) Migrant unable to return home (n=3).	Study uses news reports as their data source. Overlaps with other publications based on news reports from same country e.g. Rajkumar, 2020; Shiob *et al.,* 2020. Letter to editor, probably not peer reviewed.
Griffiths & Mamun, 2020	Global -Bangladesh, India, Malaysia, USA	News reports of couples ( **n=6**) engaging in COVID-19-related suicidal behaviour includes one murder suicide identified via Searches of seven English- Indian online papers from March to 24 May.	Suicide attempt and/or death (couples)	Details several potential reasons: 1) Fear of infection; 2) Money problems (due to recession associated with lockdowns); 3) Harassment or victimisation by others due to (possibly perceived) infection status; 4) Stress of being in isolation or quarantine; and 5) Uncertainty of when the pandemic will end.	Small sample size (n=6) Study uses news reports as their data source. Letter to editor, probably not peer reviewed.
Iqbal *et al.,* 2020	Qatar	Referrals of patients with a positive COVID-19 test to consultant liaison psychiatry service from a ward or A&E in three hospitals in Doha, . Median age 39.5; 48 male ( **n=50**)	Suicide attempts/self- harm	Three of the 50 referrals had self harmed. The self-harm was apparently a reaction to the pandemic. Two were asymptomatic for COVID-19, and one had a mild case.	Focus is on psychiatric presentations in people with acute infections, the long term impact of COVID-19 infection on psychiatric morbidity requires further study. Peer reviewed.
Jefsen *et al.,* 2020a Rohde *et al.,* 2020	Denmark	Review of notes of adult patients from the psychiatric services of the Central Denmark Region (catchment area: 1.3 million people). Notes between 1 Feb and 23 March 2020 reviewed to identify those describing "pandemic-related psychiatric symptoms" (including "self harm / suicidality", **n=74**). Median age 29.8 years; 77% female Note full case series n=1357 relevant records found from 412,804, reported in Rohde *et al.,*, 2020.	Suicide attempts/self- harm, suicidal thoughts	Of the 74 patients identified, 14 (19%) had self-harm thoughts; 10 (14%) had self-harmed; 34 (46%) had suicidal thoughts; 10 (14%) had made suicide attempts and 13 (18%) had a passive wish to die from COVID-19.	Findings restricted to suicidal / self-harm related outcomes in 74 patients with these outcomes. No data on the overall percentage of adult psychiatry patients with these outcomes during or pre-pandemic. Peer-reviewed letter to the editor.
Jefsen *et al.,* 2020b	Denmark	All clinical notes from patients below 18 years old in the Central Danish psychiatric service between 1 Feb and 23 March 2020. Pandemic‐related psychopathology identified in 94 children and adolescents.	Suicide attempts/self- harm	8 notes from 5 of the 94 patients specifically described self‐harm or suicidality related to the pandemic	No baseline data for individuals. No data on the overall percentage of child psychiatry patients with these outcomes during or pre-pandemic. Editorial perspective; probably not peer reviewed.
Jolly *et al.,* 2020	USA	Child and adolescent psychiatry inpatients, age range 11–17 years; 3 female, 1 male; ( **n=4**).	Suicide attempts/self- harm, Suicidal thoughts	One suicide attempt; one suicidal plan and two with suicidal thoughts Stressors described included: 1. Unable to see friends/ partner (all cases) 2. Arguments with parents 3. Misunderstanding within friendship group that could not be resolved well over social media 4. Academic worries- performance declined since move to distance learning 5. Feeling isolated	Detailed descriptive study of very small sample. Peer reviewed journal.
Kapilan, 2020	India	News reports about two nurses drawn from news reports ( **n=2**)	Suicide death, Suicide attempts/self- harm	1 suicide: a nurse who treated COVID-19 patients, and died reportedly due to “ extreme stress and mental disturbance” 1 suicide attempt: a nurse who contracted COVID-19	Small sample size (n=2) Information drawn from news reports. Similar to Rahman *et al.,* 2020. Letter to the editor; possibly not peer reviewed.
Kar *et al.,* 2020	India	News reports of deaths by suicide among film stars in India, 28 May to 30 July 2020 ( **n=7**).	Suicide death	7 Indian film stars who died by suicide. Media reports claimed three of these were related to financial problems associated with COVID-19.	It is unclear whether any of the deaths were strongly linked with COVID-19 and its indirect impact on people's lives, or whether the individuals were already experiencing mental health difficulties. Study uses news reports as their data source. Appears to use the same data as Mamun *et al.,* 2020b. Letter to the editor; probably not peer reviewed.
Mamun *et al.,* 2020a	Bangladesh	News report of suicide pact in mother and 22 year old son, 11 Jun 2020 ( **n=2**)	Suicide death	University student aged 22 and his mother aged 47 died by suicide. The father had insisted the day before that the student complete online exams as an internet connection was arranged.	Study uses news reports as their data source. Only a single pact reported Suggests that online teaching in LMIC may create real tensions due to digital poverty Letter to the editor; possibly not peer reviewed.
Mamun *et al.,* 2020b	India	News reports of deaths by suicide among film stars in India ( **n=7 in 2020 ** **vs.** n=16 in 2002–2019)	Suicide death	The frequency of celebrity suicides in India appears to have increased markedly during the COVID-19 era. The authors highlight the dangers of sensationalised media reporting of celebrity suicides triggering immitative events in the general population.	Study uses news reports as their data source. Appears to use the same data as Kar *et al.,* 2020 Letter to the editor; possibly not peer reviewed.
Mamun & Ullah, 2020	Pakistan	News reports of COVID-19 related suicide deaths in Pakistan, Jan 2020 to end April 2020 ( **n=12**, a further 4 reports of suspected suicide were not presented).	Suicide death	Economic concerns reported in 8/12 cases, and fear of infection in the remaining 4. There were 13 other reports of suicides (and attempted suicide) during this period not reported to be linked to COVID-19.	Highlights the potential importance of the economic impact of COVID-19 and/or public health measures on influencing suicide in low- and middle-income countries. Study uses news reports as their data source. Peer reviewed journal; paper accepted on same day as received.
Nalleballe *et al.,* 2020	World	Adult COVID-19 patients (inpatients and outpatients) with records held on the TriNetX database ( trinetx.com), 20 Jan to 10 June 2020 (n= 40,469, 76% living in USA	Suicidal thoughts	9,086 (22.5%) had a neuropsychiatric coded diagnosis within 1 month of COVID-19 diagnosis. 62 (0.2%) had suicidal thoughts recorded.	Large clinical database of people with clinical diagnosis of COVID-19. It is possible that suicidal thoughts were not asked about systematically by clinicians and so there is likely to be marked under-recording. Peer reviewed journal.
Pirnia *et al.,* 2020	Iran	Suicide of members of one family ( **n=2**).	Suicide death	Son died by suicide three weeks after his father died of COVID-19. Two days after the son, the mother also killed herself.	Small sample size (n=-2). Letter to the editor; probably not peer reviewed.
Rahman *et al.,* 2020	Worldwide	News reports of nurse suicide deaths ( **n=6**, 2 from Italy, 1 each from UK, Mexico, USA and India)	Suicide death	Factors reported as associated with deaths included: fear they had become infected; positive test result; being in quarantine; fearful of becoming infected.	Study uses news reports as their data source. Small sample size (n=6). Similar to Kapilan, 2020. Peer reviewed letter to the editor.
Rajkumar, 2020	India	49 English-language news reports of COVID-19 related suicides in India, 12 March to 11 April 2020 ( **n=23** deaths)	Suicide death	6 of the deaths occurred amongst patients hospitalised / in isolation In 7 cases a diagnosis was mentioned - in 4 this was depression, in 3 alcohol dependence. Precipitating / contributing factors included fear of acquiring infection (9/23); developing influenza-like symptoms (7/23); bereavement (n=5)	Study uses news reports In English as their data source. Provides interesting observations, useful for hypothesis testing. Probable overlap with others e.g. Dsouza *et al.,* 2020; Shiob *et al.,* 2020 Letter to the editor, possibly not peer reviewed.
Sahoo *et al.,* 2020	India	Clinical case reports of COVID-19 related suicide attempts presenting to the ED ( **n=2**)	Suicide attempts	Both cases were related to the fear and stigma of COVID-19. One case was ordered to self-isolate due to being in contact with a known case.	Small sample size (n=2) Letter to editor; probably not peer reviewed.
Shoib *et al.,* 2020	India	News reports in 22 English and local newspapers published in India, identified from Google and reporting on suicides in relation to COVID-19 Search period 25 Jan to 18 April 2020 ( **n=34** suicides)	Suicide death	18 (52.9%) aged 18–35 years; 28 (82.4%) male Most frequent reasons given: Fear of infection: 16 (47.1%); misinterpreted fever as COVID-19: 9 (26.5%); Depression and loneliness: 7 (20.6%); personal stigma of COVID-19: 4 (11.8%) Authors mapped number of reports vs number of suicides over the 8 week study period. Rise in COVID-19 related suicides mirrored the rise in number of cases - in first 3 weeks there was 1 report per week, whereas in the last 3 weeks there were 23 reports	Large case series of news reports, but probably overlaps with others e.g. Dsouza *et al.,* 2020; Rajkumar, 2020. Study uses news reports as their data source. Letter to the editor, possibly not peer reviewed.
Syed *et al.,* 2020	India	Reports of alcohol-related suicides from India, extracted from recent media reports, using Google News, retrieving reports of suicide cases from Indian online English language newspapers between 25 March and 17 May 2020 (during India’s national lockdown). Age range 25–70 years; all males ( **n=27**)	Suicide death, Suicide attempts	27 cases suicide or suicide attempts. Alcohol restrictions were reported as leading to an increase in attempts and deaths, because of alcohol withdrawal syndrome.	Case reports from newspapers in English in Indian news. Underreporting possible because of stigma. Similar to Shiob *et al.,* 2020. Letter to the editor; possibly not peer reviewed.
Thakur & Jain, 2020	World	News reports of COVID-19 related suicide deaths ( **n=7**)	Suicide death	Identified 4 types of suicide risks: 1) Social isolation; 2) Economic; 3) Stress in health professionals; 4) Stigma	Small sample size (n=7) Study uses news reports as their data source. Peer reviewed journal; paper accepted 1 day after received.
Valdés-Florido *et al.,* 2020	Spain	Patients admitted to two hospitals in Spain with reactive psychoses in the context of the COVID-19 crisis during the first two weeks of lockdown ( **n=4**)	Suicide attempts	Stress from the pandemic thought to have triggered reactive psychoses in four patients two of whom presented with severe suicidal behaviour	Small sample size (n=4) Peer reviewed journal.

**Table 2.** Summary of cross sectional surveys and cohort studies.

Authors	Geography	Data used	Outcome	Conclusions	Comment/ Limitations
Debowska *et al.,* 2020	Poland	University students recruited via 10 Polish universities and the Students’ Parliament of the Republic of Poland. N = 7228, 81% female; Mean age = 22.78. Data collection occurred in five waves, during the first two months of the COVID- 19 pandemic in Europe (March – April 2020). The waves differed from one another in the amount and type of lockdown-type measures, with wave 4 being characterised by the strictest restrictions	Suicidal thoughts	No statistical evidence of differences in suicidal thoughts over the 5 stages of data collection or of gender differences in prevalence.	Representativeness of sample unclear Frequency and intensity of suicidal thoughts and impulses in the past 24 h were measured using the Depressive Symptom Inventory-Suicidality Subscale ( Joiner *et al.*, 2002) Letter to editor, probably not peer reviewed
Hamm *et al.,* 2020	USA	Subset of adults aged >60 years who were participating in an RCT of treatment resistant depression and agreed to a qualitative interview. N=73 (of total 743 RCT participants)	Suicide and self-harm thoughts	5(7%) had suicidal thoghts at the time of the interview (April 1–23 2020), but not pre-pandemic; 7 (10%) had had a reduction in pre-existing suicidal thoughts. The rest had no suicidal thoughts pre or post pandemic	Used PHQ-9 pre and post pandemic (validated measure) Those agreeing to interview self- selecting, perhaps less likely to have experienced untoward effects. Small sample Peer reviewed
Hamza *et al.,* 2021	Canada	Students at a single university in Canada Surveyed using the same survey tool in May 2019 and May 2020. n=773 (74% female; mean age 18.5 years) ( 964 responders to 2019 survey)	Suicide attempts/ self-harm	No statistical evidence evidence of rise in NSSI: score at T1 (May 2019) 0.18 (SD 0.38) and T 2 (May 2020) 0.20 (SD 0.40) Likewise no difference when analysis stratified according to presence of absence of pre-existing mental health concerns	Used adapted version of the Inventory of Statements about Self-Injury (ISAS; Klonsky & Glenn, 2009) to assess non-suicidal self-harm in relation to 7 behaviours e.g. cutting / biting. Reported average score on ISAS scale rather than prevalence of each / any behaviour Peer reviewed
Iob *et al.,* 2020	UK	General population sample recruited on- line via media / social media. Survey data from 21 March – 20 April 2020. Participants included individuals who provided data on abuse, self-harm and thoughts of suicide or self-harm on at least one occasion n = 44 775 Weighted to represent UK population (age, sex, ethnicity, education)	Suicide attempts/ selfharm, suicide and self-harm thoughts Help seeking	7984 (18%) reported suicidal / self- harm thoughts; 2174 (5%) had self harmed at least once. Suicide/self-harm thoughts higher in those with a COVID-19 diagnosis vs. without (33% vs 17%); likewise for suicide attempts (14% vs. 5%). 57% of those engaging in SH and 40% with thoughts had sought some professional support. Compared with previous UK survey data, levels of help-seeking from MH professionals (14.5% for thoughts / 4.7% SH/SA) were lower. (14.5% for thoughts / 4.7% SH/SA) were lower.	Suicidal / self-harm thoughts measured via PHQ-9. Self harm via asking participants whether they’d self-harmed or deliberately hurt themselves. Index period was the last week. Large sample but convenience sampling Use of sample weighting to take account of selection bias Report on outcomes in relation to COVID-19 diagnosis but may be confounded by sociodemographic differences between groups Peer reviewed
Raifman *et al.,* 2020	USA	Two nationally representative surveys of US adults: 1) The 2017–2018 National Health and Nutrition Examination Survey (NHANES)- 5085 (86.8%) of 5856 NHANES participants responded to suicidal ideation questions and were included in the analyses; 2) 2020 COVID-19 and Life Stressors Impact on Mental Health and Wellbeing study (CLIMB) - conducted 31st March to 13th April 2020. 1415 (96.3%) of 1470 CLIMB participants responded to all questions relevant to the analysis	Suicidal thoughts	Suicidal ideation increased more than fourfold, from 3.4% in the 2017–2018 NHANES to 16.3% in the 2020 CLIMB survey, and from 5.8% to 26.4% among participants in low-income households. Suicidal ideation was more prevalent among people facing difficulty paying rent (31.5%), job loss (24.1%), and loneliness (25.1%).	Survey methods for NHANES and CLIMB were not identical, but two large population-based surveys conducted at two points. Characteristics of participants in CLIMB and NHANES differed. Respondents may have differed from those who did not, particularly if the stressors examined affected survey participation. Pre-print, not peer reviewed
Sueki & Ueda, 2020	Japan	Two wave population survey of Japanese people aged >20. Recruited via Internet Survey company to reflect census population of Japan. 6683 completed both waves of the survey (out of 125,011 people selected (5%) and 67% of the 9982 who completed the wave 1 survey) 51% male; mean age 46.5 years. Surveyed Jan 24 2020 (when there were just 2 covid-19 cases in Japan) and again 27–30 April, 3 weeks after state of emergency declared.	Suicidal thoughts	Suicidal thoughts score was lower during the pandemic (mean = 1.59) than before it (mean = 1.71),t(6682) = 5.87, p < .001. People in their 30s, and people: a) with unstable employment status (part-time, temporary worker), b) without children, c) with relatively low annual household income and d) those currently receiving psychiatric care had higher suicidal thoughts scores at T2 vs. the reference group, after controlling for suicidal ideation at T1	Short-form suicide ideation scale" ( Sueki, 2019). 6 questions, overall scores ranges from 0–12. Low response rate from selected sample (5%) And at T2 vs T1 (67%). Pre-print, not peer reviewed
Wang *et al.,* 2020a	China	COVID 19 patients and controls January 2, 2020 to March 10, 2020. 376 COVID-19 patients (including 95 male and 281 female patients) hospitalized between January 2 and March10, 2020，with 501 controls without COVID 19 (including 110 men and 391 women) recruited from different social media platforms	Suicidal thoughts	In Covid-19 patients moderate or high suicide risk in 27 % COVID-19 patients vs. 8 % in control (sig difference). High or very high suicide risk similarly higher in Covid group 10% vs. 4%. Age, anxiety, depression and poor sleep quality were all risk factors for high suicide risk in COVID-19 patients.	Online or face to face interview assessment by psychiatrists using the Nurses’ Global Asesment of Suicide Risk scale(NGASR). Convenience sampled controls Unlikely to be peer reviewed
Wang *et al.,* 2020b	China	Repeat cross sectional study. Participants who completed survey via “Wenjuanxing,” a Chinese online platform providing functions equivalent to Qualtrics. The data were from two studies, one conducted during the outbreak stage from (N=2540, mean age = 25.28 ± 8.07) and one conducted during the after peak stage (N=2543, mean age = 22.03 ± 6.30)	Symptom networks illustrating the relationship between depression and anxiety symptoms were estimated Suicidal thoughts showed a decreased connection with “inability to relax” and “guilty” symptoms, whereas suicidal thoughts showed an increased connection with the “too much worry” symptom over time	The association between symptoms changed over the course of the pandemic in China Some changes in connections between some symptoms of suicidal thoughts and other symptoms of depression/anxiety If generalizable, could point to some treatment targets that are more central to suicide risk	Limitation: anxiety and depression assessed via self-report not diagnoses Used PHQ-9 Not certain how generalizable networks are to other phases of the pandemic or to other countries Peer reviewed
Winkler *et al.,* 2020	Czech Republic	Covid-19 survey 6th to 20th May 2020.: N=3021 respondents interviewed either by computer-assisted telephone interview or computer assisted web interviewing. General population aged 18–64 years. The survey was representative in relation to national population (age, sex, education and region) Comparable baseline data were obtained from the 2017 Czech Mental Health Survey.	Suicide risk	Marked increase in respondents with moderate/high suicide risk from 3.9% (95% CI 3.2, 4.5) in 2017 to 12.3 (11.1, 13.4) in 2020. Having been tested for Covid-19 (with a positive or negative result) was linked with elevated perceived suicide risk (OR 2.1; 1.1, 3.8) as was Covid-19 health worries (OR 1.4; 1.1, 2.1) and Covid-19 economic worries (OR 1.4; 1.2, 1.7).	Mini International Neuropsychiatric Interview (MINI) Large nationally representative survey with comparable baseline data but Covid-19 survey was conducted remotely whereas the baseline survey was face-to-face interviewing, so information bias cannot be ruled out. Computer- assisted telephone interviewing had a low participation rate. Peer reviewed
Wu *et al.,* 2020a	China	Survivors of COVID-19, followed up median 22 days (IQR 20–30d) post hospital discharge. N=370	Suicide and Self-harm thoughts	4 (1.1%) reported experiencing suicidal / self-harm thoughts over several days	Large survey of hospital admitted COVID-19 No pre-illness baseline measure. Used PHQ-9 (standardised measure). Letter to editor, probably not peer reviewed.
Wu *el al.*, 2020b	China	4124 pregnant women during their third trimester from 25 public hospitals in 10 provinces Jan 1 ^st^-Feb 9 ^th^ 2020 1285 assessed after January 20, 2020 when the coronavirus epidemic was publicly announced and 2839 were assessed before this time point.	Self-harm thoughts	A multi-centre study to identify mental health concerns in pregnancy The risk of self- harm thoughts was higher after 20 ^th^ January compared to before (aRR=2.85, 95% CI: 1.70, 8.85, P=0.005).	Pre-existing data collection system. Thoughts of self-harm in the last 7 days from the Edinburgh Postnatal Depression Scale (EPDS, Cox *et al.,*., 1987) The findings indicate a need for enhanced levels of psychological support for pregnant women during a major infectious disease epidemic / pandemic. Pregnant women in Wuhan, Hubei Province (the epicentre of the epidemic) were not included in the sample. Peer reviewed
Zhao *et al.,* 2020	China	Survey of COVID-19 patients (n=106), 46 male, range 35–92 years at Tongji Hospital, Wuhan from Carried out February 2nd- 16 ^th^, 2020	Suicide and Self-harm thoughts	24.5% (26/106) of COVID-19 patients had self-harming or suicidal thoughts, which were "significantly higher percentages than those of the general population."	Highlights the potential mental health support needs, and the risk faced by recovering COVID-19 patients. Used PHQ-9. Peer reviewed
Zhang *et al.,* 2020	China	Repeated survey in cohort of primary and secondary school children / adolescents from two counties before the outbreak started (wave 1, early November 2019) and 2 weeks after school reopening (wave 2, mid-May 2020) in an area of China with low risk of COVID-19. 1389 children recruited 1271 completed info for W1. 1241 W2, response rate 93.1%. Mean [SD] age, 12.6 [1.4] years; age range, 9.3–15.9 years; 736 [59.3%] male).	NSSI Suicidal thoughts Suicide plans	NSSI (42.0% in 2020 vs 31.8% in 2019; aOR, 1.35 [95% CI, 1.17-1.55]; P < .001), suicide ideation (29.7% vs 22.5%; aOR, 1.32 [95% CI, 1.08-1.62]; P = .008), suicide plan (14.6% vs 8.7%; aOR, 1.71 [95% CI, 1.31-2.24]; P < .001), and suicide attempt (6.4% vs 3.0%; aOR, 1.74 [95% CI, 1.14-2.67]; P < .001). OR adjusted for sex, body mass index, self-perceived household economic status, family cohesion, parental conflict, academic stress, parental educational level, family adverse life events, self-perceived health, sleep duration, and sleep disorders	For NSSI, asking ‘In the past 12 months, have you ever harmed yourself in a way that was deliberate, but not intended to take your life?’. Suicidal ideation, plans and attempts- from the 2013 Youth Risk Behaviour Surveillance System in the USA Pre-covid data Total number of children in years 4–8 not given so not sure of % recruited and therefore representativeness Seasonal variations and secular trends not accounted for. Peer reviewed

**Table 3.** Summary of social media platform posts studies.

Authors	Geography	Data used	Outcome	Conclusions	Comment/ Limitations
Low *et al.*, 2020	Demographic information is unknown but Reddit users are predominantly American (49.9%)	Reddit Mental Health Dataset including posts from 826,961 unique users from 2018 to 2020.	Using unsupervised clustering, they found the suicidality and loneliness clusters more than doubled in the number of posts during the pandemic. The Reddit support groups for borderline personality disorder and posttraumatic stress disorder became significantly associated with the suicidality cluster The suicidality cluster doubled in size and a new cluster surrounding self-harm emerged.	Using natural language processing (NLP) on text from some of the world’s largest mental health support groups it is possible to identify mental health problems as they emerge in real time and to identify vulnerable sub-groups	Such approaches could help subreddit moderators track who is in need of assistance as well as well the concerns of specific communities are No formal diagnoses are made, reliant on what authors post Selection bias related to who posts as well as when they post and how they cope under different circumstances Peer reviewed
Saha *et al.*, 2020	USA	∼60M Twitter streaming posts originating from the U.S. from 24 March-24 May 2020, and compare these with ∼40M posts from the comparable period in 2019	A 20% increase in frequency of posts that made reference to suicidal ideation was observed during 2020.	Suicide risk is multifaceted. More attention directed at population-scale mental healthcare, such as universal screening approaches	Analysis of Twitter content makes good use of readily available data and may reveal patterns and trends that are not easily discernible by conducting research using more traditional methods but what state in their posts does not necessarily reflect trends in suicidality in the population. Not peer reviewed. Pre-print.

**Table 4.** Summary of studies using modelling approaches to estimate the possible impact of the pandemic on suicide rates.

Authors	Country / region	Data used to inform estimate	Model prediction	Comment / Limitations
Bhatia, 2020a	USA	Previous research modelling the association of unemployment with suicide in the USA indicating a 1% rise in unemployment was associated with a 1% rise in suicide. Assumes unemployment in the USA has risen from 3.8% to over 20%	7444 additional suicides in the following 2 months There were approximately 48,000 suicides in USA in 2018, so this equates to a predicted 15% rise in suicides in the USA.	No account for potential impacts of pandemic other than via unemployment rises Duration of unemployment rises uncertain Pre-print, not peer reviewed.
Bhatia, 2020b	USA	Meta-analysis of longitudinal studies investigating the association of duration of unemployment with risk of suicide: used estimate of 2.5 fold increase in risk during 1–5 years of unemployment, derived from one Swedish and one Finnish cohort. National bureau of Health statistics: age adjusted suicide rates US Dept of Labour: weekly unemployment claims US Bureau of Labour Statistics: are distribution of workforce	Estimated 9,786 additional suicides per year There were approximately 48,000 suicides in USA in 2018, so this equates to a predicted 20% rise in suicides in the USA	Estimate of the association between unemployment and suicide derived from person-based studies investigating long-term unemployment and risk of suicide; this may over-estimate association in the context of economic recession Unclear whether age specific suicide risks were applied to the unemployment data – these were not reported in meta-analysis and text of paper contradictory No account for potential impacts of pandemic other than via unemployment rises Pre-print, not peer reviewed.
Kawohl & Nordt, 2020	World	Previous research modelling the association of unemployment with suicide in 63 countries (2000– 2011). International Labour Organisations (ILO) Predicted job losses (March 2020) of between 5.3 to 24.7 million	Between 2135 and 9570 extra suicides per year worldwide. i.e. a 0.3% to 1.2% rise	No account for potential impacts of pandemic other than via unemployment rises Duration of unemployment rises uncertain Research letter, probably not peer reviewed.
McIntyre & Lee, 2020a	USA	The authors analysed theassociation of unemployment with suicide in the USA (1999–2018) and reported a 1% rise in unemployment was associated with a 1% rise in suicide. Three scenarios for changes in level of unemployment a) unchanged at 3.6%(2020), 3.7% (2021); b) rise to 5.8% (2020) and 9.3% (2021); c) rise to 24% (2020) and 18% (2021).	Scenario b) associated with a 3.3% rise in suicide in 2020–21 Scenario c) associated with an 8.4% rise in suicide in 2020–21.	Usefully models the potential impact of two different unemployment rate rises. No account for potential impacts of pandemic other than via unemployment rises Duration of unemployment rises uncertain Peer reviewed
McIntyre & Lee, 2020b	Canada	The authors analysed the association of unemployment with suicide in Canada (2000–2018) and reported a 1% rise in unemployment was associated with a 1% rise in suicide. Three scenarios for changes in level of unemployment a) minimal change at 5.9%(2020), 6.0% (2021); b) rise to 8.3% (2020) and 8.1% (2021); c) rise to 16.6% (2020) and 14.9% (2021).	Scenario b) associated with a 5.5% rise in suicide in 2020–21 Scenario c) associated with a 27.7% rise in suicide in 2020–21.	Usefully models the potential impact of two different unemployment rate rises. No account for potential impacts of pandemic other than via unemployment rises Duration of unemployment rises uncertain Peer reviewed
Moser *et al.,* 2020	Switzerland	Used published data on increased risk of suicide amongst a) prisoners in shared cells (3 fold increased risk) and b) prisoners in solitary confinement (27 fold increased risk) as indicators of risk of lock down on a) multi-person households and; b) single person households. Data on the annual number of suicides in Switzerland and the proportion of Swiss people living alone (16%) and in shared households (84%).	Estimate 1523 additional suicides. Based on an estimate the 1043 recorded suicides in Switzerland in 2017 this equates to a more than doubling in suicides deaths	The team modelled the impact of COVID-19 pandemic on multiple outcomes as well as suicide. Prison confinement is probably not a good proxy for effects of lockdown. High suicide rates in prisoners are due to multiple factors e.g. age and gender profile; high levels of psychiatric morbidity rather than impacts of confinement. Other potential factors e.g. rises in unemployment not included in models Pre-print, not peer reviewed.

**Table 5.** Summary of studies assessing service utilisation.

Authors	Country / region	Data used	Outcome	Findings	Comment / Limitations
Capuzzi *et al.,* 2020	Italy	Emergency psychiatric evaluations at psychiatric emergency rooms in two centres in Lombardy, serving a population of approx. 850,000 in two equivalent periods pre (Fri 22 Feb 2019-Sun 5 May 2019) and following the first COVID-19 case in Italy up to end of first phase of lock-down (Fri 21 Feb 2020 to Sun 3rd May 2020). Data obtained from hospital registers.	Suicide attempts/self- harm	Period A (2019) 388 total attendances, including 68 (17.5%) for self-harm/suicide attempt Period B (2020) 225 total attendances, including 59 (26.2%) for self-harm/suicide attempt. Whilst absolute number of SH/SA cases lower, the difference in number as a proportion of total cases was somewhat higher in age/sex adjusted models (aOR 1.48 (0.97 to 2.28)	Hospital based study from two centres Peer reviewed
Chen *et al.,* 2020	England, UK	Data obtained from Trust hospitals clinical record systems. People using or referred to inpatient and community MH services (including psychological therapy services) in Cambridge and Peterborough - population approx 860,000. Data for Liaison psychiatry referrals for SH/Suicide attempt/ cover 11 March 2014 - 30 August 2020. Data also presented for suicidal thoughts, but data were combined with “low mood”	Intentional drug overdose and self-harm	A marked reduction (p<0.001) in liaison psychiatry referrals for intentional drug overdose, self-harm and suicidal thoughts occurred after 23 March (lockdown). The proportion of referrals returned to pre-lockdown levels by May/June 2020.	Liaison team referral only (not all ED attendances) at a single hospital. Liaison psychiatry referral pathways may have changed as a result of COVID-19 No detailed demographic analysis of referrals as the paper focused on a wide range of mental and physical health presentations. Single area in England. Peer reviewed
Dragovic *et al.,* 2020	Australia	Western Australia (WA) North Metropolitan Health Services EDs were extracted from the Emergency Department Data Collection database. These 3 EDs serve a population of approx. 800,000 persons. Attendances over the period January to May 2020 were compared to those that occurred over the same calendar month periods during 2019.	Suicide attempts/self- harm	7140 attendances (5522 persons) over the two study periods. Suicidal and self-harm presentation decreased by 26% to previous year	Attendances at three hospitals but WA has low population density and went into stringent lockdown early - hence findings may not be generalisable to other Australian states or other countries; routinely collected healthcare data are large and complete, but they lack rich contextual detail. Peer reviewed
Gonçalves-Pinho *et al.,* 2021	Portugal l	People attending a Psychiatric Emergency Department in a tertiary hospital in North Portugal serving a population of approximately 3 million people. Attendance between March 19th and May 2nd 2020 (when "emergency state" / restriction of movement existed in Portugal in response to COVID-19) compared with same dates in 2019	“Suicide and self- inflicted injury presentations” to psychiatric ED	Between March 19 ^th^ and May 2 ^nd^ 2020, a significant reduction was identified in presentations of “suicide and intentional self-inflicted injury” to a metropolitan psychiatric ED, compared to the same period in 2019: N=36 v 81, a 55.6% reduction.	Based on attendances at a single hospital. Unclear if codes include people with suicidal thoughts as well as acts. Peer reviewed
Hernández-Calle *et al.,* 2020	Spain	Electronic health records examined at a major general hospital in Madrid, Spain: November 2018 to April 2020.	Suicidal thoughts	During March-April 2020, significantly fewer psychiatric emergency department visits due to suicidal ideation were reported compared to the same period in 2019.	Data only shown in a graph. Single centre study - findings may have limited generalisability. Peer reviewed
Hewson *et al.,* 2020	UK	31 prisons in UK Internal reports from Safer Custody Units in 31 prisons where healthcare is provided by CareUK (Russell Green, personal communication)	Suicide attempts/self- harm	After lockdown there were fewer implementations of Assessment, Care in Custody and Teamwork (ACCT) processes; to initiate care- plans for prisoners considered at risk of self-harm or suicide. Across the 31 prisons, there were 1079 ACCTs implemented in February 2020 compared to 828 in April 2020, a fall of just under 25%. Analysis of data for 8 prisons indicated that there were falls in incidents of self-harm, decreasing by a third from 324 in February 2020 to 214 in April 2020.	No gender breakdown (female prisoners in the UK generally have much higher rates of self-harm than male prisoners) Unclear the basis of the selection of the 8 prisons with self-harm data Peer reviewed editorial
Jacob *et al.,* 2020	Australia	Single trauma centre in Australia, serving a population of 1.5 million. Compared mean number of trauma admissions during March and April during years 2016 to 2020	Self-harm	During March and April 2020 a significant decrease in total number of trauma- related admissions was observed, but no significant difference in admissions following self-harm was seen.	Mean no. of admissions examined before and during the Covid-19 public health emergency. Findings from a single centre may not be generalisable. The study was evidently under-powered for examination of mean monthly self- harm admissions. Peer reviewed
Karakasi *et al.,* 2020a	Greece	Records of psychiatric emergency cases presenting at the psychiatric emergency department of AHEPA University General Hospital of Thessaloniki during the following equal time intervals: 1 March to 15-May 2019, 15November 2019 to 31 January 2020, 1 March to 15 May 2020.	Suicide attempts/self- harm	During the restrictive measures in Greece (March – May 2020), the number of hospital presenting emergency psychiatric incidents fell by half (p < 0.01). The number of suicide attempts was higher in March- May 2020 (n=7) compared to the same period in 2019 (n= 5) and Nov 2019-Jan 2020 (n=4)	Data from a single hospital Small numbers Letter. Uncertain if peer-reviewed
Lersch, 2020	USA	Emergency calls (911) to Detroit Police Department for services between 26th Feb (first reported case COVID in city) and 27th April 2020. Comparison with 2017–2019 and also number of COVID-19 cases in the city.	Suicide threats and suicides in progress	In the time period of interest during the pandemic in 2020, the number of 911 calls for mental health issues was the lowest of the 4 years (2017–2020), declining by 16% from 2019 to 2020. However, the number of calls for suicide threats declined in 2020, while the number of calls for suicides in progress remained relatively stable over the 4-year period. No significant correlations between daily number of COVID-19 cases in the city and the number of calls from mentally ill persons, but as the number of COVID-19 cases increased there was a decline in calls for suicides in progress, but a significant inverse correlation between numbers of COVID-19 cases and threats of suicide calls (Pearson’s r=0.394) and a similar but non-significant relationship with calls for suicides in progress. In local area analysis, “some of the ‘hotspots’ for suicide threats were in areas of higher rates of COVID-19 cases”.	Interesting analysis by numbers of COVID-19 cases, including by locality. Single city. Data are early and may not be complete for COVID-19 cases. Unclear if peer-reviewed
McAndrew *et al.,* 2020	Ireland	Electronic health records for the emergency department (ED) of a large teaching hospital in Dublin were examined during the first 8 weeks of the Covid-19 emergency (from 16th March to 10th May 2020). Comparative data for 2018 and 2019 were also examined.	Suicide attempts/self- harm, Suicidal thoughts	A 21% reduction in the frequency of psychiatric emergency presentations was observed, although the proportion of presentations with suicidal ideation or self-harm as factors remained unchanged. The observed reduction was largely due to a reduce attendance frequency during 'normal' hours.	Electronic health record studies are not prone to selection or self-report information biases. Further research examining patterns of emergency psychiatry presentations during COVID-19 could identify risky / vulnerable groups of people who have not been seeking help during a crisis. Similar studies from other countries and with extended follow-up periods are needed to build up a comprehensive picture of these temporal patterns. Peer reviewed
McIntyre *et al.,* 2020	Ireland	Self-harm referrals to Liaison Psychiatry team in a single tertiary care hospital in Gallway Ireland. Contrast 1 March 2020–31 May 2020 with the same period in 2017–2019	Self-harm presentations to a general hospital.	Between March-April 2020, a significantly lower proportion of self-harm presentations (-35%) to the hospital was reported, compared to the same period for 2017–2019. At the end of May, similar proportions of self-harm presentations were reported compared to previous years.	Single hospital study. Incidence based on referrals to liaison psychiatry - may under-estimate total hospital presenting cases. Liaison psychiatry referral pathways may have changed as a result of COVID-19. Peer reviewed
Olding *et al.,* 2021	England, UK	Trauma patients with penetrating injuries who were treated at King's College Hospital in London, 23rd March to 29th April 2020 compared to the same period in 2018 and 2019.	Self-harm (self- inflicted injuries	Whilst the incidence of all types of penetrating trauma appeared to have fallen by 35% during the early lockdown period), the number of self-harm episodes increased from n=1 in 2018 to 5 in 2019 and 8 in 2020	Small, single site study. Crude analytical approach. Number of self- harm cases too small to draw any strong conclusions Peer reviewed
Pignon *et al*., 2020a	France	Emergency psychiatric consultations from three psychiatric emergency centres from first four weeks of lockdown (started March 17th 2020) and corresponding weeks 2019	Suicide attempts	During the four first weeks of lockdown, 553 emergency psychiatric consultations were carried out, less than half (45.2%) of the corresponding weeks in 2019 (1224 consultations). Total suicide attempts decreased in 2020 to 42.6% of those in 2019.	Peer reviewed publication now published, Pignon *et al*., 2020b
Rajput *et al.,* 2020	England, UK	Trauma admissions to a single level 1 trauma centre in Liverpool using data from a trauma research network database. Compared three 7-week periods: (1) Lockdown: 23 March 2020–10 May 2020) (2) Pre-lockdown: 7 weeks prior to lockdown (27 January 2020–15 March 2020) (3) Pre-lockdown 2019: 7 week equivalent period in 2019 (25 March 2019–12 May 2019)	Suicide attempts/self- harm	Total trauma centre attendances fell during lockdown: 2019: n=194; 7 weeks pre lockdown 2020 n=173; during lockdown n=121 Equivalent numbers for self-harm were: 20 (2019); 24 (pre-lockdown 2020); 14 (lockdown 2020): i.e. 30% fall vs 2019.	Small sample size; no assessment of any change in socio-demographic characteristics of self-harm; possible changes due to service re-configurations in response to COVID. Peer reviewed
Rhodes *et al.,* 2020	USA	Trauma registry data of attendees at a Level 1 trauma centre in S Carolina, USA Jan 1-May 1 2019 compared to Jan 1 - May 1 2020 (lockdown April 8 ^th^ – May 1 ^st^ 2020).	Suicide attempts and self-harm, including specific methods	Some evidence of rise in suicide attempts: 2019: 6 (0.6% of all presentations); 2020: 11 (1.4%) (p=0.079), including ‘self- harm by jumping’: 2019: 0 (0%); 2020: 5 (0.6%); p=0.011). No change in other ‘self-harm’ presentations: gun: 2019: 4 (0.4%); 2020: 4 (0.5%) (p=0.716); knife: 2019: 2 (0.2%); 2020: 1 (0.1%) (p=0.719), nor in acts of ‘Undetermined intent’: 2019: 18 (1.8%); 2020: 6 (0.8%) (p=0.064).	Most of the period studied (15 of the 18 weeks) in 2020 preceded lockdown. Small numbers and no specific data on suicide attempts during the post-lockdown period. The statistical comparison of suicide/SH episodes compared these episodes as a % of total attendances, rather than changes in absolute numbers. Peer reviewed
Sade *et al.,* 2020	Israel	Pregnant women admitted to high risk pregnancy units between 19 March 2020 and 26 May 2020 (the strict isolation period of the pandemic) (n=90) compared to those hospitalised to these units between November 2016 and April 2017 (n=279)	Suicidal thoughts assessed using the Edinburgh postnatal depression scale (EPDS)	Prevalence of suicidal thoughts was similar pre (5.0%) vs during (8.6%) pandemic (p = 0.221). OR in multivariable logistic regression model, controlling for maternal age, adjusted OR 1.8, 95% CI 0.71–4.85, p = 0.203.	Admission criteria may have changed post pandemic (although admissions per month similar ~ 45/month Relatively small sample Select sample - pregnant women - generalisability to wider population uncertain. Pre-pandemic data collected in Nov 2016-April 2017 - 3 years previously - no account of any secular trends (also seasonal difference in collection period). Peer reviewed
Sheridan *et al.,* 2021	USA	Emergency department visits for mental health issues to a single tertiary care pediatric hospital in Portland, Oregon April 1 2019 up to 29 April 2020	Suicidal patients	Department dealt with 14108 patients in 2019. 16 suicidal patients seen in April 2020 vs. 46 in April 2020 (a 65% fall)	Before / after lock down comparison, time trend analysis Used routinely available data Data on suicidal patients only specified for 1 month. One tertiary centre so not generalisable. Peer-reviewed
Smalley *et al.,* 2021	USA	Attendees with suicidal thoughts and alcohol issues across 20 diverse EDs in a large Midwest integrated healthcare system with >750,000 ED visits annually. All behavioural health (BH) visits were collected for 1-month (March 25 ^th^ to April 24, 2020) following “stay at home” orders (lockdown). ICD-10 codes were used to identify visits associated with suicidal thoughts. The same parameters were used to collect data for the same time period for 2019.	Suicidal thoughts ICD coded by hospital staff	Comparing 2020 with the same period in 2019, there was 44.4% decrease in overall ED visits and 28.0% decrease in BH visits. Attendances of individuals with suicidal thoughts decreased by 60.6% in 2020 (n=451) vs. 2019 (n=1144). As a percentage of all ED attendances, suicidal thoughts attendances decreased from 2.03% in 2019 to 1.44% in 2020.	Alternative avenues for help- seeking not included. But highlights importance of improving access for vulnerable populations during a pandemic. Included only one month in 2019 and one in 2020. Letter to editor, probably not peer reviewed
Titov *et al.,* 2020	Australa	Callers / website visits to "Mindspot" - national digital MH service in Australia. Compared caller volume and characteristics 1- 28 Sept 2019 (n=1650) vs. 19 March - 15 April 2020 (n=1668)	Suicidal thoughts question from PHQ-9	No change in prevalence of: a) suicidal thoughts (30.6% in September 2019 vs. 27.5% in March-April 2020; p=0.08), or b) suicidal intentions or plans (3.7% v 2.9% post p=0.27)	Clinical / helpline sample - not population based. Possible seasonal differences- September contacts vs. March-April Evidence of increased contact volume to a digital service. Peer reviewed
Walker *et al.,* 2020	USA	ED attendances (adult and pediatric) from an integrated multiple hospital / ED system. n=18 EDs across several states. Diagnoses via electronic health records. Pandemic period (17 March 2020 to 21 April 2020) compared to same period in 2019 (17 March 2019 to 21 April 2019) and 36 day pre- pandemic period in 2020 (9 Feb 2020 to 16 March 2020)	Suicide attempts/self- harm	Total ED attendances fell by around 50% during the period of "the broad institution of distancing measures in response to the COVID-19 pandemic". Likewise, total ED attendances with "suicide" diagnosis fell by around one third during pandemic period: 17 March 2020 to 21 April 2020: n=36 (0.2% of total attendances) vs. 17 March 2019 to 21 April 2019: n=59 (0.2% total attendances) 9 Feb 2020 to 16 March vs. 2020: n=64 (0.2% total)	Hospital presentations only Only includes first 36 days of distancing measures. Peer reviewed

**Table 6.** Summary of studies assessing suicide rates.

Authors	Geography	Data used	Conclusions	Comments/Limitations
Acharya *et al.*, 2020	Nepal	News reports of police data on suicides in Nepal 2019–2020	April 2020-mid July 2020: 1233 suicide deaths Feb-March 2020: 414 suicide deaths. Report 414 suicides /month pre-lockdown vs. 559/month after lockdown (a 35% rise)	Paper uses newspaper reporting of police suicide statistics as primary source of data, so may not be reliable. Letter to editor, probably not peer reviewed
Calderon-Anyosa & Kaufman, 2020	Peru	Suicide deaths reported by the Peruvian National Death Information System between 1st January 2017 and 28th June 2020.	Interrupted Time Series (ITS) analysis. Suicide deaths fell sharply in males and females from the start of the lockdown period (March 16 2020)	Authors used appropriate time series methods Only 80% of all deaths are registered by the Peruvian National Death Information System. It is unclear whether cause of death assignment is time-lagged in Peru. Pre-print. Not peer reviewed
Isumi *et al.*, 2020	Japan	Suicide statistics published by the Ministry of Health, Labor and Welfare for children (aged <20 years) Jan 2018-May 2020	Investigated the impact of school closures (March– May 2020) by comparing these months with the same period in 2018 and 2019 using Poisson regression. In 2018 and 2019, suicide rates tend to increase from March to May; however, suicide rates from March to May in 2020 appeared to decrease slightly. Compared to March to May 2018 and 2019, no strong evidence of an increase in suicide rates during these months in 2020 (the school closure period): Incidence rate ratio =1.15, (95% CI 0.81 to 1.64).	Analysis did not account for possible underlying temporal trends in suicide using time-series approaches. Publicly available national statistics. Possibly too short a timespan to assess impact on child suicides. Suicides among children and adolescents reportedly peak at the beginning of school semesters in Japan, suicide rates may have increased when school restarted in June 2020. Peer reviewed
Pokhrel *et al.*, 2021	Nepal	News reports of police data on suicides in Nepal 2019–2020	Report a 25% rise in suicide deaths in the lockdown period (after mid-March 2020) compared to pre- lockdown. 1647 suicides between mid-March 2020 and 27 June 2020.	Data derived from newspaper reporting of police suicide statistics as primary source of data, so may not be reliable. Letter, may not have been peer reviewed.
Poudel *et al.*, 2020;	Nepal	News reports of police data on suicides in Nepal 2019–2020	Report a 20% rise in suicide deaths In the first month of lockdown (from 24 March) (487 suicides vs. 410 in mid-February to mid-March 2020). Since the start of lockdown up to 6 June, there were 1,227 suicides (16.5/day) compared to 5,785 (15.8/day) in the same period in 2019	Data derived from newspaper reporting of police suicide statistics as primary source of data, so may not be reliable. Peer reviewed
Sakelliadis *et al.*, 2020	Greece	Autopsies carried out at the University of Athens March 17th–April 15th 2019 vs. March 17th–April 15th 2020 (the first month of lockdown	Total autopsies - 125 in 2019 ; 105 in 2020. The number of suicides and undetermined deaths were similar in 2019 vs. 2020: suicides: 6 (2019) vs 4 (2020); deaths of undetermined intent 4 (2019) vs. 5 (2020).	Small numbers of deaths. Possible changes / delays in death investigation during COVID-19? Peer reviewed
Singh *et al.*, 2020	Nepal	News reports of police data on suicides in Nepal 2019–2020	Suicide deaths rose by 20% in Nepal during period of Covid-19. Within the first 74 days of lockdown on average 16.5 people per day died by suicide vs. 15.8 per day in 2019	Paper uses newspaper reporting of police suicide statistics as primary source of data, so may not be reliable. Letter, may not have been peer reviewed.
Tanaka & Okamoto, 2020	Japan	Suicide statistics (all ages) published by the Ministry of Health, Labor and Welfare. July 2016 – June 2020	Compared Use Feb-Jun 2020 (COVID period) vs. and Feb-Jun 2016–19 (pre-COVID). Suicides fell by 13.5% (95% CI -17.5 to -9.5%) in the COVID period. Decline is greatest in males and in adults compared to children (<20 years) and older people (>70 years). No evidence of an adverse effect on students during school closure (rates fell).	Publicly available national statistics. The authors cite the Japanese government’s "generous subsidies, reduced working hours, and fewer school sessions" as possible explanations for lack of adverse effect. Pre-print. Not peer reviewed.
Ueda *et al.*, 2021	Japan	Suicide statistics (all ages) published by the Ministry of Health, Labor and Welfare. Jan 2017-August 2020	During the state of emergency (April-May 2020), suicides declined by 20%. By August suicide numbers were 7.7% higher than the average for August 2016–19. The largest rises were in females (mean of 532 suicides in August 2017–19 vs. 651 in 2020). Similar trajectories in all age groups, but the largest rise was in those aged <40 years (63% higher in 2020 vs 2017–19). Groups of greatest concern: students (47% rise in university student suicides August 2020 vs August 2017–19) and housekeepers.	Authors speculate greater rise in women could be because they largely worked in the sectors most affected by pandemic related closure (retail and travel) The analysis did not account for possible underlying temporal trends in suicide using time-series approaches. the authors compare the percentage change in a single month in 2020 vs figures for the same month in 2017–19. Pre-print. Not peer reviewed

### Study populations

Sample sizes ranged from two individuals in a number of case series ( Kapilan, 2020; Mamun *et al*., 2020b; Pirnia *et al*., 2020; Sahoo *et al*., 2020) to 60 million Twitter posts ( Saha *et al*., 2020). Most studies included both male and female participants, except the studies reported by Wu *et al*. (2020a) and Sade *et al*. (2020) which were conducted in pregnant women.

### Outcomes

Seven of the 24 case series (described in 25 papers) focused on a mix of outcomes including suicide attempts (n=2), suicide deaths (n=14) and suicidal thoughts (n=1). Of the 15 cross-sectional surveys five assessed suicidal thoughts alone, others collected data on various combinations of suicidal/self-harming behaviour or thoughts. A range of validated questionnairres were used to assess suicidal thoughts ( Table 2). Five surveys used the single item from PHQ-9 ‘Have you had thoughts that you would be better off dead or of hurting yourself in some way’ over the last 2 weeks. Wang *et al*. (2020b) assessed responses to this question in a symptom network analysis.

### Summary of study findings: Case series

We identified 24 case series of suicide attempts and suicide deaths ( Table 1). Fourteen (58.3%) of these used news reports as their data source ( Bhuiyan *et al*., 2020; Boshra *et al*., 2020; Dsouza *et al*., 2020; Griffiths & Mamun, 2020; Kapilan, 2020; Kar *et al*., 2020; Mamun & Ullah, 2020; Mamun *et al*., 2020a; Mamun *et al*., 2020b; Rahman *et al*., 2020; Rajkumar, 2020; Shiob *et al*., 2020; Syed *et al*., 2020; Thakur & Jain, 2020) and are unlikely to be representative of general population suicide rates. Several overlap in terms of the information used, such as two letters to the editor about celebrity suicides in India ( Kar *et al*., 2020; Mamun *et al*., 2020b), and many lack detailed information about the range of contributing factors. Whilst most case series focused on suicdes in the general population, some focussed on specific groups, such as psychiatric patients (e.g. Jolly *et al*., 2020), healthcare professions (e.g. Kapilan *et al*., 2020), patients with COVID-19 (e.g. Nalleballe *et al*., 2020), couple suicides ( Griffiths & Manun, 2020) and alcohol-related deaths (e.g. Ahmed *et al*., 2020).

Many reasons for COVID-19 related suicide or suicide attempts were suggested in the case series with conclusions often derived from a journalist’s report of the death. Contributory factors reported included fear of contracting the disease or of passing it on to others, reactive psychoses, financial or economic issues, loneliness and isolation due to quarantine, stress among health professionals, the uncertainty around when the pandemic would end, misinterpretation of fever as COVID-19, contracting COVID-19, an inability for migrants to return home, frustration and the stigma of a (possibly perceived) positive result, which resulted in harassment or victimisation by others in the community. In the largest case series from India (n=72 suicide deaths), Dsouza *et al*. (2020) reported that the most commonly occurring antecedents to suicide were fear of infection (n=21) and financial crisis (n=19). Two studies reported specifically on the consequences of alcohol withdrawal due to lockdowns ( Ahmed *et al*., 2020; Syed *et al*., 2020).

In the USA, four case reports described stressors for adolescents which include inability to see friends, arguments with parents, unresolvable misunderstandings over social media, academic stress, and feelings of isolation ( Jolly *et al*., 2020). In a case series of adults across three hospitals in Doha, Qatar, three patients (out of 50 patients with COVID-19 receiving a psychiatric diagnosis) self-harmed as a reaction to the pandemic ( Iqbal *et al*., 2020). A study of TriNetX records of people with COVID-19 (n=40,469) found that 0.2% (62 individuals) had suicidal thoughts recorded, although clinicians may not have systematically asked about suicidality ( Nalleballe *et al*., 2020). 

### Summary of study findings: Cross-sectional surveys and cohort study

There were 13 articles describing cross-sectional surveys / cohort studies of two or more waves or one wave surveys where comparisons were explicitly made with appropriate pre-pandemic measures; or included comparative data between COVID-19 positive individuals and unaffected comparison individuals ( Table 2). Six studies present repeat survey data, with measures recorded during, as well as before, the pandemic ( Hamm *et al*., 2020; Hamza *et al*., 2021; Raifman *et al*., 2020; Winkler *et al*., 2020; Wu *et al*., 2020b; Zhang *et al*., 2020). Raifman *et al*. (2020) compared two nationally representative samples of US adults (one from 2017/18 and one from 2020 during the COVID-19 pandemic) using different survey methodologies. They found that suicidal ideation had increased more than fourfold in low-income households, particularly in those with difficulty paying rent, job loss and loneliness. Similarly, Winkler *et al*. (2020) reported on a repeated, robustly-sampled, nationally representative survey in the Czech Republic using baseline data from 2017 and found that suicide risk, as measured by the Mini International Neuropsychiatric Interview, increased from 3.9% in November 2017 to 11.9% in May 2020. Both Raifman *et al*. (2020) and Winkler *et al*. (2020) used somewhat different data collection approaches before and during the pandemic. Two other studies from China ( Wu *et al*., 2020b; Zhang *et al*., 2020) reported increases in relevant outcomes during the pandemic compared with before. The cohort study by Zhang *et al*. (2020) reported increases seen in nonsuicidal self-injury (NSSI), suicidal thoughts, suicidal plans, and suicide attempts in primary and secondary school children pre- compared with post-pandemic. Neither Hamm *et al*., 2020 (trial participants with depression aged >60years) nor Hamza *et al*., 2021 (students) found clear evidence of increased risk of suicidal ideation (older adults) or NSSI (students) during the pandemic.

Three additional articles, other than Raifman *et al*., 2020 and Winkler *et al*., 2020, reported cross-sectional surveys in the general population. Two of these used web based recruitment ( Iob *et al*., 2020; Sueki & Ueda, 2020) with non-probability quota sampling or weighting, and one ( Wang *et al*., 2020b) used a Chinese online platform providing functions similar to Qualtrics. Participants were COVID-19 patients in three studies ( Wang *et al*., 2020a; Wu *et al*., 2020a; Zhao *et al*., 2020). Wang *et al*. (2020a) and Zhao *et al*. (2020) both reported higher levels of suicide-related outcomes in COVID-19 patients than general population (compared with the general population recruited through social media or from literature reports). In a general population sample that included people who reported having been diagnosed with COVID-19, Iob *et al*. (2020) found suicide/self-harm thoughts were more common in those with a COVID-19 diagnosis than in those not affected (33% vs 17%); likewise for suicide attempts (14% vs. 5%). Two surveys were conducted in university student populations ( Debowska *et al*., 2020; Hamza *et al.*, 2020) from 11 universities, with predominantly female respondents. No statistical evidence of a rise in suicidal thoughts or self-injury was found over a number of waves of data collection. Surveys were targeted at specific populations in a further three studies ( Table 2): depressed patients ( Hamm *et al*., 2020); pregnant women ( Wu *et al*., 2020b); and school children ( Zhang *et al*., 2020). 

### Summary of study findings: Social media platform posts

Two studies ( Table 3) assessed posts on social media platforms, looking at Reddit ( Low *et al*., 2020; 50% USA users) and Twitter in the USA ( Saha *et al*., 2020). Both studies show marked increases in the proportion of postings related to suicidal thoughts and behaviours, and Low *et al*’s analysis of Reddit data identified a new cluster of posts about self-harm during the pandemic.

### Summary of study findings: Modelling studies

We identified six studies ( Table 4) that have used modelling approaches to forecast the potential impact of the pandemic on future suicide rates ( Bhatia, 2020a; Bhatia, 2020b; Kawohl & Nordt, 2020; McIntyre & Lee, 2020a; McIntyre & Lee, 2020b; Moser *et al*., 2020). Three estimated the impact of the pandemic on suicide in the USA ( Bhatia, 2020a; Bhatia, 2020b; McIntyre & Lee (2020a), while others assessed the impact on suicide in Canada ( McIntyre & Lee, 2020b), Switzerland ( Moser *et al*., 2020) and worldwide ( Kawohl & Nordt, 2020).

The models suggest between a 1% rise ( Kawohl & Nordt, 2020, globally) and a 145% rise ( Moser *et al*., 2020, in Switzerland) in suicide deaths. Each was based on different assumptions, but the models largely focused on the well-characterised impact on suicide rates of periods of economic recession and rises in unemployment ( Chang *et al*., 2013; Stuckler *et al*., 2009). Unemployment rates are predicted to rise as a result of a post-pandemic recession, due to measures to control the spread of the virus on the wider economy and loss of work as many businesses have been forced to shut down.

Only one study modelled the effects of physical distancing measures on suicide rates ( Moser *et al*., 2020); it did this by using suicide rates in prisoners in group or single cells as a model for lockdown in a group or in isolation. The prison population is exposed to multiple other risk factors for suicide (e.g. increased prevalence of mental illness, substance misuse and low socioeconomic position) ( Humber *et al*., 2011; Rivlin *et al*., 2010), and this, coupled with the distinct differences between prison incarceration and the adoption of home quarantine procedures during the pandemic, means this model is likely to overestimate the potential impact of physical distancing measures on suicide risk in the general population.

### Summary of studies’ findings: Service utilisation studies

We identified 20 service utilisation studies. Four of these addressed the impact of COVID-19 on suicidal thoughts only, thirteen included suicide attempts and/or self-harm, one suicidal thoughts, attempts and self-harm ( McAndrew *et al*., 2020), one suicide threats and suicides in progress ( Lersch, 2020), while in one the precise nature of the suicidal outcome was unclear ( Sheridan *et al*., 2021) ( Table 5). Most studies were conducted in the US (5) and the UK (4), three in Australia, two in Ireland and one study in each of the following countries: France, Greece, Israel, Italy, Portugal, and Spain. 

Across the studies focusing on suicidal thoughts, the methodologies varied from studies of presentations to health/mental health services to callers/visits to a website, with wide-ranging sample sizes, from 1668 ( Titov *et al*., 2020) to 90 ( Sade *et al*., 2020); the latter including a specific sample of pregnant women. The studies showed either a reduction ( Chen *et al*., 2020; Hernandez-Calle *et al*., 2020; Smalley *et al*., 2021) or no change ( Sade *et al*., 2020; Titov *et al*., 2020) in presentations to health/mental health services or self-reported suicidal thoughts, with the majority making comparisons to the same time in 2019. The eleven studies examining the impact of COVID-19 on self-harm/suicide attempts used a variety of methodologies, including accessing data from health/mental health services, trauma registries, community-based services, emergency call services and the prison service. Where reported, the sample sizes ranged from 18,646 ( Walker *et al*., 2020) to 30 ( Olding *et al*., 2021). Eight studies reported a decrease in self-harm/suicide attempts during the first months of the COVID-19 pandemic ( Capuzzi *et al*., 2020; Chen *et al*., 2020; Goncalves-Pinho *et al*., 2021; Hewson *et al*., 2020; McIntyre *et al*., 2020; Pignon *et al*., 2020; Rajput *et al*., 2020; Walker *et al*., 2020). In two of these studies – both with somewhat longer post lockdown follow-up periods of 3–5 months ( Chen *et al*., 2020; McIntyre *et al*., 2020) – presentations had returned to pre-lockdown levels by the end of follow-up. Three studies reported an increase in self-harm / suicide attempts (Karakasi *et al*., 2020; Olding *et al*., 2021; Rhodes *et al*., 2020).

Pignon *et al*. (2020) reported a 54.8% decrease in overall psychiatric emergency consultations and a 42.6% decrease in self-harm/suicide attempts during the first 4 weeks of the lockdown in France compared with the same period in 2019. Likewise, Gonçalves-Pinho *et al*. (2020) identified a 55.6% decrease in presentations of “suicide and intentional self-inflicted injury” to a metropolitan psychiatric emergency department in Portugal in the period 19 ^th^ March to 2 ^nd^ May between 2019 and 2020. McIntyre *et al*. (2020) reported a 35% reduction in self-harm presentations to a general hospital in March-April 2020 in Ireland compared with the same period in 2017–2019; however presentations returned to pre-pandemic levels by the end of May. Another study in Ireland ( Mcandrew *et al*., 2020) also reported a reduction in psychiatric emergency presentations to the emergency department but no change in the proportion of presentations with suicidal thoughts or self-harm. In a study conducted by Hewson *et al*. (2020) in 31 prisons in the UK between February and April 2020, self-harm incidents decreased by one third between February and April 2020.

In contrast, whilst Olding *et al*. (2021) reported a reduction in the incidence of all types of penetrating trauma presenting to a UK hospital during the early period of lockdown, the number of self-harm presentations increased slightly (albeit on the basis of very low event counts). A similar pattern was identified by Karakasi *et al*. (2020a) in Greece, where between March and May 2020 a significant reduction was observed in individuals presenting as emergencies at hospital for psychiatric examination (the number of presentations for suicide attempts was 7 compared with 5 in the same period in 2019). Capuzzi *et al*. (2020) reported a rise in self-harm / suicide attempts as a proportion of total emergency department presentations in Italy, but this rise in the proportion of cases was in the context of falls in the absolute numbers of cases, set against reductions in total emergency department attendances.

A study of emergency police calls in Detroit, USA, (Lersch *et al*., 2020) showed that the number of general mental health calls declined after the onset of the pandemic in that city, while calls relating to suicides in progress remained relatively stable over the 4 year period. Calls involving suicide threats declined inversely to the increase in COVID-19 infections, although the authors noted some ‘hotspots’ within the city for both infection rates and suicide threats. A study of 31 prisons in the UK found that after lockdown there were fewer implementations of Assessment, Care in Custody and Teamwork (ACCT) processes to initiate care- plans for prisoners considered at risk of self-harm or suicide ( Hewson *et al*., 2020).

### Summary of study findings: impact of COVID-19 on suicide rates

Nine reports, based on data from four countries – Greece, Japan, Nepal and Peru – describe changes in suicide rates in relation to the onset of COVID-19 and national lockdowns. A challenge with interpreting all the reports is the uncertainty over the extent to which official recording of suicides may have been affected by disruptions in death investigation and reporting due to COVID-19, although this is more likely to lead to under-estimation than over-estimation of suicide rates. Only one of the studies ( Calderon-Anyosa & Kaufman, 2020) used appropriate time series to take account of underlying temporal trends in suicide when comparing the COVID-19 period with earlier years/months.

The four reports from Nepal ( Acharya *et al*., 2020; Pokhrel *et al*., 2021; Poudel & Sedhai, 2020; Singh *et al*., 2020) were all based on news reports of police data on suicides, rather than on data obtained directly from Nepalese authorities and did not describe the strengths and weakness of the police data. They report between a 20% (Poudel & Sedhai, 2020) and 35% ( Acharya *et al*., 2020) rise in suicide in the first 3 months after lockdown compared with either preceding months or a similar period the previous year. These are marked rises, but without longer time series data it is not possible to determine the extent to which these were COVID-19 related or a possible continuation of pre-existing adverse trends. Three reports, based on Japan’s timely national suicide statistics, describe recent trends in Japanese suicide rates ( Tanaka & Okamoto, 2020 pre-print, Tanaka & Okamoto, 2021, final version; Isumi *et al*., 2020; Ueda *et al*., 2021). The most recent of these, using data up to October 2020, indicate that 14% falls in Japanese suicides in the early months of the pandemic (Feb-June 2020), were reversed during the second outbreak (July to October, 2020) increasing by 16% ( Tanaka & Okamoto, 2021). Increases in suicide rates were higher in females (especially housewives) and children and adolescents. Similarly compared with August in 2017–19, figures for August 2020 were increased by 7.7%, with rises particularly in females and people aged <40 years ( Ueda *et al*., 2021). An early report (data up to May 2020) provided some reassurance about the impact of public health measures/school closures on suicide rates in children (<20 years) in Japan ( Isumi *et al*., 2020). However, more recent data ( Ueda *et al*., 2021) flags a concerning rise amongst students and young (<40 years) people, particularly females. The numbers of deaths in the autopsy study from Athens ( Sakelliadis *et al*., 2020) is too small to reach any conclusion about the impact on suicide in Greece. Calderon-Anyosa’s (2020) study of suicide in Peru is reassuring, though details of potential impacts of COVID-19 on death registration in Peru are not provided.

### Other studies

The three other studies investigated various risk groups, using case control and mixed methods approaches. Son *et al*. (2020) interviewed students from a single US university about the impacts of the pandemic on their mental health; some students described suicidal thoughts and the challenges they faced, one linked suicidal thoughts to being confined at home with their family and another to study-related difficulties. Cai *et al*. (2020) compared suicidal thoughts in Chinese medical workers dealing with COVID-19 patients and those not in contact with such patients. They found no evidence of increased levels of suicidal thoughts amongst those in contact with COVID patients. Lastly, Evans *et al*. (2020) studied the pandemic-related stresses felt by Australian families in free text responses to a questionnaire. One respondent, a father with three children described the extreme financial distress they faced with “our three businesses closing, we are eligible for none of the government support due to a tax debt and are looking at bankruptcy and selling our home as the only option. Both of us have had thoughts of suicide" (Quote from father of 3 children). ( Evans *et al*., 2020)

## Discussion

Seventy-eight articles were included in this review, 49 more than in our review of studies published up to 7 ^th^ June 2020. All were based on observational studies. The majority of studies were case series or service utilisation studies from across the world. No studies were based on populations from sub-saharan Africa. Almost half of the articles did not appear to have been peer-reviewed, consisting mainly of pre-prints published before peer review, or research letters that may not have been peer-reviewed. In contrast to the last update ( John *et al*., 2020c) in which no studies reported on the change in incidence of suicide or suicidal behaviour after the onset of the pandemic compared with beforehand, we identified nine papers in this update, presenting data on studies from four countries which investigated the impact of COVID-19 on suicide rates. To date, the highest quality data come from Japan which utilises suicide records covering the entire population; these data indicate that the impact of COVID-19 on suicides rates may change over time and have varying effects on different sections of the population. Analysis of data from Peru used appropriate analytic techniques and reported a fall in suicides following the onset of the pandemic during the months March to September ( Calderon-Anyosa & Kaufman, 2020). Methodological limitations and the availability of data for only four countries limit our ability to assess the early impact of COVID-19 on suicide rates in this update.

Evidence published following our cut-off date for inclusion in this iteration of the review indicates there was no rise in suicide rates in the early months of the pandemic in high income countries ( John *et al*., 2020a). Since our 19 ^th^ October search, a further 13 studies analysing suicide trends in ten countries or regions within countries (Australia, Austria, Germany, Greece, Japan; Korea, Norway, Sweden, Thailand and the USA) have been published ( Ando & Furuichi, 2020; Bray *et al*., 2021: Deisenhammer & Kemmler, 2021; Faust *et al*., 2020; Karakasi *et al*., 2021; Ketphan *et al*., 2020; Kim, 2021; Leske *et al*., 2021; Mitchell & Li, 2021; Qin & Mehlum, 2021; Radeloff *et al*., 2020 and Radeloff *et al*., 2021; Rück *et al*., 2020; Vandoros *et al*., 2020). Four of these use appropriate time-series modelling approaches to control for underlying trends ( Leske *et al*., 2021, Australia; Faust *et al*., 2020, USA; Vandoros *et al*., 2020, Greece; Ando *et al*., 2020, Japan) – these report either no change or a fall in suicide deaths in the early months of the pandemic, although in keeping with Tanaka & Okamoto (2020); Tanaka & Okamoto (2021) and Ueda *et al*.’s (2021) analysis for Japan, Ando *et al*. (2020) report a rise in suicides in Japan since July associated with increased unemployment . In keeping with concerns from Nepal, data from Thailand’s Department of Mental Health indicate suicide numbers have risen during the pandemic ( Ketphan *et al*., 2020). Data from Connecticut, USA on suicides during the 10 weeks of stringent lockdown measures in the state indicate that whilst suicide rates fell during this period, the proportion of suicides amongst minority ethnic groups rose, highlighting the possibility that the pandemic may be having a disproportionately greater adverse impact on minority groups ( Mitchell & Li., 2021). A concern supported by a recent analysis from Maryland, USA. ( Bray *et al*., 2021).

The majority of the 13 included cross-sectional surveys were subject to methodological flaws in sampling methods and use of validated instruments. Nonetheless, there is evidence from at least three countries (China, Czech Republic and USA) of increases in suicidal/self-harm thoughts in the general population during the pandemic compared with pre-pandemic levels. Two robustly sampled general population, nationally representative cross-sectional surveys with pre pandemic baseline data from 2017/18 reported a three to four fold increase in suicide risk ( Winkler *et al*., 2020) and suicidal thoughts in low-income households ( Raifman *et al*., 2020), but differences in data collection approaches (i.e. face-to-face vs. on-line) may bias comparisons. Recent studies, with repeat measures of mental health outcomes since the start of the pandemic, also point to rising levels of suicidal thoughts during the pandemic ( O’Connor *et al*., 2020).

The review included 20 service utilisation studies (compared with only three in the previous update), the majority of which identified a drop in frequency of emergency department contacts for suicidal thoughts, behaviours and self-harm. An increase in contacts to a mental health digital platform was identified in one study ( Titov *et al*., 2020), but with no changes in contacts for suicidal thoughts. There have been several recently published service utilisation studies (Carr *et al*., 2020; Hawton *et al*., 2020a; Jollant *et al*., 2020) which reiterate and extend these findings. Jollant *et al*. (2020) report a 8.5% decrease in hospitalisation for self-harm, greater in females than males, in France in January to August 2020 compared with the same period in 2019. There was also an increase in use of some more lethal methods (firearms / jumping/ drowning) as well as a rise in in-hospital deaths and ITU admissions. Carr *et al*. (2020) report a 30% fall in consultations for self-harm in April to June 2020 in primary care and secondary care in the UK, the former a setting not explored in currently included studies. They highlight that the treatment gap for depression and anxiety was greater in working age adults, for practice populations in deprived areas, and for self-harm. A limitation of all studies based on hospital presentations is that they may not reflect community prevalence of suicidal thoughts and behaviours. This may be a particular issue if people were deterred from presenting to hospital because of fears of either over-burdening already stretched healthcare systems or of contracting the virus in these settings themselves. That said, those who present to services may be able to give some insight into whether COVID-19-related concerns are important. In one UK study, ‘stay-at-home’ related issues contributed to around half of cases, more so in males than females. The most frequent COVID-related factors were mental health issues, including new and worsening disorders, cessation, reduction or transformation of services (including absence of face-to-face support), isolation and loneliness, reduced contact with key individuals, disruption to normal routine, and entrapment ( Hawton *et al*., 2020b).

Modelling studies that aimed to predict the impact of the pandemic on national or global suicide rates produced widely differing estimates of the likely impact and most focused on predictions based on previous studies of the impact of changes in unemployment levels on suicide. These differences between model estimates were partly due to differences in modelling assumptions, which are themselves in turn associated with considerable uncertainty. Given the methodological limitations, the uncertainty of assumptions about how the economies of individual countries will be affected, as well as international differences in financial supports given to businesses and people out of work, these predictive exercises can at best only provide a guide as to where action and available suicide prevention strategies should be directed.

Studies of social media posts potentially provide another insight into the impact of the COVID-19 pandemic on suicide risk and have the potential to provide more-or-less real time assessments of changes in risk. The two studies we identified ( Low *et al*., 2020; Saha *et al*., 2020) reported heightened levels of suicide-related posting/suicidality. However, there are several limitations to this approach making these studies hard to interpret, including: self-selecting biases in respect of who contributes to these fora (and when); the unit of analysis being posts/tweets rather than individuals so multiple posts may be from the same individual; and the dissemination of misinformation; the demographic and clinical characteristics of the people making the posts are unknown; and whether comments reflect their own distress or more general concerns is uncertain.

It is also not clear whether mentions of suicide on social media posts map to actual rates of suicidal thoughts in the community and whether this changes in particular contexts and over time. The nature of the relationship (if any) between social media reports and behavioural change in the context of suicide needs to be better understood. Insights derived from such approaches may help deepen our understanding of the mental health challenges of the pandemic and how these may change over time. Future research could usefully try to segment the posts by individuals and sociodemographics to explore changes in sub-groups. Another potentially useful approach to assessing the impact on COVID-19 on population mental health and suicide risk is analysis of Google trends data ( Jacobsen *et al*., 2020; Knipe *et al*., 2020; Rana, 2020; Sinyor *et al*., 2020), but we excluded such studies from our review as we think that search data constitute an even weaker proxy for population mental health.

We identified 25 case series of suicide attempts and suicide deaths, 14 based on news stories in India, Bangladesh and Pakistan. Given the relatively low quality of case series in the hierarchy of evidence, often reflecting small numbers and selection bias, but more importantly the lack of comparator data, drawing any reliable inferences from these studies is inherently flawed. Furthermore, news reports report a non-representative sample of suicide deaths and often derive their information from bystanders and witnesses who are unlikely to know the full circumstances of the death ( Khan *et al*., 2009). However, in parts of the world without reliable suicide incidence data they may be the only source of information ( Khan & Hyder, 2006). Nevertheless, these studies highlight circumstances surrounding apparently COVID-19-related suicides and flag the potential importance of factors such as economic difficulties, fear of the disease, alcohol withdrawal and social isolation even in young people and children.

Only 14% (11/78) included studies specifically focussed on children and young people. An early report (data up to May 2020) provided some reassurance about the impact of public health measures/school closures on suicide rates in children (<20 years) in Japan ( Isumi *et al*., 2020). However, more recent data ( Tanaka & Okamoto, 2021; Ueda *et al*., 2021) flags a concerning rise amongst students and young ((<40 years) people, particularly females and children and adolescents during the second wave of the pandemic and school closure. Three were cross-sectional surveys with attendant methodological flaws. Two surveys were conducted in university student populations ( Debowska *et al*., 2020; Hamza *et al*., 2021) in 11 universities with predominantly female respondents. No statistical evidence of a rise in suicidal thoughts or self-injury was found over a number of waves of data collection. Wang *et al*’s (2020) network analysis of symptoms of anxiety and depression in young people highlighted an increasing connection between ‘too much worry’ and suicidal thoughts. It is challenging to assess how generalisable these findings from China are to other countries and other phases of the pandemic. If generalisable, it could point to some treatment targets that are more central to suicide risk, but this is not yet clear. Zhang *et al*’s (2020) cohort study reported pre-pandemic comparison data, with increases seen in NSSI, suicidal thoughts, suicidal plans and suicide attempts in primary and secondary school children post-pandemic. However the sampling frame was poorly reported so representativeness of the sample is challenging to assess. Only one of the service utilisation studies focussed on this age group ( Sheridan *et al.*, 2021) but this was based in a single tertiary centre; although another study of a broader age range included them ( Walker *et al*., 2020). There were two case series focussed on children and young people ( Jefsen *et al*., 2020b; Jolly *et al*., 2020). The stressors identified for adolescents included the inability to see friends, arguments with parents, unresolvable arguments via social media, academic stress and feelings of isolation ( Jolly *et al*., 2020).

Only three included studies focussed on frontline healthcare staff. Two were case series (Kapilan *et al*., 2020; Rahmen *et al*., 2020) based on news reports of six or eight nurses deaths (i.e. there is potential duplication of reports of the same deaths). Factors reported as associated with deaths included: fear they had become infected; positive test result; being in quarantine; fearful of becoming infected; and “ extreme stress and mental disturbance”. The third, a case control study, reported that the prevalence of suicidal thoughts was no higher in medical staff who were in direct contact with COVID-19 patients, compared to those who had no direct contact ( Cai *et al*., 2020).

### Strengths and limitations

The literature exploring COVID-19 and suicide deaths, suicidal behaviours, self-harm and suicidal thoughts is expanding rapidly. Since our last review end-date (i.e. between 7 ^th^ June 2020 to 19 ^th^ October 2020) we identified a further 4156 potentially eligible studies. While most of the published evidence that we identified in this update had important limitations there was a marked improvement in study quality compared with our last update. Importantly, a large volume of the literature remains not peer reviewed; some reports are pre-prints, so this may change, but a number are research letters. All included studies remain observational in design and thus potentially prone to multiple sources of bias (e.g., recall bias, selection bias, confounding).

A number of the studies included in this update used non-probability samples e.g. convenience samples of volunteers recruited via the Internet. Such studies tend to attract volunteers who have access to the internet, are already engaged in research or have an interest in the topic. When assessing suicidal thoughts and behaviours, those in most distress or with co-existing mental illness, as well as older people, may be less likely to participate. Therefore prevalence estimates and associations observed among healthy volunteers may not reflect associations that would be seen in representative samples ( Pierce *et al*., 2020). However, such study designs potentially provide potentially valuable information at the very early stages of a health crisis, where the timeliness of studies to inform policy and practice is important and repeated cross sectional studies provide valuable evidence about changing levels of population mental health and risk factors (e.g. O’Connor *et al*., 2020; Raifman *et al*., 2020). More consistent reporting of sampling frames, repeat survey and the use of validated measures will ensure they make a more meaningful contribution to the evidence base.

There is a paucity of research focussing or reporting on ethnic minorities within populations, children and young people, the bereaved and frontline health and social care staff, which needs to be addressed. Synthesis of findings across studies, and both between and within countries, is confounded by the timing of data collection; differences between studies may be due not only to methodological differences, but also differences in the extent and stringency of public health prevention measures (physical distancing), economic disruption and COVID-19 infection rates in the any population at the time data are collected. A final limitation of the review is that, due to resource limitations, we excluded grey literature (e.g. Fancourt & Steptoe, 2020; National Child Mortality Database, 2020)

### Implications

There is thus far no clear evidence of an increase in suicidal behaviour or self-harm associated with the pandemic, nor with the measures taken to curb the spread of COVID-19, although signals from some repeated population surveys and suicide trend data from Nepal and Japan are concerning. There are suggestions of increased risk in people who have been infected with COVID-19, in line with findings from studies showing increased risk of mental health problems in survivors of COVID-19 ( Taquet *et al*., 2021). Declines in levels of hospital presentation for suicidal behaviour may reflect a real decline in suicidal behaviours early in the pandemic perhaps due to the recognised impact of periods of acute stress / national crisis (e.g. wars) on suicide rates or unmet need in the community, with people cautious about overburdening clinical services or of their own risk of contracting COVID-19 ( John *et al*., 2020a). There is a relative lack of high quality studies to inform prevention in Low and Middle Income Countries and in disadvantaged groups, although studies point to an emerging risk in the latter ( Mitchell & Li., 2021). There are, as yet, no studies that assess the effectiveness of strategies to reduce the risk of suicide deaths, suicidal behaviours, self-harm and suicidal thoughts, resulting from the COVID-19 pandemic; such research is urgently required.

Our living review provides a regular synthesis of the most up-to-date research evidence to guide public health and clinical policy to mitigate the impact of COVID-19 on risk of suicidality. However, the rapid growth of research in this area necessarily makes the reporting of the large volume of included studies brief. Therefore in the future we plan to publish timely updates focussed on specific topics like suicide rates, for instance, or in specific populations such as children and adolescents, those with confirmed COVID-19 or healthcare workers. Our future updates will also focus on studies investigating suicide deaths, suicide attempts and self-harm. We will no longer include studies: with suicidal thoughts and “suicide risk” as outcomes; modelling studies (since these have been superseded by studies based on suicide deaths) and those based on social media posts (because of the lack of evidence for diagnoses and self-selecting biases in respect of who contributes to these).

## Dissemination of information

This living review, along with further updates, will be published via F1000Research. This review was registered on PROSPERO, with ID CRD42020183326. The protocol is available. All further data are publicly available via our Harvard Dataverse repository including all results of the continuous evidence surveillance and screening. Findings from the review will be widely disseminated through conference presentations, policy briefings, peer-reviewed publications, a project website ( https://covid19-suicide-lsr.info/), and traditional and social media outlets.

## Study status

We are currently searching and screening on a daily basis.

### Ethics and dissemination

Since this is a systematic review, ethical approval is not required.

## Data availability

### Underlying data

Harvard Dataverse: Full review data for: "The impact of the COVID-19 pandemic on self-harm and suicidal behaviour: update of living systematic review". https://doi.org/10.7910/DVN/7WZXZK (John & Schmidt, 2020)

This project contains the following underlying data:

- Screening_snapshot.csv (Screening progress for literature published before June 7th)

### Extended data

Harvard Dataverse: Full review data for: "The impact of the COVID-19 pandemic on self-harm and suicidal behaviour: update of living systematic review". https://doi.org/10.7910/DVN/7WZXZK (John & Schmidt, 2020)

This project contains the following extended data:

- LSR update tables and figures.docx (Tables and figures from this publication)
- PRISMA.doc

Data regarding the Protocol are available via our Harvard Dataverse repository for the protocol

Harvard Dataverse: Underlying data for: The impact of the Covid-19 pandemic on suicidal behaviour: a living systematic review protocol. https://doi.org/10.7910/DVN/9JYHLS ( John *et al*., 2020b)

That project contains the following extended data:

- Search.docx (additional information about the searches, including full search strategies)
- Data extraction sheet/ study report
- Figure 1
- Prisma.pdf (the PRISMA-P statement)
- Prospero registration

### Reporting guidelines

Harvard Dataverse: PRISMA checklist for ‘The impact of the COVID-19 pandemic on self-harm and suicidal behaviour: a living systematic review’ https://doi.org/10.7910/DVN/7WZXZK (John & Schmidt, 2020)

Data are available under the terms of the Creative Commons Attribution 4.0 International license (CC-BY 4.0).

## Software availability

The development version of the software for automated searching is available from Github: https://github.com/mcguinlu/COVID_suicide_living.

Archived source code at time of publication: http://doi.org/10.5281/zenodo.3871366 (McGuinness & Schmidt, 2020)

License: MIT

## References

[ref-1] Acharya SR Shin YC Moon DH : COVID-19 outbreak and suicides in Nepal: Urgency of immediate action. *Int J Soc Psychiatry.* 2020; 0020764020963150. 10.1177/0020764020963150 32985316

[ref-2] Ahmed S Khaium MO Tazmeem F : COVID-19 lockdown in India triggers a rapid rise in suicides due to the alcohol withdrawal symptoms: Evidence from media reports. *Int J Soc Psychiatry.* 2020;66(8):827–829. 10.1177/0020764020938809 32586209

[ref-3] Akl EA Meerpohl JJ Elliott J : Living systematic reviews: 4. Living guideline recommendations. *J Clin Epidemiol.* 2017;91:47–53. 10.1016/j.jclinepi.2017.08.009 28911999

[ref-4] Ando M Furuichi M : The impact of COVID-19 employment shocks on suicide and safety net use: An early-stage investigation*. *medRxiv.* 2020. 10.1101/2020.11.16.20232850 PMC8947077 35324902

[ref-5] Anestis MD Bond AE Daruwala SE : Suicidal Ideation Among Individuals Who Have Purchased Firearms During COVID-19. *Am J Prev Med.* 2021;60(3):311–317. 10.1016/j.amepre.2020.10.013 33358551

[ref-6] Bhatia R : Predictions of Covid-19 Related Unemployment On Suicide and Excess Mortality in the United States. *medRxiv.* 2020a. 10.1101/2020.05.02.20089086

[ref-7] Bhatia R : Predictions of Covid-19 Related Unemployment On Suicide and Excess Mortality in the United States. *medRxiv.* 2020b. 10.1101/2020.05.02.20089086v3

[ref-8] Bhuiyan AI Sakib N Pakpour AH : COVID-19-related suicides in Bangladesh due to lockdown and economic factors: case study evidence from media reports. *Int J Ment Health Ad.* 2020;1–6. 10.1007/s11469-020-00307-y 32427168 PMC7228428

[ref-9] Boshra SN Islam MM : The Status and Risk Factors of COVID-19 Related Suicides in Bangladesh. *medRxiv.* 2020a. Reference Source

[ref-10] Boshra SN Islam MM Griffiths MD : The demography and apparent risk factors of COVID-19-related suicides in Bangladesh in a seven-month period of the pandemic. *medRxiv.* 2020b. 10.1101/2020.08.11.20171272

[ref-11] Bray MJC Daneshvari NO Radhakrishnan I : Racial Differences in Statewide Suicide Mortality Trends in Maryland During the Coronavirus Disease 2019 (COVID-19) Pandemic. *JAMA Psychiatry.* 2021;78(4):444–447. 10.1001/jamapsychiatry.2020.3938 33325985 PMC7745133

[ref-12] Buschmann C Tsokos M : Corona-associated suicide - Observations made in the autopsy room. *Leg Med (Tokyo).* 2020a;46:101723. 10.1016/j.legalmed.2020.101723 32526673 PMC7267788

[ref-13] Buschmann C Tsokos M : Corona-associated suicide - Observations made in the autopsy room. *Leg Med (Tokyo).* 2020b;46:101723. 10.1016/j.legalmed.2020.101723 32526673 PMC7267788

[ref-14] Cai Q Feng H Huang J : The mental health of frontline and non-frontline medical workers during the coronavirus disease 2019 (COVID-19) outbreak in China: A case-control study. *J Affect Disord.* 2020;275:210–215. 10.1016/j.jad.2020.06.031 32734910 PMC7329671

[ref-15] Calderon-Anyosa RJC Kaufman JS : Impact of COVID-19 lockdown policy on homicide, suicide, and motor vehicle deaths in Peru. *medRxiv.* 2020. 10.1101/2020.07.11.20150193 PMC7680039 33232687

[ref-16] Capuzzi E di Brita C Caldiroli A : Psychiatric emergency care during Coronavirus 2019 (COVID 19) pandemic lockdown: results from a Department of Mental Health and Addiction of northern Italy. *Psychiatry Res.* 2020;293:113463. 10.1016/j.psychres.2020.113463 32977050 PMC7499069

[ref-17] Chang SS Stuckler D Yip P : Impact of 2008 global economic crisis on suicide: time trend study in 54 countries. *BMJ.* 2013;347:f5239. 10.1136/bmj.f5239 24046155 PMC3776046

[ref-18] Chang YH Chang SS Hsu CY : Impact of Pandemic on Suicide: Excess Suicides in Taiwan During the 1918-1920 Influenza Pandemic. *J Clin Psychiatry.* 2020;81(6):20l13454. 10.4088/JCP.20l13454 32991791

[ref-19] Chen S Jones PB Underwood BR : The early impact of COVID-19 on mental health and community physical health services and their patients' mortality in Cambridgeshire and Peterborough, UK. *J Psychiatr Res.* 2020;131:244–254. 10.1016/j.jpsychires.2020.09.020 33035957 PMC7508053

[ref-20] Cheung Y Chau PH Yip PS : A revisit on older adults suicides and Severe Acute Respiratory Syndrome (SARS) epidemic in Hong Kong. *Int J Geriatr Psychiatry.* 2008;23(12):1231–1238. 10.1002/gps.2056 18500689

[ref-21] Cox J Holden J Sagovsky R : Detection of postnatal depression: Development of the 10-item Edinburgh Postnatal Depression Scale. *Br J Psychiatry.* 1987;150(6):782–786. 10.1192/bjp.150.6.782 3651732

[ref-22] Debowska A Horeczy B Boduszek D : A repeated cross-sectional survey assessing university students' stress, depression, anxiety, and suicidality in the early stages of the COVID-19 pandemic in Poland. *Psychol Med.* 2020;1–4. 10.1017/S003329172000392X 33004087 PMC7556906

[ref-23] Deeks JJ Higgins JPT Altman DG : Analysing data and undertaking meta-analyses. *Cochrane handbook for systematic reviews of interventions.* 2nd ed.: John Wiley & Sons,2019. 10.1002/9781119536604.ch10

[ref-24] Deisenhammer EA Kemmler G : Decreased suicide numbers during the first 6 months of the COVID-19 pandemic. *Psychiatry Res.* 2021;295:113623. 10.1016/j.psychres.2020.113623 33307386

[ref-25] Dragovic M Pascu V Hall T : Emergency department mental health presentations before and during the COVID-19 outbreak in Western Australia. *Australas Psychiatry.* 2020;28(6):627–631. 10.1177/1039856220960673 32961096 PMC7509241

[ref-26] Dsouza DD Quadros S Hyderabadwala ZJ : Aggregated COVID-19 suicide incidences in India: Fear of COVID-19 infection is the prominent causative factor. *Psychiatry Res.* 2020;290:113145. 10.1016/j.psychres.2020.113145 32544650 PMC7832713

[ref-27] Elliott JH Synnot A Turner T : Living systematic review: 1. Introduction-the why, what, when, and how. *J Clin Epidemiol.* 2017;91:23–30. 10.1016/j.jclinepi.2017.08.010 28912002

[ref-28] Elliott JH Turner T Clavisi O : Living systematic reviews: an emerging opportunity to narrow the evidence-practice gap. *PLoS Med.* 2014;11(2):e1001603. 10.1371/journal.pmed.1001603 24558353 PMC3928029

[ref-29] Evans S Mikocka-Walus A Klas A : From " *It Has Stopped Our Lives"* to " *Spending More Time Together Has Strengthened Bonds"*: The Varied Experiences of Australian Families During COVID-19. *Front Psychol.* 2020;11:588667. 10.3389/fpsyg.2020.588667 33192922 PMC7606874

[ref-30] Fancourt D Steptoe A : COVID-19 social study.2020. Reference Source

[ref-31] Faust JS Shah SB Du C : Suicide Deaths during the Stay-at-Home Advisory in Massachusetts. *medRxiv.* 2020. 10.1101/2020.10.20.20215343 PMC7821026 33475750

[ref-32] Finlay I Gilmore I : Covid-19 and alcohol—a dangerous cocktail. *BMJ.* 2020;369:m1987. 10.1136/bmj.m1987 32434792

[ref-33] Fountoulakis KN Pantoula E Siamouli M : Development of the Risk Assessment Suicidality Scale (RASS): a population-based study. *J Affect Disord.* 2012;138(3):449–457. 10.1016/j.jad.2011.12.045 22301115

[ref-34] Gonçalves-Pinho M Mota P Ribeiro J : The Impact of COVID-19 Pandemic on Psychiatric Emergency Department Visits - A Descriptive Study. *Psychiatr Q.* 2021;92(2):621–631. 10.1007/s11126-020-09837-z 32839923 PMC7445073

[ref-35] Griffiths MD Mamun MA : COVID-19 suicidal behavior among couples and suicide pacts: Case study evidence from press reports. *Psychiatry Res.* 2020;289:113105. 10.1016/j.psychres.2020.113105 PMC7229970 33242807

[ref-36] Gunnell D Appleby L Arensman E : Suicide risk and prevention during the COVID-19 pandemic. *Lancet Psychiatry.* 2020;7(6):468–471. 10.1016/S2215-0366(20)30171-1 32330430 PMC7173821

[ref-37] Hamm ME Brown PJ Karp JF : Experiences of American older adults with pre-existing depression during the beginnings of the covid-19 pandemic: A multicity, mixed-methods study. *Am J Geriatr Psychiatry.* 2020;28(9):924–932. 10.1016/j.jagp.2020.06.013 32682619 PMC7305766

[ref-38] Hamza CA Ewing L Heath NL : When social isolation is nothing new: A longitudinal study on psychological distress during COVID-19 among university students with and without preexisting mental health concerns. *Can Psychol.* 2021;62(1):20–30. 10.1037/cap0000255

[ref-39] Hawton K Casey D Bale E : Self-harm during the early period of the COVID-19 Pandemic in England: comparative trend analysis of hospital presentations. *medRxiv.* 2020a. 10.1101/2020.11.25.20238030 PMC7832687 33601744

[ref-40] Hawton K Lascelles K Brand F : Self-harm and the COVID-19 pandemic: a study of factors contributing to self-harm during lockdown restrictions. *medRxiv.* 2020b. 10.1101/2020.12.04.20244129 PMC8561648 33774538

[ref-41] Hawton K Marzano L Fraser L : Reporting on suicidal behaviour and COVID-19-need for caution. *Lancet Psychiatry.* 2021;8(1):15–17. 10.1016/S2215-0366(20)30484-3 33160581

[ref-42] Hernández-Calle D Martínez-Alés G Mediavilla R : Trends in psychiatric emergency department visits due to suicidal ideation and suicide attempts during the CoViD-19 pandemic in Madrid, Spain. *J Clin Psychiatry.* 2020;81(5):20l13419. 10.4088/JCP.20l13419 32898342

[ref-43] Hewson T Green R Shepherd A : The effects of COVID-19 on self-harm in UK prisons. *BJPsych Bull.* 2020;1–3. 10.1192/bjb.2020.83 32669158 PMC7411440

[ref-44] Holmes EA O'Connor RC Perry VH : Multidisciplinary research priorities for the COVID-19 pandemic: a call for action for mental health science. *Lancet Psychiatry.* 2020;7(6):547–560. 10.1016/S2215-0366(20)30168-1 32304649 PMC7159850

[ref-45] Humber N Piper M Appleby L : Characteristics of and trends in subgroups of prisoner suicides in England and Wales. *Psychol Med.* 2011;41(11): 2275–2285. 10.1017/S0033291711000705 21557891

[ref-46] Iob E Steptoe A Fancourt D : Abuse, self-harm and suicidal ideation in the UK during the COVID-19 pandemic. *Br J Psychiatr.* 2020;217(4):543–546. 10.1192/bjp.2020.130 32654678 PMC7360935

[ref-47] Iqbal Y Al Abdulla MA Albrahim S : Psychiatric presentation of patients with acute SARS-CoV-2 infection: a retrospective review of 50 consecutive patients seen by a consultation-liaison psychiatry team. *BJPsych Open.* 2020;6(5):e109. 10.1192/bjo.2020.85 32907692 PMC7484218

[ref-48] Isumi A Doi S Yamaoka Y : Do suicide rates in children and adolescents change during school closure in Japan? The acute effect of the first wave of COVID-19 pandemic on child and adolescent mental health. *Child Abuse Negl.* 2020;110(Pt 2):104680. 10.1016/j.chiabu.2020.104680 32847679 PMC7443207

[ref-49] Jacob S Mwagiru D Thakur I : Impact of societal restrictions and lockdown on trauma admissions during the COVID‐19 pandemic: a single‐centre cross‐sectional observational study. *ANZ J Surg.* 2020;90(11):2227–2231. 10.1111/ans.16307 32894624

[ref-50] Jacobson NC Lekkas D Price G : Flattening the Mental Health Curve: COVID-19 Stay-at-Home Orders Are Associated With Alterations in Mental Health Search Behavior in the United States. *JMIR Ment Health*. in press. 2020. 10.2196/19347 PMC7265799 32459186

[ref-51] Jefsen OH Rohde C NØrremark B : COVID‐19‐related self‐harm and suicidality among individuals with mental disorders. *Acta Psychiatr Scand.* 2020a;142(2):152–153. 10.1111/acps.13214 32659855 PMC7404949

[ref-52] Jefsen OH Rohde C NØrremark B : Editorial Perspective: COVID‐19 pandemic‐related psychopathology in children and adolescents with mental illness. *J Child Psychol Psychiatry.* 2020b. 10.1111/jcpp.13292 32779748 PMC7361472

[ref-54] John A Eyles E Mcguinness LA : The impact of the COVID-19 pandemic on self-harm and suicidal behaviour: protocol for a living systematic review [version 1; peer review: 1 approved, 1 approved with reservations] *F1000Research.* 2020b;9:644. 10.12688/f1000research.24274.1 PMC7871358 33604025

[ref-55] John A Okolie C Eyles E : The impact of the COVID-19 pandemic on self-harm and suicidal behaviour: a living systematic review [version 1; peer review: 1 approved]. *F1000Res.* 2020c;9:1097. 10.12688/f1000research.25522.1 33604025 PMC7871358

[ref-53] John A Pirkis J Gunnell DG : Trends in suicide during the covid-19 pandemic. *BMJ.* 2020a;371:m4352. 10.1136/bmj.m4352 33184048

[ref-57] Joiner TE Pfaff JJ Acres JG : A brief screening tool for suicidal symptoms in adolescents and young adults in general health settings: reliability and validity data from the Australian National General Practice Youth Suicide Prevention Project. *Behav Res Ther.* 2002;40(4):471–81. 10.1016/s0005-7967(01)00017-1 12008659

[ref-56] Jolly TS Batchelder E Baweja R : Mental Health Crisis Secondary to COVID-19-Related Stress: A Case Series From a Child and Adolescent Inpatient Unit. *Prim care companion CNS disord.* 2020;22(5):20l02763. 10.4088/PCC.20l02763 32942348

[ref-58] Kapilan N : Suicides cases among nurses in India due to COVID-19 and possible prevention strategies. *Asian J Psychiatr.* 2020;54:102434. 10.1016/j.ajp.2020.102434 33271714 PMC7553098

[ref-59] Kar SK Arafat SY Ransing R : Repeated celebrity suicide in India during COVID-19 crisis: An urgent call for attention. *Asian J Psychiatr.* 2020;53:102382. 10.1016/j.ajp.2020.102382 32882671 PMC7451094

[ref-61] Karakasi MV Kevrekidis DP Pavlidis P : The Role of the SARS-CoV-2 Pandemic on Suicide Rates: Preliminary Study in a Sample of the Greek Population. *Am J Forensic Med Pathol.* 2021;42(1):99-100. 10.1097/PAF.0000000000000598 33181519 PMC7870042

[ref-60] Karakasi MV Zaoutsou A Theofilidis A : The impact of the SARS‐CoV‐2 pandemic on psychiatric emergencies in northern Greece: preliminary study on a sample of the Greek population. *Psychiatry Clin Neurosci.* 2020a;74(11):613–615. 10.1111/pcn.13136 32827321 PMC7461518

[ref-62] Kavoor AR Chakravarthy K John T : Remote consultations in the era of COVID-19 pandemic: Preliminary experience in a regional Australian public acute mental health care setting. *Asian J Psychiatr.* 2020;51:102074. 10.1016/j.ajp.2020.102074 32294583 PMC7195079

[ref-63] Kawohl W Nordt C : COVID-19, unemployment, and suicide. *Lancet Psychiatry.* 2020;7(5):389–390. 10.1016/S2215-0366(20)30141-3 32353269 PMC7185950

[ref-64] Ketphan O Juthamanee S Racal SJ : The mental health care model to support the community during the COVID-19 pandemic in Thailand. *Belitung Nurs J.* 2020;6:152–156. 10.33546/bnj.1193

[ref-66] Khan MM Ahmed A Khan SR : Female suicide rates in Ghizer, Pakistan. *Suicide Life Threat Behav.* 2009;39(2):227–230. 10.1521/suli.2009.39.2.227 19527163

[ref-65] Khan MM Hyder AA : Suicides in the developing world: Case study from Pakistan. *Suicide Life Threat Behav.* 2006;36(1):76–81. 10.1521/suli.2006.36.1.76 16676628

[ref-67] Killgore WD Cloonan SA Taylor EC : Trends in suicidal ideation over the first three months of COVID-19 lockdowns. *Psychiatry Res.* 2020a;293:113390. 10.1016/j.psychres.2020.113390 32835926 PMC7430225

[ref-68] Kim AM : The short-term impact of the COVID-19 outbreak on suicides in Korea. *Psychiatry Res.* 2021;295:113632. 10.1016/j.psychres.2020.113632 33338860 PMC7718107

[ref-69] Kisely S Warren N McMahon L : Occurrence, prevention, and management of the psychological effects of emerging virus outbreaks on healthcare workers: rapid review and meta-analysis. *BMJ.* 2020;369;m1642. 10.1136/bmj.m1642 32371466 PMC7199468

[ref-70] Klonsky ED Glenn CR : Assessing the functions of non-suicidal self-injury: Psychometric properties of the Inventory of Statements About Self-injury (ISAS). *J Psychopathol Behav Assess.* 2009;31(3):215–219. 10.1007/s10862-008-9107-z 29269992 PMC5736316

[ref-71] Knipe D Evans H Marchant A : Mapping population mental health concerns related to COVID-19 and the consequences of physical distancing: a Google trends analysis [version 2; peer review: 2 approved] *Wellcome Open Res.* 2020;5:82. 10.12688/wellcomeopenres.15870.2 32671230 PMC7331103

[ref-72] Lersch KM : COVID-19 and Mental Health: An Examination of 911 Calls for Service. *Policing: A Journal of Policy and Practice.* 2020;14(4):1112–1126. 10.1093/police/paaa049 PMC7454910 40504201

[ref-73] Leske S Kõlves K Crompton D : Real-time suicide mortality data from police reports in Queensland, Australia, during the COVID-19 pandemic: an interrupted time-series analysis. *Lancet Psychiatry.* 2021;8(1):58–63. 10.1016/S2215-0366(20)30435-1 33212023 PMC7836943

[ref-74] Low DM Rumker L Talkar T : Natural Language Processing Reveals Vulnerable Mental Health Support Groups and Heightened Health Anxiety on Reddit During COVID-19: Observational Study. *J Med Internet Res.* 2020;22(10):e22635. 10.2196/22635 32936777 PMC7575341

[ref-75] Mahase E : Covid-19: EU states report 60% rise in emergency calls about domestic violence. *BMJ.* 2020;369:m1872. 32393463 10.1136/bmj.m1872

[ref-76] Mamun MA Chandrima RM Griffiths MD : Mother and son suicide pact due to COVID-19-related online learning issues in Bangladesh: An unusual case report. *Int J Ment Health Addict.* 2020a;1–4. 10.1007/s11469-020-00362-5 32837439 PMC7340761

[ref-77] Mamun MA Syed NK Griffiths MD : Indian celebrity suicides before and during the COVID-19 pandemic and their associated risk factors: Evidence from media reports. *J Psychiatr Res.* 2020b;131:177–179. 10.1016/j.jpsychires.2020.09.002 32979693 PMC7477604

[ref-78] Mamun MA Ullah I : COVID-19 suicides in Pakistan, dying off not COVID-19 fear but poverty? – The forthcoming economic challenges for a developing country. *Brain Behav Immun.* 2020;87:163–166. 10.1016/j.bbi.2020.05.028 32407859 PMC7212955

[ref-79] McAndrew J O’Leary J Cotter D : Impact of initial COVID-19 restrictions on psychiatry presentations to the emergency department of a large academic teaching hospital. *Ir J Psychol Med.* 2020;1–8. 10.1017/ipm.2020.115 32996441 PMC7711497

[ref-80] McIntyre A Tong K McMahon E : Covid-19 and its effect on emergency presentations to a tertiary hospital with self-harm in Ireland. *Ir J Psychol Med.* 2020;1–7. 10.1017/ipm.2020.116 32993833 PMC7711341

[ref-81] McIntyre RS Lee Y : Preventing suicide in the context of the COVID-19 pandemic. *World Psychiatry.* 2020a;19(2):250–251. 10.1002/wps.20767 32394579 PMC7214950

[ref-82] McIntyre RS Lee Y : Projected increases in suicide in Canada as a consequence of COVID-19. *Psychiatry Res.* 2020b;290:113104. 10.1016/j.psychres.2020.113104 32460184 PMC7236718

[ref-83] Mitchell TO Li L : State-Level Data on Suicide Mortality During COVID-19 Quarantine: Early Evidence of a Disproportionate Impact on Racial Minorities. *Psychiatry Res.* 2021;295:113629. 10.1016/j.psychres.2020.113629 33290944

[ref-84] Moher D Shamseer L Clarke M : Preferred reporting items for systematic review and meta-analysis protocols (PRISMA-P) 2015 statement. *Syst Rev.* 2015;4(1):1. 10.1186/2046-4053-4-1 25554246 PMC4320440

[ref-85] Moser DA Glaus J Frangou S : Years of life lost due to the psychosocial consequences of COVID-19 mitigation strategies based on Swiss data. *Eur Psychiatry.* 2020;63(1):e58. 10.1192/j.eurpsy.2020.56 32466820 PMC7303469

[ref-86] Morgan R Sterne JA Higgins JP : A new instrument to assess Risk of Bias in Non-randomised Studies of Exposures (ROBINS-E): Application to studies of environmental exposure *Global Evidence Summit.* Cape Town.2017. Reference Source

[ref-87] Morin CM Belleville G Bélanger L : The Insomnia Severity Index: psychometric indicators to detect insomnia cases and evaluate treatment response. *Sleep.* 2011;34(5):601–608. 10.1093/sleep/34.5.601 21532953 PMC3079939

[ref-88] Nalleballe K Onteddu SR Sharma R : Spectrum of neuropsychiatric manifestations in COVID-19. *Brain Behav Immun.* 2020;88:71–74. 10.1016/j.bbi.2020.06.020 32561222 PMC7297688

[ref-89] National Child Mortality Database: Child Suicide Rates during the COVID-19 Pandemic in England: Real-time Surveillance.2020. Reference Source

[ref-90] Nock MK Holmberg EB Photos VI : Self-Injurious Thoughts and Behaviors Interview: Development, reliability, and validity in an adolescent sample. *Psychol Assess.* 2007;19(3):309–317. 10.1037/1040-3590.19.3.309 17845122

[ref-91] O'Connor RC Wetherall K Cleare S : Mental health and well-being during the COVID-19 pandemic: longitudinal analyses of adults in the UK COVID-19 Mental Health & Wellbeing study. *Br J Psychiatry.* 2020;1–8. 10.1192/bjp.2020.212 33081860 PMC7684009

[ref-92] Olding J Zisman S Olding C : Penetrating trauma during a global pandemic:changing patterns in interpersonal violence, self-harm and domestic violence in the Covid-19 outbreak. *Surgeon.* 2021;19(1):e9–e13. 10.1016/j.surge.2020.07.004 32826157 PMC7392113

[ref-93] Pierce M McManus S Jessop C : Says who? The significance of sampling in mental health surveys during COVID-19. *Lancet Psychiatry.* 2020;7(7):567–568. 10.1016/S2215-0366(20)30237-6 32502467 PMC7266586

[ref-94] Pignon B Gourevitch R Tebeka S : Dramatic reduction of psychiatric emergency consultations during lockdown linked to COVID-19 in Paris and suburbs. *MedRxiv.* 2020a. 10.1101/2020.05.19.20095901 PMC7361336 32609417

[ref-95] Pignon B Gourevitch R Tebeka S : Dramatic reduction of psychiatric emergency consultations during lockdown linked to COVID-19 in Paris and suburbs. *Psychiatry Clin Neurosci.* 2020b;74:557–559. 10.1111/pcn.13104 32609417 PMC7361336

[ref-96] Pirnia B Dezhakam H Pirnia K : Grief of COVID-19 is a mental contagion, first family suicide in Iran. *Asian J Psychiatr.* 2020;54:102340. 10.1016/j.ajp.2020.102340 32777756 PMC7834061

[ref-97] Pokhrel S Sedhai YR Atreya A : An increase in suicides amidst the coronavirus disease 2019 pandemic in Nepal. *Med Sci Law.* 2021;61(2):161–162. 10.1177/0025802420966501 33036544

[ref-98] Poudel K Subedi P : Impact of COVID-19 pandemic on socioeconomic and mental health aspects in Nepal. *Int J Soc Psychiatry.* 2020;66(8):748–755. 10.1177/0020764020942247 32650687 PMC7443960

[ref-99] Qin P Mehlum L : National observation of death by suicide in the first 3 months under COVID-19 pandemic. *Acta Psychiatr Scand.* 2021;143(1):92–93. 10.1111/acps.13246 33111325

[ref-100] Radeloff D Papsdorf R Uhlig K : Trends in suicide rates during the COVID-19 pandemic restrictions in a major German city. *medRxiv.* 2020. 10.1101/2020.10.21.20187419 PMC7889841 33461639

[ref-101] Radeloff D Papsdorf R Uhlig K : Trends in suicide rates during the COVID-19 pandemic restrictions in a major German city. *Epidemiol Psychiatr Sci.* 2021;30:E16. 10.1017/S2045796021000019 33461639 PMC7889841

[ref-102] Rahman A Plummer V : COVID-19 related suicide among hospital nurses; case study evidence from worldwide media reports. *Psychiatry Res.* 2020;291:113272. 10.1016/j.psychres.2020.113272 32886958 PMC7331553

[ref-103] Raifman J Ettman CK Dean L : Economic precarity, social isolation, and suicidal ideation during the COVID-19 pandemic. *medRxiv.* 2020. 10.1101/2020.10.05.20205955 PMC9668199 36383566

[ref-104] Rajkumar RP : Suicides related to the COVID-19 outbreak in India: a pilot study of media reports. *Asian J Psychiatr.* 2020;53:102196. 10.1016/j.ajp.2020.102196 32534434 PMC7274089

[ref-105] Rajput K Sud A Rees M : Epidemiology of trauma presentations to a major trauma centre in the North West of England during the COVID-19 level 4 lockdown. *Eur J Trauma Emerg Surg.* 2020;1–6. 10.1007/s00068-020-01507-w 32997167 PMC7525754

[ref-106] Rana U : Elderly Suicides in India: An Emerging Concern during COVID-19 Pandemic. *Int Psychogeriatr.* 2020;32(10):1251–1252. 10.1017/S1041610220001052 32487275 PMC7322164

[ref-107] Reger MA Stanley IH Joiner TE : Suicide Mortality and Coronavirus Disease 2019—A Perfect Storm? *JAMA Psychiatry.* 2020;77(11):1093–1094. 10.1001/jamapsychiatry.2020.1060 32275300

[ref-108] Rhodes HX Petersen K Biswas S : Trauma trends during the initial peak of the COVID-19 pandemic in the midst of lockdown: experiences from a rural trauma center. *Cureus.* 2020;12(8):e9811. 10.7759/cureus.9811 32953322 PMC7494409

[ref-109] Rivlin A Hawton K Marzano L : Psychiatric disorders in male prisoners who made near-lethal suicide attempts: case–control study. *Br J Psychiatry.* 2010;197(4):313–319. 10.1192/bjp.bp.110.077883 20884955

[ref-110] Rohde C Jefsen OH Nørremark B : Psychiatric Symptoms Related to the COVID-19 Pandemic. *medRxiv.* 2020. 10.1101/2020.04.16.20067744 32434604

[ref-111] Rück C Mataix-cols D Malki K : Will the COVID-19 pandemic lead to a tsunami of suicides? A Swedish nationwide analysis of historical and 2020 data. *medRxiv.* 2020. 10.1101/2020.12.10.20244699

[ref-112] Sade S Sheiner E Wainstock T : Risk for depressive symptoms among hospitalized women in high-risk pregnancy units during the COVID-19 pandemic. *J Clin Med.* 2020;9(8):2449. 10.3390/jcm9082449 32751804 PMC7464613

[ref-113] Saha K Torous J Caine ED : Psychosocial Effects of the COVID-19 Pandemic: Large-scale Quasi-Experimental Study on Social Media. *J Med Internet Res.* 2020;22(11):e22600. 10.2196/22600 33156805 PMC7690250

[ref-114] Sahoo S Rani S Parveen S : Self-harm and COVID-19 Pandemic: An emerging concern – A report of 2 cases from India. *Asian J Psychiatr.* 2020;51:102104. 10.1016/j.ajp.2020.102104 32325391 PMC7161515

[ref-115] Sakelliadis EI Katsos KD Zouzia EI : Impact of Covid-19 Lockdown on Characteristics of Autopsy Cases in Greece. Comparison between 2019 and 2020. *Forensic Sci Int.* 2020;313:110365. 10.1016/j.forsciint.2020.110365 32563134 PMC7291972

[ref-116] Sheridan DC Cloutier R Johnson K : Where have all the emergency paediatric mental health patients gone during COVID-19? *Acta Paediatr.* 2021;110(2):598–599. 10.1111/apa.15537 32799399 PMC7461447

[ref-117] Shoib S Nagendrappa S Grigo O : Factors associated with COVID-19 outbreak-related suicides in India. *Asian J Psychiatr.* 2020;53:e102223. 10.1016/j.ajp.2020.102223 32574941

[ref-118] Singh R Baral KP Mahato S : An urgent call for measures to fight against increasing suicides during COVID-19 pandemic in Nepal. * Asian J Psychiatr.* 2020;54:102259. 10.1016/j.ajp.2020.102259 32619837 PMC7305514

[ref-119] Sinyor M Spittal MJ Niederkrotenthaler T : Changes in Suicide and Resilience-related Google Searches during the Early Stages of the COVID-19 Pandemic. *Can J Psychiatry.* 2020;65(10):741–743. 10.1177/0706743720933426 32524848 PMC7502879

[ref-120] Smalley CM Malone DA Meldon SW : The impact of COVID-19 on suicidal ideation and alcohol presentations to emergency departments in a large healthcare system. *Am J Emerg Med.* in press 2021.41:237–238. 10.1016/j.ajem.2020.05.093 32505472 PMC7263212

[ref-121] Son C Hegde S Smith A : Effects of COVID-19 on college students’ mental health in the United States: Interview survey study. *J Med Internet Res.* 2020;22(9):e21279. 10.2196/21279 32805704 PMC7473764

[ref-122] Sterne JA Hernán MA Reeves BC : ROBINS-I: a tool for assessing risk of bias in non-randomised studies of interventions. *BMJ.* 2016;355:i4919. 10.1136/bmj.i4919 27733354 PMC5062054

[ref-123] Stuckler D Basu S Suhrcke M : The public health effect of economic crises and alternative policy responses in Europe: an empirical analysis. *Lancet.* 2009;374(9686):315–323. 10.1016/S0140-6736(09)61124-7 19589588

[ref-124] Sueki H : Development of a short form of the suicidal ideation scale. *Suicide Prevention & CrisisIntervention.* 2019;39(2):94–101. Reference Source

[ref-125] Sueki H Ueda M : Short-term effect of the COVID-19 pandemic on suicidal ideation: A prospective cohort study. *PsyArXiv.* 2020. 10.31234/osf.io/3jevh

[ref-126] Syed NK Griffiths MD : Nationwide suicides owing to alcohol withdrawal symptoms during COVID-19 pandemic: A review of cases from media reports. *J Psychiatr Res.* 2020;130:289–291. 10.1016/j.jpsychires.2020.08.021 32866677 PMC7438040

[ref-127] Tanaka T Okamoto S : Suicide during the COVID-19 pandemic in Japan. *medRxiv.* 2020. 10.1101/2020.08.30.20184168

[ref-128] Tanaka T Okamoto S : Increase in suicide following an initial decline during the COVID-19 pandemic in Japan. *Nat Hum Behav.* 2021;5(2):229–238. 10.1038/s41562-020-01042-z 33452498

[ref-129] Taquet M Luciano S Geddes JR : Bidirectional associations between COVID-19 and psychiatric disorder: retrospective cohort studies of 62 354 COVID-19 cases in the USA. *Lancet Psychiatry.* 2021;8(2):130–140. 10.1016/S2215-0366(20)30462-4 33181098 PMC7820108

[ref-130] Thakur V Jain A : COVID 2019-suicides: A global psychological pandemic. *Brain Behav Immun.* in press 2020;88:952–953. 10.1016/j.bbi.2020.04.062 32335196 PMC7177120

[ref-131] Titov N Staples L Kayrouz R : Rapid report: Early demand, profiles and concerns of mental health users during the coronavirus (COVID-19) pandemic. *Internet Interv.* 2020;21:100327. 10.1016/j.invent.2020.100327 32537424 PMC7262525

[ref-132] Turecki G Brent DA Gunnell D : Suicide and suicide risk. *Nat Rev Dis Primers.* 2019;5(1):74. 10.1038/s41572-019-0121-0 31649257

[ref-133] Ueda M Nordström R Matsubayashi T : Suicide and mental health during the COVID-19 pandemic in Japan. *J Public Health (Oxf).* 2021;fdab113. 10.1093/pubmed/fdab113 33855451 PMC8083330

[ref-134] Valdés-Florido MJ López-Díaz Á Palermo-Zeballos FJ : Reactive psychoses in the context of the COVID-19 pandemic: clinical perspectives from a case series. * Rev Psiquiatr Salud Ment.* 2020;13(2):90–94. 10.1016/j.rpsm.2020.04.009 38620329 PMC7183984

[ref-135] Vandoros S Theodorikakou O Katsadoros K : No evidence of increase in suicide in Greece during the first wave of Covid-19. *medRxiv.* 2020. 10.1101/2020.11.13.20231571

[ref-136] Walker LE Heaton HA Monroe RJ : Impact of the SARS-CoV-2 Pandemic on Emergency Department Presentations in an Integrated Health System. * Mayo Clin Proc.* 2020;95(11):2395–2407. 33153630 10.1016/j.mayocp.2020.09.019 PMC7501771

[ref-137] Wang R Li J Mei J : Research on the suicide risk, sleep, psychological status and influencing factors of coronavirus disease 2019 patients.Lanzhou University/CNKI ID: czh-359.2020a. Reference Source

[ref-138] Wang Y Hu Z Feng Y : Changes in network centrality of psychopathology symptoms between the COVID-19 outbreak and after peak. *Mol Psychiatry.* 2020b;25(12):3140–3149. 10.1038/s41380-020-00881-6 32929212 PMC7488637

[ref-139] Wasserman IM : The impact of epidemic, war, prohibition and media on suicide: United States, 1910-1920. *Suicide Life Threat Behav.* 1992;22(2):240–254. 1626335

[ref-140] Winkler P Formanek T Mlada K : Increase in prevalence of current mental disorders in the context of COVID-19: analysis of repeated nationwide cross-sectional surveys. * Epidemiol Psychiatr Sci.* 2020;29:e173. 10.1017/S2045796020000888 32988427 PMC7573458

[ref-141] Wong TW Gao Y Tam WWS : Anxiety among university students during the SARS epidemic in Hong Kong. *Stress Health.* 2007;23(1):31–35. 10.1002/smi.1116

[ref-142] Worldometers: Covid-19 Coronavirus Pandemic.2020; [Accessed 07/06/2020]. Reference Source

[ref-143] Wu C Hu X Song J : Mental health status and related influencing factors of COVID-19 survivors in Wuhan, China. *Clin Transl Med.* 2020a;10(2):e52. 10.1002/ctm2.52 32508037 PMC7300592

[ref-144] Wu Y Zhang C Liu H : Perinatal depressive and anxiety symptoms of pregnant women during the coronavirus disease 2019 outbreak in China. *Am J Obstet Gynecol.* 2020b;223(2):240.e1–240.e9. 10.1016/j.ajog.2020.05.009 32437665 PMC7211756

[ref-145] Zhao Q Hu C Feng R : Investigation of the mental health of patients with novel coronavirus pneumonia. *Chinese Journal of Neurology.* 2020; (12):E003–E003. 10.3760/cma.j.cn113694-20200220-00102

[ref-146] Zhang L Zhang D Fang J : Assessment of mental health of Chinese primary school students before and after school closing and opening during the COVID-19 pandemic. *JAMA Netw Open.* 2020;3(9):e2021482. 10.1001/jamanetworkopen.2020.21482 32915233 PMC7489803

[ref-147] Zortea TC Brenna CTA Joyce M : The Impact of Infectious Disease-Related Public Health Emergencies on Suicide, Suicidal Behavior, and Suicidal Thoughts. *Crisis.* 2020;1–14. 10.1027/0227-5910/a000753 33063542 PMC8689932

