# Supplementary material for: The impact of the COVID-19 pandemic on self-harm and suicidal behaviour: update of living systematic review
Source: F1000Res. 2021 Jun 17;9:1097. Originally published 2020 Sep 4. [Version 2] doi: 10.12688/f1000research.25522.2 (PMC7871358; doi:10.12688/f1000research.25522.2)
Supplement: Peer review history

# Peer review: Reviewer response for version 1

**DOI:** 10.5256/f1000research.28166.r77902

**Published:** 2021-02-08

**Authors:** Gonzalo Martinez-Ales

**Affiliations:** 1 Mailman School of Public Health, Columbia University, New York, NY, USA

This manuscript is a great scientific contribution. The main strength of the manuscript (that it builds on a remarkable effort -- their living systematic review) goes hand in hand with the most important limitation (the period included in the particular iteration that is under consideration for publication). I would like to thank the authors for such a great addition to science (the living systematic review) and express my admiration. Next, I expand on these observations.

 The introduction is right on target and reads well. A reference to recent increases in gun purchases in the US (e.g., https://www.businessinsider.com/gun-sales-boom-2020-background-checks-hit-record-highs-2021-1). Methods are sound. Results are concise and informative. The tables are particularly interesting and we congratulate the authors on the table including modelling studies as it conveys the most important information easily. The discussion also reads well and adjusts well to the findings.

 There is, however, a major limitation to this study that authors may want to address: the limited period of time included. This iteration of the review stopped including papers by July 7th, roughly 4 months after the pandemic hit Western countries for the first time. Notably, this review would have been of great interest if published over the summer. Several research reports (and important grey literature) have become public in the meantime, some adding to the evidence reviewed here without notably changing the overarching results but enhancing their reliability (and probably creating the necessary ground for a quantitative summary or a meta-analysis) and, more importantly, some creating groundbreaking evidence that may change the conclusion of this review (such as the Nature Comms paper by Tanaka and Okamoto using data from Japan to show an initial dip and subsequent increase in suicide rates in Japan). 

 See some key recent key additions to the literature as an example:

https://www.nature.com/articles/s41562-020-01042-z ^1^

https://www.medrxiv.org/content/10.1101/2020.11.13.20231571v1 ^2^

https://www.medrxiv.org/content/10.1101/2020.10.06.20207530v5 ^3^

https://www.medrxiv.org/content/10.1101/2020.10.21.20187419v1 ^4^

https://www.medrxiv.org/content/10.1101/2020.10.20.20215343v1 ^5^

 The impact of this profound and sound review is somewhat limited by the period included: readers should resort to the authors’ ongoing live review.

Are the rationale for, and objectives of, the Systematic Review clearly stated?

Yes

Is the statistical analysis and its interpretation appropriate?

Not applicable

Are sufficient details of the methods and analysis provided to allow replication by others?

Yes

Are the conclusions drawn adequately supported by the results presented in the review?

Yes

Reviewer Expertise:

Psychiatric epidemiology.

I confirm that I have read this submission and believe that I have an appropriate level of expertise to confirm that it is of an acceptable scientific standard, however I have significant reservations, as outlined above.

## References

[1] : Increase in suicide following an initial decline during the COVID-19 pandemic in Japan. *Nat Hum Behav* .2021; 10.1038/s41562-020-01042-z 10.1038/s41562-020-01042-z 33452498

[2] : No evidence of increase in suicide in Greece during the first wave of Covid-19. *medRxiv* .2020; 10.1101/2020.11.13.20231571 10.1101/2020.11.13.20231571

[3] : Suicide and mental health during the COVID-19 pandemic in Japan. *medRxiv* .2020; 10.1101/2020.10.06.20207530 10.1101/2020.10.06.20207530

[4] : Trends in suicide rates during the COVID-19 pandemic restrictions in a major German city. *medRxiv* .2020; 10.1101/2020.10.21.20187419 10.1101/2020.10.21.20187419 PMC788984133461639

[5] : Suicide Deaths during the Stay-at-Home Advisory in Massachusetts. *medRxiv* .2020; 10.1101/2020.10.20.20215343 10.1101/2020.10.20.20215343

# Peer review: Reviewer response for version 1

**DOI:** 10.5256/f1000research.28166.r75857

**Published:** 2021-01-11

**Authors:** Kimberly A Van Orden

**Affiliations:** 1 Department of Psychiatry, University of Rochester Medical Center, Rochester, New York, USA

This article provides a review of empirical studies on suicide ideation, behavior, and deaths as related to the COVID-19 pandemic (up to June 2020). Given prior data linking disasters and crises more generally, and pandemics specifically, to changes in suicide rates, describing any changes in suicide rates (as well as suicide ideation and non-fatal behavior) due to the COVID-19 pandemic could contribute to suicide prevention science and promote public health efforts to save lives. A key strength of this paper is its design as a ‘living review’ that will be updated every six months as more data is available. Another strength is the transparent reporting on search methods and strategies.

 A limitation of the paper is inadequate attention to study quality in the analysis and interpretation of findings. I will give several examples. First, the authors report that they used a formal tool to assess the risk of bias for epidemiological or clinical trial design studies, but do not report findings from these assessments; given that many papers included in the review were not peer reviewed, it seems especially useful for the authors to use such assessments of study quality to guide their review and to ‘weight’ findings from these studies in their analysis. Second, the degree of methodological rigor could be assessed for all studies, not just those with epidemiology/clinical trial designs and the authors should consider doing so. Third, the authors indicate in the primary table that letters to the editor were ‘probably not peer reviewed.’ Given that this information could be verified by contacting the journals, this would be a useful strategy to bolster findings from this review. Fourth, when the authors describe the findings, they do not differentiate between findings that appear methodologically-sound versus those that may not be, thus negating one of the most useful features of review papers for readers.

 Another limitation of the paper is that it provides relatively little synthesis or conclusions, which is a key function of review papers, as opposed to a database that contains a listing of available studies. The discussion section includes more of a summary of what studies examined (and did not examine) as opposed to a synthesis of findings. The authors do not provide a nuanced discussion of the fact that these studies come from numerous countries around the world and what addressing this issue could potentially tell us about possible variability in suicide rates around the world. They do not discuss limitations with sampling that appeared across studies (e.g., generalizability of online platforms like M-Turk). In the discussion section, the authors conclude that “a marked improvement in the quality of design, methods, and reporting in future studies is needed.” This may be accurate, but I do not think it is an especially useful statement to guide the field. A more useful set of statements might involve a synthesis of methodological strengths and weaknesses as well as a discussion on strategies that can be taken going forward to address these weaknesses. The authors do not posit further implications; this may be accurate—that nothing else can be concluded right now—but in that case, perhaps the paper is premature.

 The authors should provide additional details on the methods used for the review process to construct Tables 1-4. In particular, for the column labeled ‘Conclusions,’ presumably, this refers to conclusions made by the authors of the original papers? This should be stated explicitly. Did the authors of this review include all conclusions made by the authors of the original studies in the table or did they select ones deemed most useful?  How did the authors of this review select the limitations and comments included in the final column? Some of the comments included in that final column appear opinion-based and are not supported by data from the papers (e.g., prevalence is “surprisingly low” or these data “cannot be interpreted” and “usefully”).

 For future updates, the authors should consider providing dates for data collection in their tables given that the timing of when studies are conducted may moderate findings, given the variability in length of physical distancing, amount of economic disruption, and the number of deaths due to COVID-19.

Are the rationale for, and objectives of, the Systematic Review clearly stated?

Yes

Is the statistical analysis and its interpretation appropriate?

Not applicable

Are sufficient details of the methods and analysis provided to allow replication by others?

No

Are the conclusions drawn adequately supported by the results presented in the review?

Partly

Reviewer Expertise:

Suicide prevention

I confirm that I have read this submission and believe that I have an appropriate level of expertise to confirm that it is of an acceptable scientific standard, however I have significant reservations, as outlined above.

# Peer review: Reviewer response for version 1

**DOI:** 10.5256/f1000research.28166.r71350

**Published:** 2020-09-22

**Authors:** Lakshmi Vijayakumar

**Affiliations:** 1 Department of Psychiatry, VHS SNEHA (Suicide Prevention Agency), Chennai, Tamil Nadu, India

- This is a much needed study during the pandemic which is constantly evolving with many ramifications.
- In the category of what are the effects of other exposures, suicide by railways can be added. In fact there a likely reduction of railway suicides. The other addition could be the impact of working from home, change in workplace etc.
- The authors have righty pointed out that the studies are from newspaper reports, non-representative samples and cross-sectional, hence the generalizability of these findings are limited.
- One is not sure of when studies using proxy data like newspaper data are included, and why Google trend studies are not included.
- The paper is a call for more robust well-designed studies to understand the association between the pandemic and suicidal behaviour.

Are the rationale for, and objectives of, the Systematic Review clearly stated?

Yes

Is the statistical analysis and its interpretation appropriate?

Yes

Are sufficient details of the methods and analysis provided to allow replication by others?

Yes

Are the conclusions drawn adequately supported by the results presented in the review?

Yes

Reviewer Expertise:

suicide research

I confirm that I have read this submission and believe that I have an appropriate level of expertise to confirm that it is of an acceptable scientific standard.
